# Supplementary material for: Evaluation of the performance of nucleic acid amplification tests (NAATs) in detection of chlamydia and gonorrhoea infection in vaginal specimens relative to patient infection status: a systematic review
Source: BMJ Open. 2019 Jan 17;9(1):e022510. doi: 10.1136/bmjopen-2018-022510 (PMC6340625; doi:10.1136/bmjopen-2018-022510)
Supplement: Supplementary data [file bmjopen-2018-022510supp001.pdf]

Supplemental File for

Evaluation of the performance of nucleic acid amplification tests (NAATs) in detection of chlamydia and gonorrhoea infection in vaginal specimens relative to patient infection status: a systematic review

Minttu M. Rönn, Louise Mc Grath-Lone, Bethan Davies, Janet Wilson, Helen Ward

Search strategy

The following Boolean searches were performed on OvidSP on 8 October 2013 and an update on 3 October 2017. We searched all fields (af) using Ovid MEDLINE (1947-present) and EMBASE (1946-present) databases. We combined the individual searches with AND in the following manner:

(chlamydia OR “chlamydia trachomatis”).af OR (gonorrh?ea OR “Neisseria gonorrhoeae”).af

AND

(vagina$.af OR vulvo-vagina$).af

AND

(urine OR endovervi$ OR cervi$).af

AND

(NAAT OR “nucleic acid amplification” OR (PCR OR “polymerase chain reaction”) OR (TMA OR “transcription mediated amplification OR “transcription-mediated amplification”) OR (SDA or “Strand displacement amplification”) OR (LCR or “ligase chain reaction”)).af

AND

(sensitivity OR specificity OR performance).af

AND

(diagnos$ OR screen$ OR test OR detect$).af

Table S1.Studies which were excluded based on the full article (n=81).

Does not have >1 NAAT per site for all samples for CT (or 1 NAAT plus culture for NG)		Performace calculation not based on PIS	Duplicate data
Bakken et al., 2005 (1)	Schoeman et al., 2012 (2)	Jaton et al., 2006 (3)	Andrews, 2009 (4)
Behets et al., 2002 (5)	Schmitt et al, 2014 (6)	Le Roy et al., 2013 (7)	
Berwald et al., 2009(8)	Shrier et al., 2004 (9)	Shalepo et al., 2006 (10)	**Study population all infected**
Black et al., 2009 (11)	Skidmore et al., 2006 (12)	Schachter et al., 2003 (13)	Bachmann et al., 2002 (14)
Carder et al., 1999 (15)	Stewart et al., 2012 (16)	Samra et al., 2011 (17)	
Chandeying et al., 2004 (18)	Sturm et al., 2004 (19)	Upton et al., 2013 (20)	
Cheng et al. 2014 (21)	Tanaka et al., 1998 (22)	Vahidnia et al., 2014 (23)	
Dietrich et al., 2010 (24)	Tanaka et al., 2000 (25)	Yu et al., 2016(26)	**No vaginal samples**
Domeika & Drulyte, 2000 (27)	Taylor et al., 2011 (28)	Zauli et al., 2013 (29)	
Domeika et al.,1999 (30)	Thomas et al., 1998 (31)		Carroll et al. 1998 (32)
Falk et al., 2010 (33)	Thomas et al., 2001 (34)		Lee et al., 2013 (35)
Fang et al., 2008 (36)	Van Der Pol, 2012 (37)		Nunez-Forero et al. 2015 (38)
Fernandez et al., 2016 (39)	Van Der Pol et al., 2012 (28)		
Garrow et al., 2002 (40)	Van Der Pol et al., 2013 (41)	**No primary data reported**	
Gaydos et al., 2013 (42)	Van Der Pol et al., 2015 (43)	Chernesky & Jang, 2006 (44)	**Only vaginal samples**
Gokral et al., 2005 (45)	Van Der Pol et al., 2017 (46)	Fong & Christian, 2012 (47)	Gaydos et al. 2002 (48)
		Gaydos et al., 2014 (49)	
Hjelm et al., 2001 (50)	Van Dommelen et al., 2011 (51)	Meyer, 2012 (52)	
Hsieh et al., 2003 (53)	Wiesenfeld et al., 1996 (54)	Sautter et al., 2009 (55)	

Keane et al., 2007 (56)	Witkin et al., 1996 (57)	Van der Pol et al. 2017 (58)	**Performance calculation not based on PIS**
Knox et al., 2002 (59)			Kellogg et al. 2004 (60)
Kriesel et al., 2016 (61)		**Paper not available/located**	Tayoun et al. 2015 (62)
Li et al.2014., (63)			
Lawton et al. 2013 (64)		Lanjouw et al., 2009 (65)	**Results not stratified by sample site**
Macmillan et al., 2003 (66)		Ostergaard et al., 1997 (67)	McKechnie et al., 2011 (68)
Macmillan et al., 2003(69)		Cheng et al., 2014 (70)	
Ostergaard & Moller, 1995 (71)			
Ostergaard et al. 1996 (72)		**Cervical & vaginal samples combined**	
Polaneczky et al., 1998 (73)			
Renton et al., 2006 (74)		Koumans et al., 2003 (75)	
Rompalo et al., 2001 (76)		Leslie et al., 2003 (77)	
Rumyantseva et al 2015 (78)			

Modified Quality Assessment for Diagnostic Accuracy Studies (QUADAS-2) tool for assessment of included study quality

A modified tool, adapted from QUADAS-2 (79) was used to evaluate the following aspects of the included studies:

- **Risk of bias in patient selection:** Were patients selected at random (or in a consecutive manner of patients attending the clinic)? Were risk factors for the outcomes used to select patients included?
- **Risk of bias in patient infection status (PIS) definition:** Was PIS clearly defined? Had vaginal swabs been included in the PIS definition?
- **Risk of bias in flow and timing:** Is there a lag between the different tests done which could affect the results of the test? Were the same tests conducted for all patients (for example, was discrepant analysis used for equivocal test results?)?
- **Applicability concerns in patient selection:** Did the patient and the setting match the research question?
- **Applicability concerns for PIS definition:** Is the definition of PIS different from the review question?

Table S2. QUADAS-2 assessment for each included study

	Risk of bias			Applicability concerns	
Reference	Patient selection	PIS definition	Flow and timing	Patient selection	PIS definition
Chernesky *et al.,* 2006 (80)	Unclear	High	High	Unclear	Low
Chernesky *et al.*, 2014 (81)	Unclear	High	Low	Low	Low
Cosentino *et al.,* 2003 (82)	Unclear	High	High	High	Low
Gaydos *et al.,* 2010 (83)	Unclear	High	Unclear	Low	Low
Hook *et al.,* 1997 (84)	Unclear	High	High	Low	Low
Le Roy *et al.*, 2012 (85)	High	High	High	Low	High
Schachter *et al.,* 2005 (86)	Unclear	Low	Low	Low	Low
Shipitsyna *et al.,* 2009 (87)	High	High	Unclear	High	High
Stary et al., 1998 (88)	High	High	High	High	High

Overall, the sampling of patients to studies was not well described (unclear risk of bias) or selected patients with risk factors for the outcome (high risk of bias). All but Schachter *et al.* had included vaginal samples in their PIS definition which we considered likely to introduce a high risk of bias for the performace estimate (as discussed in the manuscript).

For flow and timing, the majority of studies were judged to have high risk of bias as they had used discrepant analysis (additional tests performed on patients with equivocal results) in their analyses or they had a different testing for some of the patients. There were less applicability concerns as concerns about risk of bias. Applicability of patient selection was unclear if the patient selection was poorly defined and of high concern if the patient population was at increased risk for the outcome (reported symptoms or were contacts of infected partners or reported high risk behaviours). PIS definition was marked as high concern if it was poorly explained (Shipitsyna *et al.*) or the performace estimate was not directly reported (Stary and Le Roy).

Table S3. Summary of results from studies that estimated performace of vaginal specimens relative to PIS for chlamydia

Author and year	Number of women who had chlamydia test	Chlamydia infected as defined by PIS (prevalence)	Sample performace relative to PIS (95% CI) [numbers reported by studies]		
			Vaginal samples	Endocervical samples	Urine
Chernesky M. et al. (2006)	298	69<br>(23%)	(AC2 against PIS)<br>99% (92-100%) [68/69]<br>(PT against PIS)<br>65% (53-76%) [45/69]<br>(AMP against PIS)<br>65% (52-76%) [45/69]	(AC2 against PIS)<br>90% (80-96%) [62/69]<br>(PT against PIS)<br>64% (51-75%) [44/69]<br>(AMP against PIS)<br>59% (47-71%) [41/69]	(AC2 against PIS)<br>81% (70-90%) [56/69]<br>(PT against PIS)<br>67% (54-78%) [46/69]<br>(AMP against PIS)<br>57% (44-68%) [39/69]
Chernesky M. *et al.*, (2014)	575	53<br>(9%)	AC2 (Tigris)<br>98.1 (90.1-99.7%)<br>AC2 (Panther)<br>96.2 (87.3-99.0%)<br><br>Real*Time* CT/NG<br>98.0 (89.7-99.7%)<br><br>ProbeTec CT/GC Qx<br>90.6 (79.8-95.9%)<br><br>Cobas CT/NG<br>84.6 (72.5-92.0%)	n/a	AC2 (Tigris)<br>88.7 (77.4-94.7%)<br>AC2 (Panther)<br>88.0 (76.2-94.4%)<br><br>Real*Time* CT/NG<br>76.9 (63.9—86.3%)<br><br>ProbeTec CT/GC Qx<br>75.5 (62.4-85.1%)<br><br>Cobas CT/NG<br>81.1 (68.6-89.4%)
Cosentino L.A. et al. (2003)	455	37<br>(8.1%)	(SDA against PIS) – SDA available for all sites<br>92% (78-98%) [34/37]	(SDA against PIS)<br>92% 78-98%) [34/37]	n/a
Gaydos C.A. et al. (2010)	2014 women enrolled	(Overall prevalence 9%)	Clinician-collected<br>Estimate and range presented<br>(ABT against PIS)<br>Symptomatic : 93% (84–97%)<br>Asymptomatic: 87% (74–95%)<br><br>(GP AC2 against PIS)<br>Symptomatic: 93% (85–97%)<br>Asymptomatic: 85% (72–94%)	(ABT against PIS)<br>Estimate and range presented<br>Symptomatic: 88% (79–94%)<br>Asymptomatic: 81% (67–91%)<br><br>(GP AC2 against PIS)<br>Symptomatic: 91% (83-97%)<br>Asymptomatic: 79% (64–89%)	(ABT against PIS)<br>Estimate and range presented<br>Symptomatic: 93% (85–97%)<br>Asymptomatic: 96% (85–100%)<br><br>(GP AC2 against PIS)<br>Symptomatic: 94% (86–98%)<br>Asymptomatic: 94% (82–99%)

Le Roy C. et al. (2012)	193	19 included in this analysis. Prevalence in the study was 20/193 (10%).	Bio-Rad Dx CT/NG/MG<br>100 % (82-100%) [19/19]<br><br>Cobas<br>100% (82-100%) [19/19]	Not evaluated as only 21 individuals had EC swab taken (3 Ct positives)	Bio-Rad Dx CT/NG/MG<br>100% (82-100%) [19/19]<br><br>Cobas<br>89% (67-99%) [17/19]
Schachter J et al. (2005)	1451	180<br>(12%)	Clinician-collected<br>Aptima Combo: 97%<br>Aptima CT: 97%<br><br>Patient-collected<br>Aptima Combo: 97%<br>Aptima CT: 98%	n/a	n/a
Shipitsyna E. et al. (2009)	298 (with both EC and vagina)	36<br>(12%)	100% (85-100%) [36/36]	97% (85-100%) [35/36]	n/a
Stary A et al. (1998)	308	25<br>(8%)	(TMA against PIS)<br>92% (74-99%) [23/25]<br><br>(LCR against PIS)<br>92%(74-99%) [23/25]	(TMA against PIS)<br>88% (69-97%) [22/25]<br><br>(LCR against PIS)<br>92% (74-99%) [23/25]	(TMA against PIS)<br>76% (55-91%) [19/25]<br><br>(LCR against PIS)<br>96% (80-100%) [24/25]

Table S4. **Summary of results from studies that estimated performace of vaginal specimens relative to PIS for gonorrhoea**

Author and year	Number of women who had gonorrhoea test	Gonorrhoea infected as defined by PIS (prevalence)	Sample performace relative to PIS		
			**Vaginal**	**Endocervical**	**Urine**
Chernesky M. *et al.*, (2014)	575	11<br>(2%)	AC2 (Tigris): 90.9%<br>AC2 (Panther): 100%<br>RealTime CT/NG: 70.0%<br>ProbeTec CT/GC Qx:100%<br>Cobas CT/NG:63.6%	n/a	AC2 (Tigris): 72.7%<br>AC2 (Panther): 66.7%<br>RealTime CT/NG: 70.0%<br>ProbeTec CT/GC Qx: 80.0%<br>Cobas CT/NG: 70.0%
Cosentino L.A. et al. (2003)	455	39<br>(8.6%)	100% (91-100%) [39/39]	100% (91-100%) [39/39]	n/a
Gaydos C.A. et al. (2010)	2014 women enrolled	Overall prevalence 3.8%	Clinician-collected<br>Estimate and range presented (ABT (RealTime) against PIS)<br>Symptomatic: 97% (83–100%)<br>Asymptomatic: 96% (78–100%)<br><br>(GP AC2 against PIS)<br>Symptomatic: 94% (79–99%)<br>Asymptomatic: 96% (78–100%)	Estimate and range presented (ABT (RealTime) against PIS)<br>Symptomatic: 87% (70–96%)<br>Asymptomatic: 91% (72–99%)<br><br>(GP AC2 against PIS)<br>Symptomatic: 91% (75–98%)<br>Asymptomatic: 91% (71%–99%)	Estimate and range presented (ABT (RealTime) against PIS)<br>Symptomatic: 94% (79–99%)<br>Asymptomatic: 87% (66–97%)<br><br>(GP AC2 against PIS)<br>Symptomatic: 84% (67–95%)<br>Asymptomatic:83% (61-95%)
Hook, E et al. (1997)	309	52<br>(16.8%)	Patient-obtained<br>100% (93-100%) [52/52]	85% (72-93%) [44/52]	
Le Roy et al. (2012)	193	2 (1%)	Not evaluated.		
Schachter J et al. (2005)	1450	78<br>(5.4%)	Clinician-collected<br>Aptima Combo: 96%<br>Aptima GC: 96%<br><br>Patient-collected<br>Aptima Combo: 99%<br>Aptima GC: 96%		

References

1. Bakken IJ, Bratt H, Skjeldestad FE, Nordbø SA. 2005. [Detection of chlamydia trachomatis in urine, vulval and cervical swabs]. Tidsskr den Nor lægeforening Tidsskr Prakt Med ny række 125:1629–30.
2. Schoeman SA, Stewart CMW, Booth RA, Smith SD, Wilcox MH, Wilson JD. 2012. Assessment of best single sample for finding chlamydia in women with and without symptoms: a diagnostic test study. BMJ 345:e8013.
3. Jaton K, Bille J, Greub G. 2006. A novel real-time PCR to detect Chlamydia trachomatis in first-void urine or genital swabs. J Med Microbiol 55:1667–1674.
4. Andrews J. 2009. Self-administered vaginal swabs were accurate for screening for sexually transmitted infections in the emergency department. Evid Based Med 14:150.
5. Behets FM-TF, Andriamiadana J, Randrianasolo D, Rasamilalao D, Ratsimbazafy N, Dallabetta G, Cohen MS. 2002. Laboratory diagnosis of sexually transmitted infections in women with genital discharge in Madagascar: implications for primary care. Int J STD AIDS 13:606–611.
6. Schmitt M, Depuydt C, Stalpaert M, Pawlita M. 2014. Bead-based multiplex sexually transmitted infection profiling. J Infect 69:123–133.
7. Le Roy C, Papaxanthos A, Liesenfeld O, Mehats V, Clerc M, Bébéar C, de Barbeyrac B. 2013. Swabs (dry or collected in universal transport medium) and semen can be used for the detection of Chlamydia trachomatis using the cobas 4800 system. J Med Microbiol 62:217–22.
8. Berwald N, Cheng S, Augenbraun M, Abu-Lawi K, Lucchesi M, Zehtabchi S. 2009. Self-administered vaginal swabs are a feasible alternative to physician-assisted cervical swabs for sexually transmitted infection screening in the emergency department. Acad Emerg Med 16:360–3.
9. Shrier L, Dean D, Klein E, Harter K, Rice P. 2004. Limitations of Screening Tests for the Detection of Chlamydia trachomatis in Asymptomatic Adolescent and Young Adult Women. Obs Gynecol Surv 59:513–515.
10. Shalepo K, Savicheva A, Shipitsyna E, Unemo M, Domeika M. 2006. Diagnosis of Chlamydia trachomatis in Russia - In-house PCR assays may be effective but overall optimization and quality assurance are urgently needed. Apmis 114:500–507.
11. Black CM, Driebe EM, Howard L a, Fajman NN, Sawyer MK, Girardet RG, Sautter RL, Greenwald E, Beck-Sague CM, Unger ER, Igietseme JU, Hammerschlag MR, MD P, Igietseme JU, Hammerschlag MR, C.M. B-SB, E.M. D, L.A. H, N.N. F, M.K. S, R.G. G, R.L. S, E. G, E.R. U, J.U. I, M.R. H. 2009. Multicenter study of nucleic acid amplification tests for detection of Chlamydia trachomatis and Neisseria gonorrhoeae in children being evaluated for sexual abuse. Pediatr Infect Dis J 28:608–613.
12. Skidmore S, Horner P, Herring A, Sell J, Paul I, Thomas J, Caul EO, Egger M, McCarthy E, Sanford E, Salisbury C, Macleod J, Sterne J, Low L, Group CSS (ClaSS) P. 2006. Vulvovaginal-swab or first-catch urine specimen to detect Chlamydia trachomatis in women in a community setting?. J Clin Microbiol 44:4389–4394.
13. Schachter J, McCormack WM, Chernesky MA, Martin DH, Van Der Pol B, Rice PA, Hook EW 3rd, Stamm WE, Quinn TC, Chow JM. 2003. Vaginal swabs are appropriate specimens for diagnosis of genital tract infection with Chlamydia trachomatis. J Clin Microbiol 41:3784–3789.
14. Bachmann LLH, Desmond RA, Stephens J, Hughes A, Hook EW, Stephens R, Hughes A, Hook III EW. 2002. Duration of persistence of gonococcal DNA detected by ligase chain reaction in men and women following recommended therapy for uncomplicated gonorrhea. J Clin Microbiol 40:3596–3601.

15. Carder C, Robinson AJ, Broughton C, Stephenson JM, Ridgway GL. 1999. Evaluation of self-taken samples for the presence of genital Chlamydia trachomatis infection in women using the ligase chain reaction assay. Int J STD AIDS 10:776–779.

16. Stewart CMWCMW, Schoeman SA, Booth RA, Smith SDSDD, Wilcox MHMHH, Wilson JDJDD. 2012. Assessment of self taken swabs versus clinician taken swab cultures for diagnosing gonorrhoea in women: single centre, diagnostic accuracy study. BMJ 345:e8107.

17. Samra Z, Rosenberg S, Madar-Shapiro L. 2011. Direct simultaneous detection of 6 sexually transmitted pathogens from clinical specimens by multiplex polymerase chain reaction and auto-capillary electrophoresis. Diagn Microbiol Infect Dis 70:17–21.

18. Chandeying V, Skov S, Farrell D, Lamlertkittikul S, Tabrizi S, Jarumanokul R. 2002. GYNAECOLOGY A Comparison of First-Void Urine , Self-Administered Low Vaginal Swab , Self-Inserted Tampon , and Endocervical Swab Using PCR Tests for the Detection of Infection with Chlamydia Trachomatis in Commercial Sex Workers and Women Attending Gyneco 14:201–209.

19. Sturm PDJ, Connolly C, Khan N, Ebrahim S, Sturm a W. 2004. Vaginal tampons as specimen collection device for the molecular diagnosis of non-ulcerative sexually transmitted infections in antenatal clinic attendees. Int J STD AIDS 15:94–8.

20. Upton A, Wilson J, Bissessor L. 2013. Introduction of routine polymerase chain reaction testing for Neisseria gonorrhoeae in a community laboratory. Sex Health. ELEC, CSIRO (P.O. Box 1139, Collingwood VIC 3066, Australia), A. Upton, Labtests, 27-41 Carbine Road, Mt Wellington 1060, New Zealand. E-mail: arlo.upton@labtests.co.nz.

21. Cheng A, Kirby JE. 2014. Evaluation of the hologic gen-probe PANTHER, APTIMA combo 2 assay in a tertiary care teaching hospital. Am J Clin Pathol 141:397–403.

22. Tanaka M, Nakayama H, Yoshida H, Takahashi K, Nagafuji T, Hagiwara T, Kumazawa J. 1998. Detection of Chlamydia trachomatis in vaginal specimens from female commercial sex workers using a new improved enzyme immunoassay. Sex Transm Infect 74:435–438.

23. Vahidnia A, Costa S, Veenings S, Tuin H, van Loon L, Bliekendaal H. 2014. Comparative evaluation of Roche Aurora FLOW, Becton and Dickinson Viper system, and Dynex DS2 for detection of Chlamydia trachomatis, Neisseria gonorrhoeae, and Trichomonas vaginalis in various clinical specimens. Diagn Microbiol Infect Dis 80:191–192.

24. Dietrich W, Rath M, Stanek G, Apfalter P, Huber JC, Tempfer C. 2010. Multiple site sampling does not increase the sensitivity of Chlamydia trachomatis detection in infertility patients. Fertil Steril 93:68–71.

25. Tanaka M, Nakayama H, Sagiyama K, Haraoka M, Yoshida H, Hagiwara T, Akazawa K, Naito S. 2000. Evaluation of a new amplified enzyme immunoassay (EIA) for the detection of Chlamydia trachomatis in male urine , female endocervical swab , and patient obtained vaginal swab specimens. J Clin Pathol 53:350–354.

26. Yu B, An Y, Xu G, Shan H. 2016. Detection of *Chlamydia trachomatis* and *Neisseria gonorrhoea* e based on cross-priming amplification. Lett Appl Microbiol 62:399–403.

27. Domeika M, Drulyte O. 2000. Use of PCR for the detection of genital Chlamydia trachomatis infection on self-obtained mailed vaginal samples. Acta Obstet Gynecol Scand 79:570–575.

28. Taylor SN, Van Der Pol B, Lillis R, Hook EW 3rd, Lebar W, Davis T, Fuller D, Mena L, Fine P, Gaydos CA, Martin DH. 2011. Clinical evaluation of the BD ProbeTecTM Chlamydia trachomatis Qx amplified DNA assay on the BD ViperTM system with XTRTM technology. Sex Transm Dis 38:603–609.

29. Zauli DAG, De Menezes CLP, De Oliveira CL. 2013. Development and padronization of three multiplex PCRs for the diagnosis of chlamydia trachomatis, toxoplasma gondii, herpes simplex viruses 1 and 2, and cytomegalovirus. Mol Biotechnol 54:1004–1009.

30. Domeika M, Bassiri M, Butrimiene I, Venalis a, Ranceva J, Vasjanova V. 1999. Evaluation of vaginal introital sampling as an alternative approach for the detection of genital Chlamydia trachomatis infection in women. Acta Obstet Gynecol Scand 78:131–136.

31. Thomas BJ, Pierpoint T, Taylor-Robinson D, Renton M. 1998. Sensitivity of the ligase chain reaction assay for detecting Chlamydia trachomatis in vaginal swabs from women who are infected at other sites. Sex Transm Infect 74:140–1.

32. Carroll KC, Aldeen WE, Morrison M, Anderson R, Lee D, Mottice S. 1998. Evaluation of the Abbott LCx ligase chain reaction assay for detection of Chlamydia trachomatis and Neisseria gonorrhoeae in urine and genital swab specimens from a sexually transmitted disease clinic population. J Clin Microbiol 36:1630–1633.

33. Falk L, Coble B-I, Mjörnberg P, Fredlund H. 2010. Sampling for Chlamydia trachomatis infection - a comparison of vaginal, first-catch urine, combined vaginal and first-catch urine and endocervical sampling. Int J STD AIDS 21:283–287.

34. Thomas BJ, Pierpoint T, Taylor-Robinson D, Renton M. 2001. Qualitative and quantitative aspects of the ligase chain reaction assay for Chlamydia trachomatis in genital tract samples and urines. Int J STD AIDS 12:589–594.

35. Lee GI, Yoen JP, Kang JS, Hwang SY, Hong YM, Yang JH, Yoon HK. 2013. A comparison of oligonucleotide-based microarray and real-time PCR for the detection of sexually transmitted infections. Biochip J 7:68–74.

36. Fang J, Husman C, DeSilva L, Chang R, Peralta L. 2008. Evaluation of self-collected vaginal swab, first void urine, and endocervical swab specimens for the detection of Chlamydia trachomatis and Neisseria gonorrhoeae in adolescent females. J Pediatr Adolesc Gynecol 21:355–360.

37. Van Der Pol B, Taylor SN, Lebar W, Davis T, Fuller D, Mena L, Fine P, Gaydos C a., Martin DH, Hook EW. 2012. Clinical Evaluation of the BD ProbeTec™ Neisseria gonorrhoeae Qx Amplified DNA Assay on the BD Viper™ System With XTR™ Technology. Sex Transm Dis 39:147–153.

38. Nuñez-Forero L, Moyano-Ariza L, Gaitán-Duarte H, Ángel-Müller E, Ruiz-Parra A, González P, Rodríguez A, Tolosa JE. 2016. Diagnostic accuracy of rapid tests for sexually transmitted infections in symptomatic women. Sex Transm Infect 92:24–28.

39. Fernández G, Martró E, González V, Saludes V, Bascuñana E, Marcó C, Rivaya B, López E, Coll P, Matas L, Ausina V. 2016. Usefulness of a novel multiplex real-time PCR assay for the diagnosis of sexually-transmitted infections. Enferm Infecc Microbiol Clin 34:471–6.

40. Garrow SC, Smith DW, Harnett GB. 2002. The diagnosis of chlamydia, gonorrhoea, and trichomonas infections by self obtained low vaginal swabs, in remote northern Australian clinical practice. Sex Transm Infect 78:278–281.

41. Van Der Pol B, Taylor SN, Liesenfeld O, Williams JA, Hook EW. 2013. Vaginal swabs are the optimal specimen for detection of genital Chlamydia trachomatis or Neisseria gonorrhoeae using the Cobas 4800 CT/NG test. Sex Transm Dis 40:247–50.

42. Gaydos CA, Van Der Pol B, Jett-Goheen M, Barnes M, Quinn N, Clark C, Daniel GE, Dixon PB, Hook EW 3rd, Group CS. 2013. Performance of the Cepheid CT/NG Xpert Rapid PCR Test for Detection of Chlamydia trachomatis and Neisseria gonorrhoeae. J Clin Microbiol 51:1666–1672.

43. Van Der Pol B, Hook 3rd EW, Williams JA, Smith B, Taylor SN. 2015. Performance of the BD CTQx and GCQx Amplified Assays on the BD Viper LT Compared With the BD Viper XTR System. Sex Transm Dis 42:521–523.

44. CHERNESKY MA, JANG DA. 2006. APTIMA transcription-mediated amplification assays for Chlamydia trachomatis and Neisseria gonorrhoeae. Expert Rev Mol Diagn 6:519–525.

45. Gokral JS, Mania-Pramanik J, Meherji PK, Mali BN. Introital swab testing for Chlamydia trachomatis in a resource-poor setting: an Indian perspective. Int J Fertil Womens Med 50:140–3.

46. Van Der Pol B, Williams JA, Fuller DA, Taylor SN, Hook EW. 2017. Combined testing for chlamydia, gonorrhea, and trichomonas by use of the BD max CT/GC/TV assay with genitourinary specimen types. J Clin Microbiol 55:155–164.

47. Fong H, Christian CW. 2012. Evaluating Sexually Transmitted Infections in Sexually Abused Children: New Techniques to Identify Old Infections. Clin Pediatr Emerg Med 13:202–212.

48. Gaydos C, Crotchfelt K, Shah N, Tennant M, Quinn TC, Gaydos JC, McKee KT, Rompalo AM. 2002. Evaluation of dry and wet transported intravaginal swabs in detection of Chlamydia trachomatis and Neisseria gonorrhoeae infections in female soldiers by PCR. J Clin Microbiol 40:758–761.

49. Gaydos CA. 2014. Review of use of a new rapid real-time PCR, the Cepheid GeneXpert ® (Xpert) CT/NG assay, for *Chlamydia trachomatis* and *Neisseria gonorrhoeae* : results for patients while in a clinical setting. Expert Rev Mol Diagn 14:135–137.

50. Hjelm E, Hallén a., Domeika M. 2001. Cervical, urine and vaginal specimens for detection of Chlamydia trachomatis by ligase chain reaction in women: A comparison. Acta Derm Venereol 81:285–288.

51. van Dommelen L, Dukers-Muijrers N, Van Tiel F, Brouwers E, Hoebe C. 2011. Evaluation of one-sample testing of self-obtained vaginal swabs and first-catch urine samples separately and in combination for the detection of Chlamydia trachomatis by two amplified DNA assays in women visiting a sexually transmitted disease clinic. Clin Microbiol Infect 17:S754–S755.

52. Meyer T, T. M. 2012. [Diagnosis and treatment of Chlamydia trachomatis infections]. Hautarzt 63:16–23.

53. Hsieh Y-H, Howell MR, Gaydos JC, Mckee KT, Quinn TC, Gaydos C a, MD MsPH, Mckee KT, Quinn TC, Gaydos C a. 2003. Preference among female Army recruits for use of self-administrated vaginal swabs or urine to screen for Chlamydia trachomatis genital infections. Sex Transm Dis 30:769–773.

54. Wiesenfeld HC, Heine RP, Rideout a., Macio I, DiBiasi F, Sweet RL. 1996. The vaginal introitus: A novel site for Chlamydia trachomatis testing in women. Am J Obstet Gynecol 174:1542–1546.

55. Sautter RL, LeBar WD, Greenwald E. 2009. The Laboratory's Role in Evaluating Sexually Transmitted Diseases as a Result of Sexual Abuse. Clin Microbiol Newsl 31:145–150.

56. Keane F, Bendall R, Saulsbury N, Haddon L. 2007. A comparison of self-taken vulvovaginal and cervical samples for the diagnosis of Chlamydia trachomatis infection by polymerase chain reaction. Int J STD AIDS 18:98–100.

57. Witkin SS, Inglis SR, Polaneczky M. 1996. Detection of Chlamydia trachomatis and Trichomonas vaginalis by polymerase chain reaction in introital specimens from pregnant women. Am J Obstet Gynecol 175:165–167.

58. Van Der Pol B. 2017. Profile of the triplex assay for detection of chlamydia, gonorrhea and trichomonas using the BD MAX^{TM} System. Expert Rev Mol Diagn 17:539–547.

59. Knox J, Tabrizi SN, Miller P, Petoumenos K, Law M, Chen S, Garland SM. 2002. Evaluation of self-collected samples in contrast to practitioner-collected samples for detection of Chlamydia trachomatis, Neisseria gonorrhoeae, and Trichomonas vaginalis by polymerase chain reaction among women living in remote areas. Sex Transm Dis 29:647–54.

60. Kellogg ND, Baillargeon J, Lukefahr JL, Lawless K, Menard SW. 2004. Comparison of nucleic acid amplification tests and culture techniques in the detection of Neisseria gonorrhoeae and Chlamydia trachomatis in victims of suspected child sexual abuse. J Pediatr Adolesc Gynecol 17:331–339.

61. Kriesel JD, Bhatia AS, Barrus C, Vaughn M, Gardner J, Crisp RJ. 2016. Multiplex PCR testing for nine different sexually transmitted infections. Int J STD AIDS 27:1275–1282.

62. Abou Tayoun AN, Burchard PR, Caliendo AM, Scherer A, Tsongalis GJ. 2015. A multiplex PCR assay for the simultaneous detection of Chlamydia trachomatis, Neisseria gonorrhoeae, and Trichomonas vaginalis. Exp Mol Pathol 98:214–218.

63. Li J, Jang D, Gilchrist J, Smieja M, Ewert R, MacRitchie C, Chernesky M. 2014. Comparison of flocked and Aptima swabs and two specimen transport media in the Aptima combo 2 assay. J Clin Microbiol 52:3808–3809.

64. Lawton B, Bromhead C. 2013. Nucleic acid amplification tests of self-taken vulvovaginal swabs are more sensitive than clinician taken endocervical culture for gonorrhoea. Evid Based Med 18:e46.

65. Lanjouw E, van Daele PLA, Raes MPE, van der Meijden WI. 2009. Consecutively Acquired Sexually Transmitted Infections Mimicking Crohn's Disease. Am J Gastroenterol 104:532–533.

66. Macmillan S, McKenzie H, Templeton A, Strandell A. 2003. Screening for chlamydial infection by DNA testing in urine and vulval swabs was highly sensitive and more acceptable than endocervical swabs. Evidence-based Obstet Gynecol 5:120–121.

67. Ostergaard LJ, Moller JK, Andersen B, Olesen F. 1997. Self-collected and mailed samples for diagnosis of urogenital Chlamydia trachomatis infection in women [Danish]. Ugeskr Laeger 159:5089–92.

68. McKechnie ML, Hillman RJ, Jones R, Lowe PC, Couldwell DL, Davies SC, King F, Kong F, Gilbert GL. 2011. The prevalence of urogenital micro-organisms detected by a multiplex PCR-reverse line blot assay in women attending three sexual health clinics in Sydney, Australia. J Med Microbiol 60:1010–1016.

69. Macmillan S, McKenzie H, Templeton A. 2003. Parallel observation of four methods for screening women under 25 years of age for genital infection with Chlamydia trachomatis. Eur J Obstet Gynecol Reprod Biol 107:68–73.

70. Cheng P, Tang J, Cheng H, Liu X, Wang Y. 2014. PCR methods for simultaneous detection of Neisseria agonorrhoeae, Chlamydiatrachomatis and Ureaplasma parvum. Chinese J Biol 27:109–114.

71. Ostergaard L, Møller JK. 1995. Use of PCR and direct immunofluorescence microscopy for confirmation of results obtained by Syva MicroTrak Chlamydia enzyme immunoassay. J Clin Microbiol 33:2620–3.

72. Ostergaard L, Møller JK, Andersen B, Olesen F. 1996. Diagnosis of urogenital Chlamydia trachomatis infection in women based on mailed samples obtained at home: multipractice comparative study. BMJ 313:1186–9.

73. Polaneczky M, Quigley C, Pollock L, Dulko D, Witkin SS. 1998. Use of self-collected

vaginal specimens for detection of Chlamydia trachomatis infection. Obstet Gynecol 91:375–8.

74. Renton A, Thomas BM, Gill S, Lowndes C, Taylor-Robinson D, Patterson K. 2006. Chlamydia trachomatis in cervical and vaginal swabs and urine specimens from women undergoing termination of pregnancy. Int J STD AIDS 17:443–447.

75. Koumans EH, Black CM, Markowitz LE, Unger ER, Pierce A, Sawyer MK, Papp JR. 2003. Comparison of Methods for Detection of Chlamydia trachomatis and Neisseria gonorrhoeae Using Commercially Available Nucleic Acid Amplification Tests and a Liquid Pap Smear Medium. Society 41:1507–1511.

76. Rompalo AM, Gaydos CA, Shah N, Tennant M, Crotchfelt KA, Madico G, Quinn TC, Daniel R, Shah K V, Gaydos JC, McKee KT. 2001. Evaluation of use of a single intravaginal swab to detect multiple sexually transmitted infections in active-duty military women. Clin Infect Dis 33:1455–1461.

77. Leslie DE, Azzato F, Ryan N, Fyfe J. 2003. An assessment of the Roche Amplicor ® gonorrhoeae multiplex PCR assay in routine diagnostic use on a variety of specimen types. Commun Dis Intell Q Rep 27:373–9.

78. Rumyantseva T, Golparian D, Nilsson CS, Johansson E, Falk M, Fredlund H, Van Dam A, Guschin A, Unemo M. 2015. Evaluation of the new AmpliSens multiplex real-time PCR assay for simultaneous detection of Neisseria gonorrhoeae, Chlamydia trachomatis, Mycoplasma genitalium, and Trichomonas vaginalis. Apmis 123:879–886.

79. Whiting PF, Rutjes AWS, Westwood ME, Mallett S, Deeks JJ, Reitsma JB, Leeflang MMG, Sterne JAC, Bossuyt PMM. 2011. QUADAS-2: a revised tool for the quality assessment of diagnostic accuracy studies. Ann Intern Med 155:529–36.

80. Chernesky MA, Jang D, Luinstra K, Chong S, Smieja M, Cai W, Hayhoe B, Portillo E, Main C, Ewert R, Macritchie C, Main C, Ewert R, Main MacRitchie C, Ewert R. 2006. High analytical sensitivity and low rates of inhibition may contribute to detection of Chlamydia trachomatis in significantly more women by the APTIMA Combo 2 assay. J Clin Microbiol 44:400–405.

81. Chernesky M, Jang D, Gilchrist J, Hatchette T, Poirier A, Flandin JF, Smieja M, Ratnam S. 2014. Head to Head Comparison of Second Generation Nucleic Acid Amplification Tests for Chlamydia trachomatis and Neisseria gonorrhoeae on Female Urines and Self-Collected Vaginal Swabs. J Clin Microbiol 52:2305–10.

82. Cosentino LA, Landers D V, Hillier SL. 2003. Detection of Chlamydia trachomatis and Neisseria gonorrhoeae by strand displacement amplification and relevance of the amplification control for use with vaginal swab specimens. J Clin Microbiol 41:3592–6.

83. Gaydos CA, Cartwright CP, Colaninno P, Welsch J, Holden J, Ho SY, Webb EM, Anderson C, Bertuzis R, Zhang L, Miller T, Leckie G, Abravaya K, Robinson J. 2010. Performance of the Abbott RealTime CT/NG for detection of Chlamydia trachomatis and Neisseria gonorrhoeae. J Clin Microbiol 48:3236–43.

84. Hook EW 3rd, Ching SF, Stephens J, Hardy KF, Smith KR, Lee HH. 1997. Diagnosis of Neisseria gonorrhoeae infections in women by using the ligase chain reaction on patient-obtained vaginal swabs. J Clin Microbiol 35:2129–2132.

85. Le Roy C, Le Hen I, Clerc M, Arfel V, Normandin F, Bebear C, de Barbeyrac B. 2012. The first performance report for the Bio-Rad Dx CT/NG/MG assay for simultaneous detection of Chlamydia trachomatis, Neisseria gonorrhoeae and Mycoplasma genitalium in urogenital samples. J Microbiol Methods 89:193–197.

86. Schachter J, Chernesky MA, Willis DE, Fine PM, Martin DH, Fuller D, Jordan JA, Janda W, Hook EW. 2005. Vaginal swabs are the specimens of choice when screening for Chlamydia trachomatis and Neisseria gonorrhoeae: results from a

multicenter evaluation of the APTIMA assays for both infections. Sex Transm Dis 32:725–8.

87. Shipitsyna E, Zolotoverkhaya E, Agne-Stadling I, Krysanova A, Savicheva A, Sokolovsky E, Domeika M, Unemo M. 2009. First evaluation of six nucleic acid amplification tests widely used in the diagnosis of Chlamydia trachomatis in Russia. J Eur Acad Dermatology Venereol 23:268–276.

88. Stary A, Schuh E, Kerschbaumer M, Götz B, Lee H. 1998. Performance of transcription-mediated amplification and ligase chain reaction assays for detection of chlamydial infection in urogenital samples obtained by invasive and noninvasive methods. J Clin Microbiol 36:2666–70.
