# Supplementary material for: Evaluation of the performance of nucleic acid amplification tests (NAATs) in detection of chlamydia and gonorrhoea infection in vaginal specimens relative to patient infection status: a systematic review
Source: BMJ Open. 2019 Jan 17;9(1):e022510. doi: 10.1136/bmjopen-2018-022510 (PMC6340625; doi:10.1136/bmjopen-2018-022510)
Supplement: Author's manuscript [file bmjopen-2018-022510.draft_revisions.pdf]

BMJ Open is committed to open peer review. As part of this commitment we make the peer review history of every article we publish publicly available.

When an article is published we post the peer reviewers' comments and the authors' responses online. We also post the versions of the paper that were used during peer review. These are the versions that the peer review comments apply to.

The versions of the paper that follow are the versions that were submitted during the peer review process. They are not the versions of record or the final published versions. They should not be cited or distributed as the published version of this manuscript.

BMJ Open is an open access journal and the full, final, typeset and author-corrected version of record of the manuscript is available on our site with no access controls, subscription charges or pay-per-view fees (http://bmjopen.bmj.com).

If you have any questions on BMJ Open's open peer review process please email info.bmjopen@bmj.com

BMJ Open

Evidence-based evaluation of the sensitivity of vaginal specimens in detecting chlamydia and gonorrhoea infection relative to patient infection status: a systematic review

Journal:	*BMJ Open*
Manuscript ID	bmjopen-2018-022510
Article Type:	Research
Date Submitted by the Author:	22-Feb-2018

Complete List of Authors:	Rönn, Minttu; Imperial College London, Department of Infectious Disease Epidemiology; Harvard T. H. Chan School of Public Health, Department of Global Health and Population<br>Mc Grath-Lone, Louise; Imperial College London, Department of Infectious Disease Epidemiology<br>Davies, Bethan; Imperial College London, Department of Infectious Disease Epidemiology<br>Wilson, Janet; Leeds General Infirmary,<br>Ward, Helen; Imperial College London, Department of Infectious Disease Epidemiology

Keywords:	chlamydia, gonorrhea, patient infection status, Molecular diagnostics < INFECTIOUS DISEASES

SCHOLARONE™
Manuscripts

Evidence-based evaluation of the sensitivity of vaginal specimens in detecting chlamydia and gonorrhoea infection relative to patient infection status: a systematic review

AUTHORS:

Minttu M. Rönn[1,2], Louise Mc Grath-Lone[1], Bethan Davies[1], Janet D. Wilson[3], Helen Ward[1]

1 Department of Infectious Disease Epidemiology, Imperial College London, London, UK
2. Department of Global Health and Population, Harvard T.H. Chan School of Public Health
3 Leeds Sexual Health, Leeds Teaching Hospitals NHS Trust, Leeds, UK

Corresponding Author:
Minttu M. Rönn
Imperial College London
St Mary's Campus
Norfolk Place
W2 1PG London

MMR: mronn@hsph.harvard.edu
LM: lmcgrathlone@gmail.com
BD: bethan.davies06@imperial.ac.uk
JDW: janet-d.wilson@nhs.net
HW: h.ward@imperial.ac.uk

Abstract word count: 263
Manuscript word count: 3140
References: 33
Tables: 1 (in the manuscript)
Figures: 3 (attached)
Supplemental material: 1 (attached)

Keywords: chlamydia, gonorrhea, diagnostics, patient infection status

For peer review only

For peer review only - http://bmjopen.bmj.com/site/about/guidelines.xhtml

ABSTRACT

OBJECTIVES: We aimed to assess the sensitivity of vaginal specimens in comparison to other urogenital specimens in their ability to detect chlamydia and gonorrhea infection in women.

SETTING: We conducted a systematic review using EMBASE and Ovid MEDLINE databases for eligible studies. We compared the sensitivity of specimens taken from the vagina to those from the cervix or urine in diagnosing chlamydia and gonorrhoea infection based on a conservative definition of patient infection status (PIS) among women.

PARTICIPANTS: We included studies that tested samples from the vagina and ≥1 other site (cervix and/or urine) with ≥2 NAATs for chlamydia and ≥2 NAATs or 1 NAAT and culture for gonorrhoea for each site.

PRIMARY AND SECONDARY OUTCOME MEASURES: We analysed the sensitivity of vaginal specimens compared to PIS.

RESULTS: Nine publications met the inclusion criteria (eight for chlamydia; six for gonorrhoea) and were narratively reviewed. Pooled summary estimates were not calculated due to the variable methodology and PIS definitions. Vaginal specimens performed similarly to cervical and urine specimens for chlamydia (range of sensitivity estimates: vaginal 65-100%, cervical 59-97%, urine 57-100%) and gonorrhoea (vaginal 64-100%, cervical 85-100%, urine 67-94%). Vaginal specimens were estimated to have a high sensitivity (>80%) for chlamydia and gonorrhoea infections in all but one study.

CONCLUSIONS: We found that for chlamydia and gonorrhoea, the sensitivity of vaginal specimens was similar to that of cervical and urine specimens relative to PIS. As vaginal samples have a higher acceptability and lower cost, this study can

For peer review only - http://bmjopen.bmj.com/site/about/guidelines.xhtml

support clinical testing guidelines by providing evidence that vaginal samples are a suitable alternative to traditional test sites.

ARTICLE SUMMARY

Strengths and limitations of this study

- The systematic review included studies which had used a conservative definition of patient infection status (PIS), which can improve estimates of sensitivity.
- The study included a broad time frame and the search did not have language restrictions, which increased the scope of the literature search.
- The lack of standardised definitions of PIS and variable methodologies used restricted our analyses.

For peer review only

INTRODUCTION

In the UK, practices for *Chlamydia trachomatis* (chlamydia) and *Neisseria gonorrhoeae* (gonorrhoea) testing are not currently standardised. Nucleic acid amplification tests (NAATs) are recommended for the diagnosis of chlamydia and gonorrhoea due to their high sensitivity and specificity (1–3), but a variety of urogenital specimens (i.e. urine, cervical and vaginal) are used for testing at clinic level. This is an issue because the sensitivity of NAATs to detect infection is known to vary by the type of specimen that is tested. For example, a systematic review by Cook *et al.* (2005) estimated that the overall sensitivity of NAATs for gonorrhoea was just 55.6% (95% CI: 36.3-74.9%) for urine samples compared to 94.2% (90.5-98.0%) for cervical samples, with a specificity of 98.7% (97.5-99.9%) and 99.2% (98.4-100%), respectively (4).

Recent studies have suggested that vaginal swabs may have a higher sensitivity for the detection of chlamydia and gonorrhoea than the traditionally-used urine and cervical samples (5–9). However, much of the evidence for the relative, superior performance of vaginal samples is based on studies that did not aim to directly compare NAAT performance between anatomical sites, but instead is inferred from studies that aimed to evaluate a novel NAAT (6, 9–14). An issue related to these studies is that the estimates may be overestimated due to the use of discrepant analysis. As there is no gold-standard diagnostic test for either chlamydia or gonorrhoea (i.e. one with 100% sensitivity and specificity), studies evaluating the performance of a novel NAAT have tended to compare its performance against presumably poorer performing existing NAATs. Given the assumption that the novel NAAT has a higher sensitivity, it would be reasonable to expect that it would yield a

For peer review only - http://bmjopen.bmj.com/site/about/guidelines.xhtml

positive result in some cases where the existing NAAT yields a negative result. However, this assumption creates a challenge in differentiating false positive results from true positive results for the novel NAAT. In this situation, a third test is often performed. If this third test is positive then the positive result from the novel NAAT is defined a “true positive” and if the third test is negative the result from the novel NAAT is defined a “false positive”. This approach is called discrepant analysis (DA). DA has been criticized because it only retests “false positives” and doesn’t seek to improve the classification of “false negatives” which potentially introduces a bias towards overestimating the performance of the novel NAAT (15).

To address some of the potential bias from DA, patient infection status (PIS) was developed to represent an “extended gold-standard” by measuring test performance based on information from multiple sources. There is no consensus definition for PIS, but in general, it involves performing ≥1 test(s) at >1 site and interpreting the results using an algorithm that describes the number of positive tests (by test type or site) needed to define a person as infected. Thus, PIS is determined with the results of multiple NAATs adjusting for potential problems with the NAATs as well as for the possibility that a person might be infected at one or more anatomical sites (16). PIS is not a perfect reference-standard, and it has received criticism for similar reasons as discrepant analysis whereby there is a conditional dependence in the estimation test performance and the PIS (17). The potential for overestimation for each site remains, but, nonetheless, PIS allows a meaningful comparison of specimens from different sites.

For peer review only

For peer review only - http://bmjopen.bmj.com/site/about/guidelines.xhtml

A further limitation of the current evidence base is that many studies that have concluded in favour of using vaginal samples over cervical or urine for the diagnosis of chlamydia and gonorrhoea have used just one NAAT per test site (6, 9–14). Therefore, the overall comparison of vaginal test performance compared to cervical and urine tests warrants further evaluation to inform evidence-based clinical testing guidelines. To this end, we performed a systematic review of the published literature. The primary aim of this review was to compare the sensitivity of specimens taken from the vagina to those from the cervix or urine in diagnosing chlamydia and gonorrhoea infection in women based on a PIS definition. Given the lack of calibrated gold-standard diagnostic tests and variation in the definition of PIS in the literature, we purposively used conservative selection criteria to identify studies that applied rigorous testing criteria in the process of defining PIS. We also examined the quality of the studies in light of the known biases in the PIS methodology to increase our ability to assess the evidence-base.

As clinical presentation and method of specimen collection may influence the sensitivity of NAATs, the secondary aims of this review were to evaluate whether there was a difference in the sensitivity of vaginal specimens (a) from asymptomatic versus symptomatic patients and (b) that were self-collected or clinician-collected.

METHODS

We performed a systematic review following PRISMA guidelines (18). Protocol for our review was not registered in advance. We included studies which (a) tested for urogenital chlamydia and/or gonorrhoea infection in (b) post-pubertal women (c) using a vaginal specimen (collected by any method) and (d) at least one other urine

For peer review only

or cervical specimen. We limited this review to studies that used a conservative approach for defining PIS whereby each specimen site had to be tested with (e) ≥2 NAATs for chlamydia and ≥2 NAATs or 1 NAAT plus culture for gonorrhoea (as culture is an accepted alternative to NAAT for gonorrhoea diagnosis (19)). Finally, studies had to (f) report the performance of a specimen site compared to the PIS, or give the numbers of patients infected and results from the tests so that this could be calculated.

NAATs were defined as commercially available or in-house tests involving DNA or RNA replication using polymerase chain reaction (PCR), transcription mediated amplification (TMA), ligase chain reaction (LCR) or strand displacement amplification (SDA). Studies that used pooled samples or novel point-of-care tests were excluded together with studies that used one NAAT on all samples and only confirmed positive or equivocal results with a second NAAT. No restrictions were set on year or language of publication. However, only peer-reviewed studies were included; conference abstracts/proceedings, notes, letters and book chapters were excluded.

The electronic databases EMBASE (1947 to 2013) and Ovid MEDLINE (1946 to 2013) were searched on 8 October 2013 by two authors, using OvidSP as the search platform (MMR and LM). The search was repeated on 3 October 2017 with date limits of “2013-current” (BD and MMR). The search strategy included keywords related to the infections, interventions, specimens of interest and outcomes. The full search strategy is presented in the supplement (File S1).

For peer review only - http://bmjopen.bmj.com/site/about/guidelines.xhtml

After duplicate references had been removed by OvidSP, two authors (MMR and LM, or BD and MMR for the latter search) independently selected studies by scanning titles and abstracts. A list of potentially eligible publications was obtained through consensus agreement and full texts were obtained online, through the British Library or directly from the authors. The full texts were then reviewed for inclusion and a third author was consulted if there was disagreement between the two reviewers. Where the language of the article was not English we consulted colleagues fluent in the relevant language. The reference lists of all studies meeting the inclusion criteria, as well as recent key papers and systematic reviews identified during the search process, were examined iteratively for additional references.

One author extracted information from the selected studies using a data extraction form in Microsoft Excel. The extracted data was checked against the studies by a second author. Two reviewers independently assessed the risk of bias in patient selection, PIS definition and flow and timing (of different tests and analysis, including here also discrepant analysis) for each included study using an adapted version of the quality assessment of diagnostic accuracy studies (QUADAS-2) tool (20). The purpose of this quality assessment was to consider the potential biases and variation within the individual studies that may account for heterogeneity across the studies. For each study, we calculated or reported the sensitivity of each NAAT at each test site. Sensitivity was defined as the “number of tests appearing to be a true positive at a site for a given NAAT / number of infected patients as determined by PIS”. We did this by extracting the available data and calculating confidence intervals using the exact binomial distribution. If the study did not present raw numbers, the summary estimate was extracted instead. These sensitivity measures were used to compare

For peer review only - http://bmjopen.bmj.com/site/about/guidelines.xhtml

the ability of each site to accurately predict PIS by describing the overall differences within and between sample sites for chlamydia and gonorrhoea. Calculations were done using Stata SE 11.2 and forest plots were constructed with Microsoft Excel.

Patient and Public Involvement

Vaginal specimens are an acceptable and sometimes a preferred way for chlamydia and gonorrhea testing among women.(21) In this study, we compare the evidence-base of sensitivity of different testing sites for detection of chlamydia and gonorrhea by conducting a systematic review of published primary research. We did not involve patient groups in the design or conduct of the study. The findings of the study can be used to standardize testing practices.

RESULTS

The search on 8 October 2013 identified 353 studies of which 233 remained after de-duplication by the search platform OvidSP. We excluded 168 studies based on their abstracts and screened the full text of the remaining 65 studies. We excluded 58 of these studies and the reasons for exclusion are presented in the supplement (File S1, Table S1). We identified one additional study through searching the reference lists (22) so that in total eight eligible studies were identified (23–29). The updated search on 3 October 2017 returned 214 studies, de-duplicated to 154 with one additional study identified through reference lists. Twenty studies were selected for full text review and one met the inclusion criteria (30). In total, nine eligible studies were included in this review: eight for chlamydia (23–30) and six for gonorrhoea (22, 24, 27, 29, 30). A flowchart describing study selection is presented in Figure 1 and a description of the included studies is presented in Table 1.

For peer review only

For peer review only - http://bmjopen.bmj.com/site/about/guidelines.xhtml

Figure 1. Flow chart of study selection. n_1 describes the first search in 2013, and n_2 the updated search in 2017.

For peer review only

For peer review only - http://bmjopen.bmj.com/site/about/guidelines.xhtml

228 **Table 1.**Characteristics of included studies

**Author and year (Reference) // Included infections**	**Review outcome the main focus of the study? // Aim of the study**	**Tests performed (acronym for the NAAT)**	**Specimens/sites (in women) // Comments**	**PIS definition // Comments on discrepant analysis**
**Chernesky M. et al. (2006)** (26)<br><br>CT	No<br><br>To compare detection thresholds and inhibitor and infection rates from different specimens	APTIMA Combo 2 assay (Gen-Probe Incorporated) (TMA AC2)<br><br>ProbeTec ET (SDA Probetec ET)<br><br>Amplicor (PCR AMP)	3 EC,3 CCVS and FCU divided into 3 aliquots<br><br>Each sample was tested as spiked (with added CT) or without added CT; seemingly all the tests were done to all the samples	≥1 site positive by ≥2 different tests or two specimens positive in a single test (≥2 out of 9 specimen-tests done); VS included in PIS<br><br>No discrepant analysis described
**Cherneskey M et al. (2014)** (30)<br><br>CT & NG	No<br><br>To compare the performance of four second-generation NAATs with FVU and VS	APTIMA COMBO 2<br><br>Real*Time* CT/NG<br><br>ProbeTec CT/GC Q^x<br><br>Cobas CT/NG	1 FCU and 4 SCVS<br><br>Each sample was tested as spiked (with added CT) or without added CT; all the tests were done to all the samples	≥2 of the 4 assays were positive for any specimen type; VS included in PIS<br><br>No discrepant analysis described
**Cosentino L.A. et al. (2003)** (27)<br><br>**CT & NG**	Yes<br><br>To compare vaginal swab specimens to endocervical swab specimens for the detection of CT and NG	PDprobeTec ET assay (SDA Probetec ET)<br><br>Amplicor Roche (PCR AMP)<br><br>Thayer-Martin medium and chocolate agar (for NG)<br><br>LCR for discrepant results	3 EC, 2 (presumably clinician-obtained) VS. One EC used for NG culture<br><br>CT was tested by PCR and SDA; NG (EC) was tested for by culture, SDA	Positive result by two different molecular tests for CT or for NG by culture or by two molecular tests; VS included in PIS<br><br>Discrepant analysis for indeterminate results by LCR
**Gaydos C.A. et al. (2010)**	No	Abbot RealTime CT/NG assay (PCR RealTime) – index	4 EC, 1 SCVS, 2 CCVS, 3 urine	≥1 positive result by both of the 2 reference NAATs, additionally for NG if culture

For peer review only - http://bmjopen.bmj.com/site/about/guidelines.xhtml
For peer review only

(24)<br><br>CT & NG	To compare the performance of the new tNAATs Abbott assays with the Gen-Probe Aptima Combo 2 assay	Gen-Probe APTIMA Combo2 Assay (TMA AC2)– reference<br><br>Becton Dickenson ProbeTec ET assay (SDA Probetec ET) – reference<br><br>NG culture	Only one NAAT performed on the SCVS (this test was not used to define PIS); results for this are not presented	positive the subject was defined as infected. Infection absent if ≥1 reference NAAT was negative for all sample types<br><br>Discrepant analysis: for CT retested equivocal results, for NG not done
**Hook, E et al. (1997)**<br>(22)<br><br>NG	Yes<br><br>To evaluate patient-obtained vaginal specimens tested with culture and LCR assays for NG compared to clinician-collected specimens	LCx Probe System, Abbot Laboratories (LCR)<br><br>Modified Thayer-Martin medium (culture)	3 SCVS, 3 EC<br>(one sample at each site not part of this study as processed for chlamydia)	Culture positive from either site;or LCR positive and culture negative with a positive confirmatory LCR; VS is included in PIS<br><br>Yes, alternative TMA with different target site to confirm samples where culture was negative and LCR was positive
**Le Roy C. et al. (2012)**<br>(23)<br><br>CT & NG[a]	No, data extracted based on their reporting.<br><br>Determine clinical performance of Bio-Rad CT/NG/MG assay for detection of chlamydia, gonorrhoea and mycoplasma genitalium	Bio-Rad Dx CT/NG/MG Assay (qPCR DxCT/NG/MG) – index test<br><br>Roche COBAS TaqMan CT (qPCR TaqMan) - reference<br><br>NG culture	Symptomatic: 2 SCVS, 2 EC and 2 FCU. Asymptomatic: 2 SCVS and FCU.<br><br>More tests done on symptomatic patients, but all samples seem to have been treated the same.	Study definition: At least 2 positive results from either of the two assays. We determined PIS based on test results for FCU and VS (which were available for all patients, see supplement material for further information); all infected patients had ≥2 positive tests and a positive test at both sites; VS is included in PIS<br><br>Discrepant analysis used for equivocal results
**Schachter J et al. (2005)**<br>(29)<br><br>**CT & NG**	Yes<br><br>To evaluate the performance of Gen-Probe's APTIMA assays on vaginal swabs for CT and NG	APTIMA CT Assay (TMA ACT)<br><br>APTIMA GC Assay (TMA AGC)<br><br>APTIMA COMBO 2 (TMA AC2)	1 FCU, 1 SCVS, 1 CCVS, 2 EC swabs<br><br>All samples tested with three TMAs (2 for CT and 2 for NG) FCU and 1x EC swab were also tested with BDProbeTec)	Infected if either BD or AC2 were positive on FCU or EC . VS not included in PIS<br><br>No discrepant analysis described

For peer review only - http://bmjopen.bmj.com/site/about/guidelines.xhtml

		BDProbeTEc ET System CT/GC Assays (SDA Probetec ET)		
**Shipitsyna E. et al. (2008)** (25)<br><br>CT	No<br><br>To evaluate the performance of five PCRs and a recently introduced nucleic acid sequence-based amplification (NASBA) assay	Different ‘in-house’ PCRs tested: cPCR-DT, rtPCR-DT, cPCR-Ly, cPCR-Ep, rtPCR-Ep, real-time NASBA assay<br><br>Reference methods:<br>Cobas Amplicor PCR, Roche Diagnoticss<br><br>LightMix 480HT PCR (TIM MOLBIOL GmbH)	Used the subsample who had 4 specimens collected each: 2 EC and 2 VS<br><br>All sample sites tested using reference NAAT and at least 3 other NAATs	Several (range) Russian PRCs used on the sample and the sample was considered true positive if a positive result by a Russian PCR was confirmed by the reference tests; VS included in PIS<br><br>Discrepant analysis
**Stary A et al. (1998)** (28)<br><br>**CT**	No<br><br>To compare TMA assay with LCR in detection of CT in genital tract with different specimen types	Gen-Probe Amplified CT Assay (TMA ACT)<br><br>LCR assay, Abbot (LCR)<br><br>McCoy cell culture<br><br>direct-fluorescent antibody assay (DFA) or alternative TMA for confirming discrepant results	3 EC, 3 CCVS, FCU<br><br>EC and VS tested with LCR, TMA and culture. FCU tested with LCR and TMA	Positive culture at any site or positive result by both NAATs in one site, or one positive NAAT confirmed with discrepant analysis; VS included in PIS.<br><br>Yes, used DFA or TMA with different target site to confirm samples where only one NAAT was positive

a. Too few infections with gonorrhoea for analysis

CT: *Chlamydia trachomatis*; NG: *Neisseria gonorrhoea*

For peer review only

For peer review only - http://bmjopen.bmj.com/site/about/guidelines.xhtml

CCVS: Clinician-collected vaginal specimens; SCVS: Self-collected vaginal specimens; EC: endocervical swabs; FCU: first catch urine; VS: vaginal specimen

Tests used: LCR (Ligase Chain Reaction); PCR AMP (Polymerase Chain Reaction Amplicor); PCR ‘In house’; PCR RealTi*me* (Polymerase Chain Reaction RealTi*me*); qPCR TaqMan (Quantitative Polymerase Chain Reaction TaqMan); qPCR DxCT/NG/MG (Quantitative Polymerase Chain Reaction DxCT/NG/MG); NASBA (Nucleic acid sequence-based amplification); SDA Probetec ET (Strand Displacement Amplification Probetec ET); TMA AC2 (Transcription Mediated Amplification Aptima Combo-2); TMA ACT (Transcription Mediated Amplification Aptima CT); TMA AGC (Transcription Mediated Amplification Aptima GC)

For peer review only

For peer review only - http://bmjopen.bmj.com/site/about/guidelines.xhtml

The factors considered in the modified QUADAS-2 assessment and the results are presented in the supplement (File S1, Table S2). All studies were rated as high or unclear risk for at least two of the five criteria related to risk of bias and applicability concerns; for example, risk for patient selection bias was either unclear (6/9) or high (3/9). Our inclusion criteria required there to be ≥2 NAATs for chlamydia and ≥2 NAATs or 1 NAAT plus culture for gonorrhoea performed at each anatomical site, but variation remained in the number and type of tests used in the PIS algorithms. For example, the definitions included "≥2 positive tests out of a number of tests performed (regardless of the site)", "≥2 different tests positive at the same site", "any test positive in ≥2 different sites" and "≥2 different tests positive" (described in Table 1). In all but one (29), the risk of bias in the definition of PIS was considered high because the algorithm used was unclearly defined and/or included vaginal specimens (as the component whose performance is being evaluated should be independent of the gold-standard it is measured against).

Seven out of nine studies (with the exception of Chernesky *et al.* 2006 (26) and Chernesky *et al.* 2014 (30)) had performed a different number of tests on a subsample of their study population where most of the studies (6/9) performed discrepant analysis for the tests with equivocal results. Due to the low number of studies identified, and their variable methods and definitions of PIS, we deemed pooling of the results to be inappropriate. Instead, we compared the ability of each site to predict PIS using forest plots of site-specific sensitivity from each study. Few studies included results that could be used to explore our secondary aims: three studies included patient obtained vaginal swabs (22, 29, 30) and just one reported results for asymptomatic and symptomatic patients separately (24).

For peer review only - http://bmjopen.bmj.com/site/about/guidelines.xhtml

Chlamydia

Of the eight studies that considered chlamydia test performance (23–30), two directly addressed the question in this review (27, 29) and the remainder aimed to test the performance of a novel NAAT in a clinical setting (23–26, 28, 30). All eight studies were performed in high-income countries. Where a broader description of the population sample was reported, the studies had recruited patients from sexual health or reproductive health clinics, youth centres or primary care settings. One study specified that the sample population was high-risk for acquisition of sexually transmitted infections (28).

The reported chlamydia prevalence in the included studies ranged from 8.1% to 23.2% based on varying definitions of PIS. The number of infected women according to PIS ranged from 19 to 69 (with one study giving a point estimate without the raw data (29)). The site-specific sensitivity estimates and their confidence intervals are presented in Figure 2 and the results are presented in table format in the supplement (File S1, Table S3). Across the studies vaginal specimens performed similarly to cervical and urine specimens (sensitivity range: vaginal 65-100%, cervical 59-97%, urine 57-100%). The lowest overall sensitivity estimates were reported by Chernesky *et al.* (2006) (26).

Figure 2. Sensitivity estimates for chlamydia by specimen site

Figure legend:

For peer review only - http://bmjopen.bmj.com/site/about/guidelines.xhtml

289 Each study is represented by a colour and where results are stratified by test, sampling or symptom status, this has been specified next to the author and year.

291 Tests used: LCR (Ligase Chain Reaction); PCR AMP (Polymerase Chain Reaction Ampicor); PCR 'In house'; PCR RealTi*me* (Polymerase Chain Reaction RealTi*me*); qPCR TaqMan (Quantitative Polymerase Chain Reaction TaqMan); qPCR DxCT/NG/MG (Quantitative Polymerase Chain Reaction DxCT/NG/MG); NASBA (Nucleic acid sequence-based amplification); SDA Probetec ET (Strand Displacement Amplification Probetec ET); TMA AC2 (Transcription Mediated Amplification Aptima Combo-2); TMA ACT (Transcription Mediated Amplification Aptima CT); TMA AGC (Transcription Mediated Amplification Aptima GC.

299 **Gonorrhoea**

300 We identified six studies that tested for gonorrhoea (22–24, 27, 29, 30). Of these, five had a sufficient number of infected women according to PIS for sensitivity to be calculated and were included in this analysis (22, 24, 27, 29, 30). The excluded study found two gonorrhoea cases in 193 tested individuals and these were not analysed further (23). Of the five remaining studies, three addressed the question in this review (22, 27, 29) whilst the remaining two compared the performance of a new assay (24, 30). As with chlamydia, all of the studies were performed in high-income countries.

308 The prevalence of gonorrhoea ranged from 2% to 16.8% in the five studies, based on PIS, as defined by the studies; the denominator used for sensitivity estimates varied from 11 to 52, with one study only reporting point estimates (29). Figure 3 presents the forest plot and the results are presented in table format in the supplement (File S1, Table S4). The sensitivity estimates at each site in the four studies were high: sensitivity range for vaginal samples was 63.6-100%, for cervical

For peer review only - http://bmjopen.bmj.com/site/about/guidelines.xhtml

samples 85-100% and for urine samples 66.7-94%. Three studies (22, 24, 27) included sensitivity estimates for cervical swabs and two studies had sensitivity estimates for urine (24, 30). The lowest overall sensitivity estimates were reported by Chernesky *et al.* (2014) (30).

Figure 3. Sensitivity estimates for gonorrhoea by specimen site

Figure legend:

Each study is represented by a colour and where results are stratified by test, sampling or symptom status, this has been specified next to the author and year.

Tests used: LCR (Ligase Chain Reaction); PCR AMP (Polymerase Chain Reaction Ampicor); PCR 'In house'; PCR RealT*ime* (Polymerase Chain Reaction RealT*ime*); qPCR TaqMan (Quantitative Polymerase Chain Reaction TaqMan); qPCR DxCT/NG/MG (Quantitative Polymerase Chain Reaction DxCT/NG/MG); NASBA (Nucleic acid sequence-based amplification); SDA Probetec ET (Strand Displacement Amplification Probetec ET); TMA AC2 (Transcription Mediated Amplification Aptima Combo-2); TMA ACT (Transcription Mediated Amplification Aptima CT); TMA AGC (Transcription Mediated Amplification Aptima GC.

DISCUSSION

We conducted a systematic review to evaluate the evidence for the relative performance of vaginal specimens in the detection of chlamydia and gonorrhoea in women using studies with conservative definitions of PIS. In the studies included in this review, we found similar sensitivities between urine, cervical and vaginal specimen types. This finding is contrary to other studies which have found that vaginal specimens have a higher sensitivity than cervical swabs (5, 7, 9, 31). This

For peer review only - http://bmjopen.bmj.com/site/about/guidelines.xhtml

difference may be due to the less conservative definition of PIS used in other studies, which may be more prone for overestimation.

This study followed PRISMA guidelines for systematic reviews and assessed the quality of the included studies using a modified version of QUADAS-2. The systematic searches were conducted by two authors and the results and discussion represent consensus opinion based on group discussion. There were no language or time restrictions and multiple databases were searched. Therefore, this review represents a robust and comprehensive summary of extant evidence on the comparative sensitivity of vaginal specimens in detecting chlamydia and gonorrhoea infection. A further strength of this study is that we excluded studies where only one test was performed at the vaginal site, even if ≥2 NAATs were used at all other sites (6, 10, 32, 33). By limiting the analysis to studies that performed ≥2 tests at multiple sites, we aimed to minimize the potential for overestimation of test sensitivity (16). A limitation of this study is that the majority of included studies did not aim to address the review question and the QUADAS-2 assessment found the studies were of variable quality and had methodological limitations and heterogeneity, particularly in PIS definition which was an important element of other analysis. As a result, some bias is likely to remain in the estimates reported in this review and the sensitivity estimates should not be directly compared across studies.

The impact of different definitions of PIS on sensitivity estimates is illustrated by two studies included in this review. Chernesky *et al.* (26, 30) presented lower sensitivity estimates for the SDA and PCR assays compared to the TMA assay, but Gaydos *et al.* (24) did not observe this phenomenon of superior sensitivity of TMA. In

For peer review only

Chernesky's study the TMA test identified more infected women than the SDA and PCR comparator assays. The authors suggest this could be due to (a) TMA targeting ribosomal RNA (of which there are many more copies in the infected cell) rather than DNA which is targeted by PCR and SDA, and (b) the TMA test producing fewer inhibited samples than the PCR and SDA tests. However, both of these explanations could be expected to also occur in Gaydos's study. Therefore, the differing definitions of PIS between the two studies may be responsible for the differing sensitivity estimates. For example, Chernesky *et al.* used three sampling sites and three NAATs and required ≥1 site positive by ≥2 tests or two sites positive with a single test while Gaydos *et al.* required a positive result from each of the reference NAATs. In Gaydos's study, two positive TMA tests would not have led to a positive PIS and the denominator for the sensitivity estimate would have been lower, leading to a higher estimate of sensitivity for the other assays. It may be that the subtle differences in the PIS definitions render the studies incomparable.

Overall, we found that vaginal specimens performed similarly to cervical and urine specimens for the diagnosis of both chlamydia and gonorrhoea in the identified studies. Based on the available evidence, and given the acceptability (21) and lower cost of vaginal samples (8), our study further supports that vaginal samples appear to provide an appropriate alternative to traditional cervical or urine testing methods. However, this review has also highlighted that there are a lack of studies that have attempted to explicitly answer this research question whilst taking into account the inherent biases in estimating diagnostic test sensitivity, particularly for NAATs. Further primary research with appropriate robust methodology for determining test performance is needed for a more authoritative comparison of the performance of

For peer review only - http://bmjopen.bmj.com/site/about/guidelines.xhtml

different urogenital sites. We suggest that research should be directed towards applying more sophisticated statistical methods for comparing test performance such as the latent class model described by Hagdu *et al.* (17), which uses a weighted statistical model acknowledging the conditional dependence of the tests.

PATIENT CONSENT

Not required

DATA SHARING STATEMENT

All data presented in this study is available in the manuscript or in the supplementary material.

FUNDING

MMR received funding from the Wellcome Trust (G090285/Z/09/Z) during the first review. BD has been funded by an MRC Population Health Scientist Fellowship (G0902120). HW, BD and LM were supported in part by the National Institute for Health Research (NIHR) Biomedical Research Centre based at Imperial College Healthcare NHS Trust and Imperial College London. JW has received research funding in the form of extra diagnostic reagents and equipment for one study from Hologic/Gen-Probe and has received one lecture fee from BD Diagnostics. HW and JW have received funding from the NIHR Research for Patient Benefit (RfPB) Programme. The funders had no role in study design, data collection and analysis, decision to publish, or preparation of the manuscript.

CONFLICT OF INTEREST

For peer review only

For peer review only - http://bmjopen.bmj.com/site/about/guidelines.xhtml

Authors declare no conflicts of interest.

AUTHOR CONTRIBUTIONS

HW and JW conceived the study idea. MMR and LM conducted the first review and BD and MMR conducted the update of the review with discrepancies solved by all three. MMR drafted the manuscript, which was subsequently edited by LM, BD, HW and JW. MMR prepared the tables and figures presented in the manuscript. All authors have read and accepted the final manuscript.

ACKNOWLEDGMENTS

The authors would like to thank Dr Mikaela Smit and Dr Annick Borquez for their help in evaluating and translating articles not available in English.

PRESENTATION AT A MEETING

Poster presented at 3rd Joint Conference of the British HIV Association, BHIVA with the British Association for Sexual Health and HIV, BASHH. Liverpool United Kingdom. Abstract: HIV Medicine 15 (pp 109-110), 2014.

For peer review only

For peer review only - http://bmjopen.bmj.com/site/about/guidelines.xhtml

REFERENCES

1. British Association for Sexual Health and HIV. 2015. Chlamydia trachomatis UK Testing Guidelines 1–17.

2. Bignell C, Fitzgerald M. 2011. UK national guideline for the management of gonorrhoea in adults, 2011. Int J STD AIDS 22:541–7.

3. Papp J, Schachter J, Gaydos C, Van Der Pol B. 2014. Recommendations for the laboratory-based detection of Chlamydia trachomatis and Neisseria gonorrhoeae--2014. MMWR Recomm Rep 63:1–19.

4. Cook RL, Hutchison SL, Østergaard L, Braithwaite RS, Ness RB. 2005. Systematic review: noninvasive testing for Chlamydia trachomatis and Neisseria gonorrhoeae. Ann Intern Med 142:914–25.

5. Schoeman SA, Stewart CMW, Booth RA, Smith SD, Wilcox MH, Wilson JD. 2012. Assessment of best single sample for finding chlamydia in women with and without symptoms: a diagnostic test study. BMJ 345:e8013.

6. Stewart CMWCMW, Schoeman SA, Booth RA, Smith SDSDD, Wilcox MHMHH, Wilson JDJDD. 2012. Assessment of self taken swabs versus clinician taken swab cultures for diagnosing gonorrhoea in women: single centre, diagnostic accuracy study. BMJ 345:e8107.

7. Shafer M, Moncada J, Boyer CB, Betsinger K, Flinn SD, Schachter J. 2003. Comparing first-void urine specimens, self-collected vaginal swabs, and endocervical specimens to detect Chlamydia trachomatis and Neisseria gonorrhoeae by a nucleic acid amplification test. J Clin Microbiol 41:4395–9.

8. Blake DR, Maldeis N, Barnes MR, Hardick A, Quinn TC, Gaydos CA. 2008. Cost-effectiveness of screening strategies for Chlamydia trachomatis using cervical swabs, urine, and self-obtained vaginal swabs in a sexually transmitted disease clinic setting. Sex Transm Dis 35:649–55.

9. Taylor SN, Van Der Pol B, Lillis R, Hook EW 3rd, Lebar W, Davis T, Fuller D, Mena L, Fine P, Gaydos CA, Martin DH. 2011. Clinical evaluation of the BD ProbeTecTM Chlamydia trachomatis Qx amplified DNA assay on the BD ViperTM system with XTRTM technology. Sex Transm Dis 38:603–609.

10. Van Der Pol B, Taylor SN, Liesenfeld O, Williams JA, Hook EW. 2013. Vaginal swabs are the optimal specimen for detection of genital Chlamydia trachomatis or Neisseria gonorrhoeae using the Cobas 4800 CT/NG test. Sex Transm Dis 40:247–50.

11. Falk L, Coble B-I, Mjörnberg P, Fredlund H. 2010. Sampling for Chlamydia trachomatis infection - a comparison of vaginal, first-catch urine, combined vaginal and first-catch urine and endocervical sampling. Int J STD AIDS 21:283–287.

12. Gaydos CA, Van Der Pol BB, Jett-Goheen M, Barnes M, Quinn N, Clark C, Daniel GE, Dixon PB, Hook EW 3rd, Group CS, Davis T, Pancholi P, Kerndt P, Horner P, Marrazzo J, De Burgh-Thomas A, Castro J, Ferguson D, Meyer M, Philip S, Van Der Pol BB, Thompson M, Taylor S, Ronk D, Fine P, Bachman L, Mayne D, Dietz C, Toevs K, Gettings N, Gall S, Patel R, Ross J, Brooks L, Hazan L, Clarke P, Groom R, Ackerman R, Anduczyk E, Mathews A, Blank S, Harris L, Tidings A, Moore P, Alexander S, Spurrell T, Group CS. 2013. Performance of the Cepheid CT/NG Xpert Rapid PCR Test for Detection of Chlamydia trachomatis and Neisseria gonorrhoeae. J Clin Microbiol 51:1666–1672.

13. Rompalo AM, Gaydos CA, Shah N, Tennant M, Crotchfelt KA, Madico G, Quinn TC, Daniel R, Shah K V, Gaydos JC, McKee KT. 2001. Evaluation of use of a single intravaginal swab to detect multiple sexually transmitted infections in active-duty military women. Clin Infect Dis 33:1455–1461.

For peer review only - http://bmjopen.bmj.com/site/about/guidelines.xhtml

14. van Dommelen L, Dukers-Muijrers N, Van Tiel F, Brouwers E, Hoebe C. 2011. Evaluation of one-sample testing of self-obtained vaginal swabs and first-catch urine samples separately and in combination for the detection of Chlamydia trachomatis by two amplified DNA assays in women visiting a sexually transmitted disease clinic. Clin Microbiol Infect 17:S754–S755.

15. Hadgu A. 1999. Discrepant analysis: a biased and an unscientific method for estimating test sensitivity and specificity. J Clin Epidemiol 52:1231–7.

16. Martin DH, Nsuami M, Schachter J, Iii EWH, Ferrero D, Quinn TC, Gaydos C, Hook EW 3rd, Ferrero D, Quinn TC, Gaydos C. 2004. Use of multiple nucleic acid amplification tests to define the infected-patient “gold standard” in clinical trials of new diagnostic tests for Chlamydia trachomatis infections. Society 42:4749–4758.

17. Hadgu A, Dendukuri N, Wang L. 2012. Evaluation of screening tests for detecting Chlamydia trachomatis: bias associated with the patient-infected-status algorithm. Epidemiology 23:72–82.

18. Moher D, Liberati A, Tetzlaff J, Altman DG, Group P. 2009. Preferred reporting items for systematic reviews and meta-analyses: the PRISMA statement. BMJ 339:b2535.

19. Bignell C, Unemo M. 2013. 2012 European guideline on the diagnosis and treatment of gonorrhoea in adults. Int J STD AIDS 24:85–92.

20. Whiting PF, Rutjes AWS, Westwood ME, Mallett S, Deeks JJ, Reitsma JB, Leeflang MMG, Sterne JAC, Bossuyt PMM. 2011. QUADAS-2: a revised tool for the quality assessment of diagnostic accuracy studies. Ann Intern Med 155:529–36.

21. Chernesky MA, Hook EW, Martin DH, Lane J, Johnson R, Jordan JA, Fuller D, Willis DE, Fine PM, Janda WM, Schachter J. 2005. Women find it easy and prefer to collect their own vaginal swabs to diagnose Chlamydia trachomatis or Neisseria gonorrhoeae infections. Sex Transm Dis 32:729–33.

22. Hook EW 3rd, Ching SF, Stephens J, Hardy KF, Smith KR, Lee HH. 1997. Diagnosis of Neisseria gonorrhoeae infections in women by using the ligase chain reaction on patient-obtained vaginal swabs. J Clin Microbiol 35:2129–2132.

23. Le Roy C, Le Hen I, Clerc M, Arfel V, Normandin F, Bebear C, de Barbeyrac B, Bébéar C, de Barbeyrac B. 2012. The first performance report for the Bio-Rad Dx CT/NG/MG assay for simultaneous detection of Chlamydia trachomatis, Neisseria gonorrhoeae and Mycoplasma genitalium in urogenital samples. J Microbiol Methods 89:193–197.

24. Gaydos CA, Cartwright CP, Colaninno P, Welsch J, Holden J, Ho SY, Webb EM, Anderson C, Bertuzis R, Zhang L, Miller T, Leckie G, Abravaya K, Robinson J. 2010. Performance of the Abbott RealTime CT/NG for detection of Chlamydia trachomatis and Neisseria gonorrhoeae. J Clin Microbiol 48:3236–43.

25. Shipitsyna E, Zolotoverkhaya E, Agne-Stadling I, Krysanova A, Savicheva A, Sokolovsky E, Domeika M, Unemo M. 2009. First evaluation of six nucleic acid amplification tests widely used in the diagnosis of Chlamydia trachomatis in Russia. J Eur Acad Dermatology Venereol 23:268–276.

26. Chernesky MA, Jang D, Luinstra K, Chong S, Smieja M, Cai W, Hayhoe B, Portillo E, Main C, Ewert R, Macritchie C, Main C, Ewert R, Main MacRitchie C, Ewert R. 2006. High analytical sensitivity and low rates of inhibition may contribute to detection of Chlamydia trachomatis in significantly more women by the APTIMA Combo 2 assay. J Clin Microbiol 44:400–405.

27. Cosentino LA, Landers D V, Hillier SL. 2003. Detection of Chlamydia trachomatis and Neisseria gonorrhoeae by strand displacement amplification and relevance of the amplification control for use with vaginal swab specimens. J Clin Microbiol 41:3592–6.

28. Stary A, Schuh E, Kerschbaumer M, Götz B, Lee H. 1998. Performance of transcription-

For peer review only - http://bmjopen.bmj.com/site/about/guidelines.xhtml

mediated amplification and ligase chain reaction assays for detection of chlamydial infection in urogenital samples obtained by invasive and noninvasive methods. J Clin Microbiol 36:2666–70.

29. Schachter J, Chernesky MA, Willis DE, Fine PM, Martin DH, Fuller D, Jordan JA, Janda W, Hook EW. 2005. Vaginal swabs are the specimens of choice when screening for Chlamydia trachomatis and Neisseria gonorrhoeae: results from a multicenter evaluation of the APTIMA assays for both infections. Sex Transm Dis 32:725–8.

30. Chernesky M, Jang D, Gilchrist J, Hatchette T, Poirier A, Flandin JF, Smieja M, Ratnam S. 2014. Head to Head Comparison of Second Generation Nucleic Acid Amplification Tests for Chlamydia trachomatis and Neisseria gonorrhoeae on Female Urines and Self-Collected Vaginal Swabs. J Clin Microbiol 52:2305–10.

31. Fang J, Husman C, DeSilva L, Chang R, Peralta L. 2008. Evaluation of self-collected vaginal swab, first void urine, and endocervical swab specimens for the detection of Chlamydia trachomatis and Neisseria gonorrhoeae in adolescent females. J Pediatr Adolesc Gynecol 21:355–360.

32. Koumans EH, Black CM, Markowitz LE, Unger ER, Pierce A, Sawyer MK, Papp JR. 2003. Comparison of Methods for Detection of Chlamydia trachomatis and Neisseria gonorrhoeae Using Commercially Available Nucleic Acid Amplification Tests and a Liquid Pap Smear Medium. Society 41:1507–1511.

33. Van Der Pol B, Williams JA, Fuller DA, Taylor SN, Hook EW. 2017. Combined testing for chlamydia, gonorrhea, and trichomonas by use of the BD max CT/GC/TV assay with genitourinary specimen types. J Clin Microbiol 55:155–164.

For peer review only

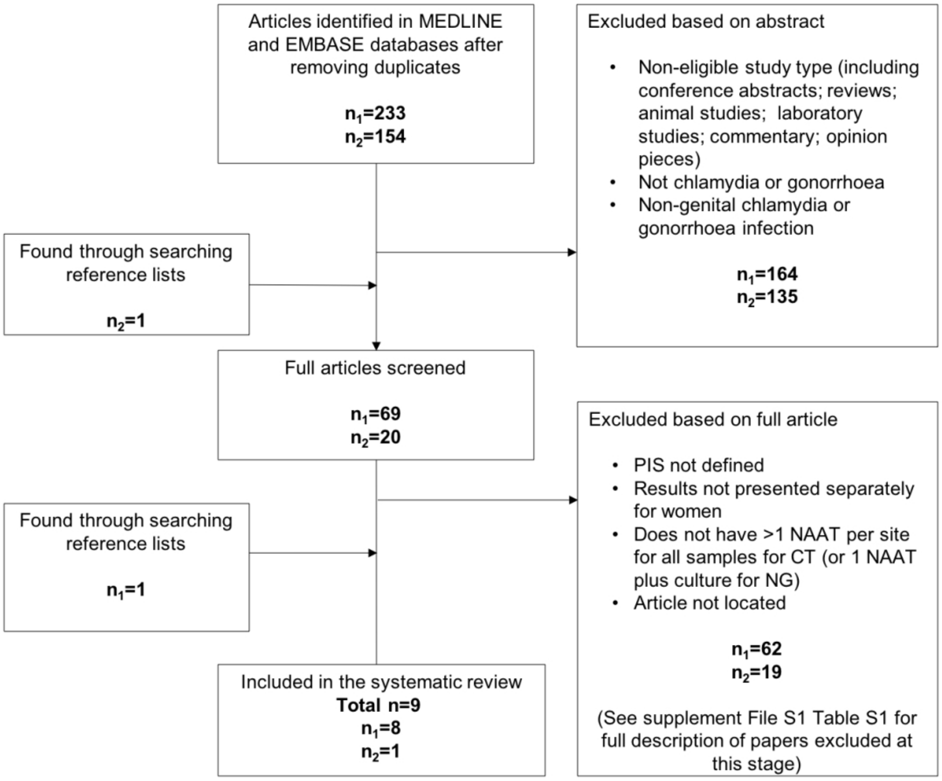

Figure 1. Flow chart of study selection. n1 describes the first search in 2013, and n2 the updated search in 2017.

239x189mm (150 x 150 DPI)

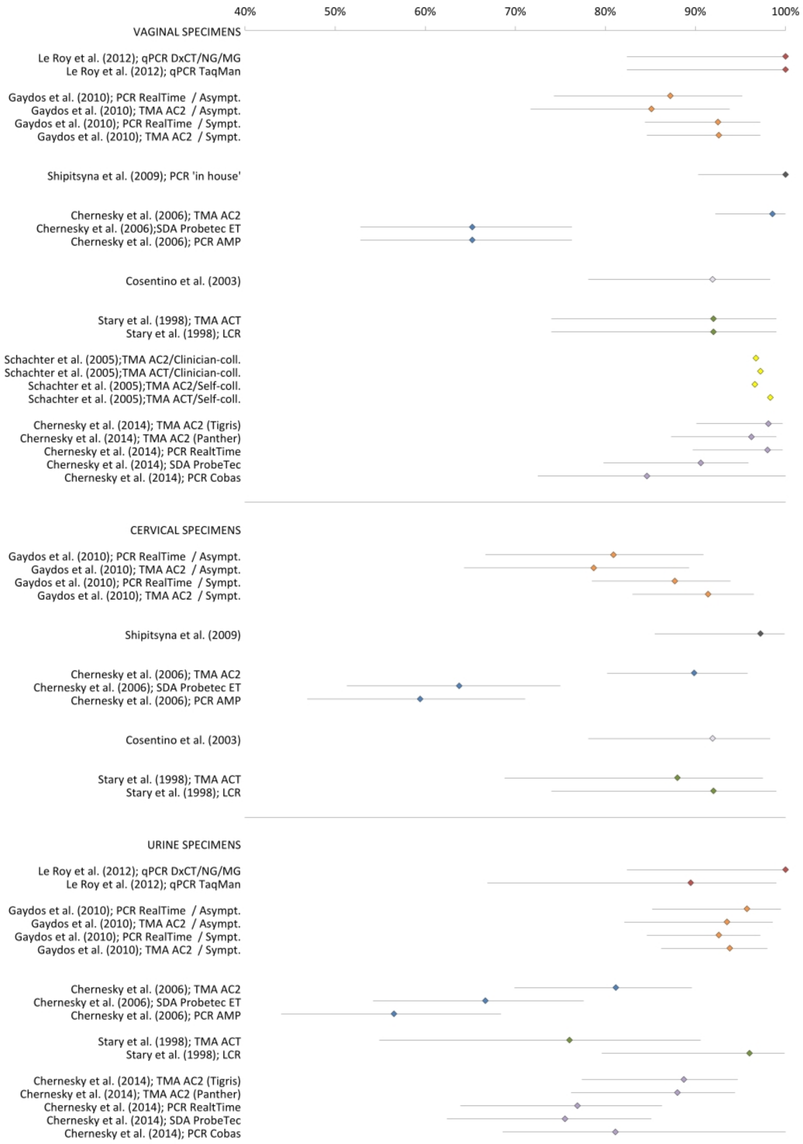

Figure 2. Sensitivity estimates for chlamydia by specimen site

708x945mm (150 x 150 DPI)

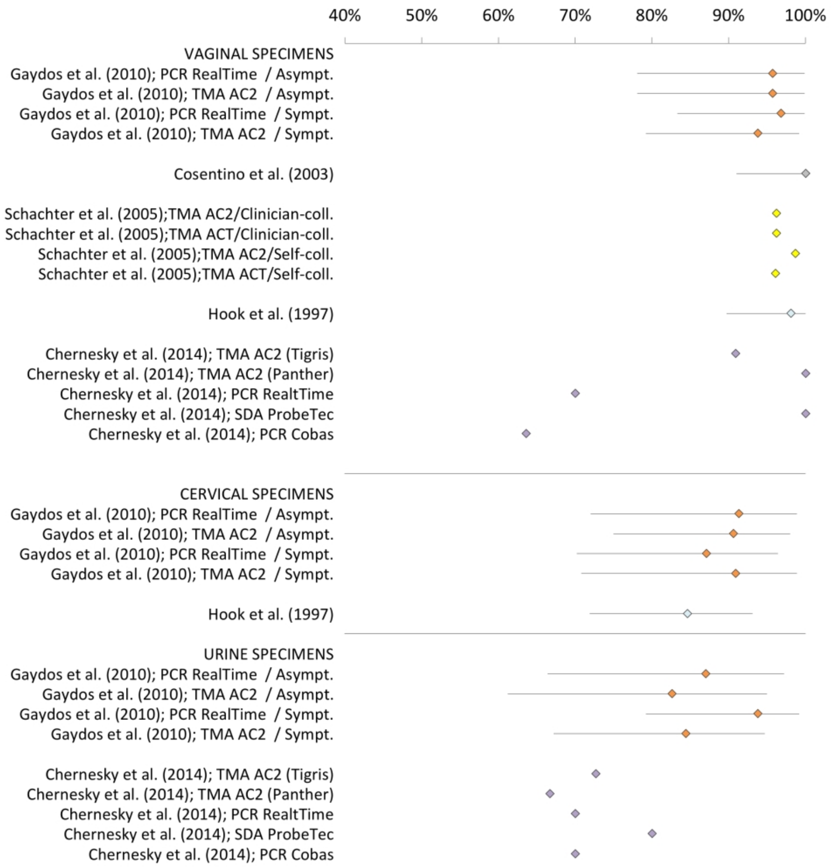

Figure 3. Sensitivity estimates for gonorrhoea by specimen site

527x478mm (150 x 150 DPI)

Supplemental File for

Evidence-based evaluation of the sensitivity of vaginal specimens in detecting chlamydia and gonorrhoea infection relative to patient infection status: a systematic review

Minttu M. Rönn, Louise Mc Grath-Lone, Bethan Davies, Janet Wilson, Helen Ward

For peer review only

Search strategy

The following Boolean searches were performed on OvidSP on 8 October 2013 and an update on 3 October 2017. We searched all fields (af) using Ovid MEDLINE (1947-present) and EMBASE (1946-present) databases. We combined the individual searches with AND in the following manner:

(chlamydia OR “chlamydia trachomatis”).af OR (gonorrh?ea OR “Neisseria gonorrhoeae”).af

AND

(vagina$.af OR vulvo-vagina$).af

AND

(urine OR endovervi$ OR cervi$).af

AND

(NAAT OR “nucleic acid amplification” OR (PCR OR “polymerase chain reaction”) OR (TMA OR “transcription mediated amplification OR “transcription-mediated amplification”) OR (SDA or “Strand displacement amplification”) OR (LCR or “ligase chain reaction”)).af

AND

(sensitivity OR specificity OR performance).af

AND

(diagnos$ OR screen$ OR test OR detect$).af

Table S1.Studies which were excluded based on the full article (n=58+n=19).

**Does not have >1 NAAT per site for all samples for CT (or 1 NAAT plus culture for NG)**		**Sensitivity calculation not based on PIS**	**Duplicate data**
Bakken et al., 2005 (1)	Schoeman et al., 2012 (2)	Jaton et al., 2006 (3)	Andrews, 2009 (4)
Behets et al., 2002 (5)	Schmitt et al, 2014 (6)	Le Roy et al., 2013 (7)	
Berwald et al., 2009(8)	Shrier et al., 2004 (9)	Shalepo et al., 2006 (10)	**Study population all infected**
Black et al., 2009 (11)	Skidmore et al., 2006 (12)	Schachter et al., 2003 (13)	Bachmann et al., 2002 (14)
Carder et al., 1999 (15)	Stewart et al., 2012 (16)	Samra et al., 2011 (17)	
Chandeying et al., 2004 (18)	Sturm et al., 2004 (19)	Upton et al., 2013 (20)	
Cheng et al. 2014 (21)	Tanaka et al., 1998 (22)	Vahidnia et al., 2014 (23)	
Dietrich et al., 2010 (24)	Tanaka et al., 2000 (25)	Yu et al., 2016(26)	**No vaginal samples**
Domeika & Drulyte, 2000 (27)	Taylor et al., 2011 (28)	Zauli et al., 2013 (29)	
Domeika et al.,1999 (30)	Thomas et al., 1998 (31)		Carroll et al. 1998 (32)
Falk et al., 2010 (33)	Thomas et al., 2001 (34)		Lee et al., 2013 (35)
Fang et al., 2008 (36)	Van Der Pol, 2012 (37)		Nunez-Forero et al. 2015 (38)
Fernandez et al., 2016 (39)	Van Der Pol et al., 2012 (28)		
Garrow et al., 2002 (40)	Van Der Pol et al., 2013 (41)	**No primary data reported**	
Gaydos et al., 2013 (42)	Van Der Pol et al., 2015 (43)	Chernesky & Jang, 2006 (44)	**Only vaginal samples**
Gokral et al., 2005 (45)	Van Der Pol et al., 2017 (46)	Fong & Christian, 2012 (47)	Gaydos et al. 2002 (48)
		Gaydos et al., 2014 (49)	
Hjelm et al., 2001 (50)	Van Dommelen et al., 2011 (51)	Meyer, 2012 (52)	
Hsieh et al., 2003 (53)	Wiesenfeld et al., 1996 (54)	Sautter et al., 2009 (55)	

For peer review only

Keane et al., 2007 (56)	Witkin et al., 1996 (57)	Van der Pol et al. 2017 (58)	**Performance calculation not based on PIS**
Knox et al., 2002 (59)			Kellogg et al. 2004 (60)
Kriesel et al., 2016 (61)		**Paper not available/located**	Tayoun et al. 2015 (62)
Li et al.2014., (63)			
Lawton et al. 2013 (64)		Lanjouw et al., 2009 (65)	**Results not stratified by sample site**
Macmillan et al., 2003 (66)		Ostergaard et al., 1997 (67)	McKechnie et al., 2011 (68)
Macmillan et al., 2003(69)		Cheng et al., 2014 (70)	
Ostergaard & Moller, 1995 (71)			
Ostergaard et al. 1996 (72)		**Cervical & vaginal samples combined**	
Polaneczky et al., 1998 (73)			
Renton et al., 2006 (74)		Koumans et al., 2003 (75)	
Rompalo et al., 2001 (76)		Leslie et al., 2003 (77)	
Rumyantseva et al 2015 (78)			

For peer review only

Modified QUADAS-2 tool for assessment of included study quality

A modified tool, adapted from QUADAS-2 (79) was used to evaluate the following aspects of the included studies:

- **Risk of bias in patient selection:** Were patients selected at random (or in a consecutive manner of patients attending the clinic)? Were risk factors for the outcomes used to select patients included?
- **Risk of bias in patient infection status (PIS) definition:** Was PIS clearly defined? Had vaginal swabs been included in the PIS definition?
- **Risk of bias in flow and timing:** Is there a lag between the different tests done which could affect the results of the test? Were the same tests conducted for all patients (for example, was discrepant analysis used for equivocal test results?)?
- **Applicability concerns in patient selection:** Did the patient and the setting match the research question?
- **Applicability concerns for PIS definition:** Is the definition of PIS different from the review question?

Table S2. QUADAS-2 assessment for each included study

	Risk of bias			Applicability concerns	
Reference	Patient selection	PIS definition	Flow and timing	Patient selection	PIS definition
Chernesky *et al.*, 2006 (80)	Unclear	High	High	Unclear	Low
Chernesky *et al.*, 2014 (81)	Unclear	High	Low	Low	Low
Cosentino *et al.*, 2003 (82)	Unclear	High	High	High	Low
Gaydos *et al.*, 2010 (83)	Unclear	High	Unclear	Low	Low
Hook *et al.*, 1997 (84)	Unclear	High	High	Low	Low
Le Roy *et al.*, 2012 (85)	High	High	High	Low	High
Schachter *et al.*, 2005 (86)	Unclear	Low	Low	Low	Low
Shipitsyna *et al.*, 2009 (87)	High	High	Unclear	High	High
Stary et al., 1998 (88)	High	High	High	High	High

Overall, the sampling of patients to studies was not well described (unclear risk of bias) or selected patients with risk factors for the outcome (high risk of bias). All but Schachter *et al.* had included vaginal samples in their PIS definition which we considered likely to introduce a high risk of bias for the sensitivity estimate (as discussed in the manuscript).

For flow and timing, the majority of studies were judged to have high risk of bias as they had used discrepant analysis (additional tests performed on patients with equivocal results) in their analyses or they had a different testing for some of the patients. There were less applicability concerns as concerns about risk of bias. Applicability of patient selection was unclear if the patient selection was poorly defined and of high concern if the patient population was at increased risk for the outcome (reported symptoms or were contacts of infected partners or reported high risk behaviours). PIS definition was marked as high concern if it was poorly explained (Shipitsyna *et al.*) or the sensitivity estimate was not directly reported (Stary and Le Roy).

For peer review only

Table S3. Summary of results from studies that estimated sensitivity of vaginal specimens relative to PIS for chlamydia

Author and year	Number of women who had chlamydia test	Chlamydia infected as defined by PIS (prevalence)	Sample sensitivity relative to PIS (95% CI) [numbers reported by studies]		
			Vaginal samples	Endocervical samples	Urine
Chernesky M. et al. (2006)	298	69 (23%)	(AC2 against PIS) 99% (92-100%) [68/69] (PT against PIS) 65% (53-76%) [45/69] (AMP against PIS) 65% (52-76%) [45/69]	(AC2 against PIS) 90% (80-96%) [62/69] (PT against PIS) 64% (51-75%) [44/69] (AMP against PIS) 59% (47-71%) [41/69]	(AC2 against PIS) 81% (70-90%) [56/69] (PT against PIS) 67% (54-78%) [46/69] (AMP against PIS) 57% (44-68%) [39/69]
Chernesky M. *et al.*, (2014)	575	53 (9%)	AC2 (Tigris) 98.1 (90.1-99.7%) AC2 (Panther) 96.2 (87.3-99.0%)<br><br>Real*Time* CT/NG 98.0 (89.7-99.7%)<br><br>ProbeTec CT/GC Qx 90.6 (79.8-95.9%)<br><br>Cobas CT/NG 84.6 (72.5-92.0%)	n/a	AC2 (Tigris) 88.7 (77.4-94.7%) AC2 (Panther) 88.0 (76.2-94.4%)<br><br>Real*Time* CT/NG 76.9 (63.9—86.3%)<br><br>ProbeTec CT/GC Qx 75.5 (62.4-85.1%)<br><br>Cobas CT/NG 81.1 (68.6-89.4%)
Cosentino L.A. et al. (2003)	455	37 (8.1%)	(SDA against PIS) – SDA available for all sites 92% (78-98%) [34/37]	(SDA against PIS) 92% 78-98%) [34/37]	n/a
Gaydos C.A. et al. (2010)	2014 women enrolled	(Overall prevalence 9%)	Clinician-collected Estimate and range presented (ABT against PIS) Symptomatic : 93% (84–97%) Asymptomatic: 87% (74–95%)<br><br>(GP AC2 against PIS) Symptomatic: 93% (85–97%) Asymptomatic: 85% (72–94%)	(ABT against PIS) Estimate and range presented Symptomatic: 88% (79–94%) Asymptomatic: 81% (67–91%)<br><br>(GP AC2 against PIS) Symptomatic: 91% (83-97%) Asymptomatic: 79% (64–89%)	(ABT against PIS) Estimate and range presented Symptomatic: 93% (85–97%) Asymptomatic: 96% (85–100%)<br><br>(GP AC2 against PIS) Symptomatic: 94% (86–98%) Asymptomatic: 94% (82–99%)

For peer review only

Le Roy C. et al. (2012)	193	19 included in this analysis. Prevalence in the study was 20/193 (10%).	Bio-Rad Dx CT/NG/MG<br>100 % (82-100%) [19/19]<br><br>Cobas<br>100% (82-100%) [19/19]	Not evaluated as only 21 individuals had EC swab taken (3 Ct positives)	Bio-Rad Dx CT/NG/MG<br>100% (82-100%) [19/19]<br><br>Cobas<br>89% (67-99%) [17/19]
Schachter J et al. (2005)	1451	180<br>(12%)	Clinician-collected<br>Aptima Combo: 97%<br>Aptima CT: 97%<br><br>Patient-collected<br>Aptima Combo: 97%<br>Aptima CT: 98%	n/a	n/a
Shipitsyna E. et al. (2009)	298 (with both EC and vagina)	36<br>(12%)	100% (85-100%) [36/36]	97% (85-100%) [35/36]	n/a
Stary A et al. (1998)	308	25<br>(8%)	(TMA against PIS)<br>92% (74-99%) [23/25]<br><br>(LCR against PIS)<br>92%(74-99%) [23/25]	(TMA against PIS)<br>88% (69-97%) [22/25]<br><br>(LCR against PIS)<br>92% (74-99%) [23/25]	(TMA against PIS)<br>76% (55-91%) [19/25]<br><br>(LCR against PIS)<br>96% (80-100%) [24/25]

For peer review only - http://bmjopen.bmj.com/site/about/guidelines.xhtml

Table S4. **Summary of results from studies that estimated sensitivity of vaginal specimens relative to PIS for gonorrhoea**

Author and year	Number of women who had gonorrhoea test	Gonorrhoea infected as defined by PIS (prevalence)	Sample sensitivity relative to PIS		
			**Vaginal**	**Endocervical**	**Urine**
Chernesky M. *et al.*, (2014)	575	11 (2%)	AC2 (Tigris): 90.9%<br>AC2 (Panther): 100%<br>RealTime CT/NG: 70.0%<br>ProbeTec CT/GC Qx:100%<br>Cobas CT/NG:63.6%	n/a	AC2 (Tigris): 72.7%<br>AC2 (Panther): 66.7%<br>RealTime CT/NG: 70.0%<br>ProbeTec CT/GC Qx: 80.0%<br>Cobas CT/NG: 70.0%
Cosentino L.A. et al. (2003)	455	39 (8.6%)	100% (91-100%) [39/39]	100% (91-100%) [39/39]	n/a
Gaydos C.A. et al. (2010)	2014 women enrolled	Overall prevalence 3.8%	Clinician-collected<br>Estimate and range presented (ABT (RealTime) against PIS)<br>Symptomatic: 97% (83–100%)<br>Asymptomatic: 96% (78–100%)<br><br>(GP AC2 against PIS)<br>Symptomatic: 94% (79–99%)<br>Asymptomatic: 96% (78–100%)	Estimate and range presented (ABT (RealTime) against PIS)<br>Symptomatic: 87% (70–96%)<br>Asymptomatic: 91% (72–99%)<br><br>(GP AC2 against PIS)<br>Symptomatic: 91% (75–98%)<br>Asymptomatic: 91% (71%–99%)	Estimate and range presented (ABT (RealTime) against PIS)<br>Symptomatic: 94% (79–99%)<br>Asymptomatic: 87% (66–97%)<br><br>(GP AC2 against PIS)<br>Symptomatic: 84% (67–95%)<br>Asymptomatic:83% (61-95%)
Hook, E et al. (1997)	309	52 (16.8%)	Patient-obtained<br>100% (93-100%) [52/52]	85% (72-93%) [44/52]	
Le Roy et al. (2012)	193	2 (1%)	Not evaluated.		
Schachter J et al. (2005)	1450	78 (5.4%)	Clinician-collected<br>Aptima Combo: 96%<br>Aptima GC: 96%<br><br>Patient-collected<br>Aptima Combo: 99%<br>Aptima GC: 96%		

For peer review only - http://bmjopen.bmj.com/site/about/guidelines.xhtml

References

1. Bakken IJ, Bratt H, Skjeldestad FE, Nordbø SA. 2005. [Detection of chlamydia trachomatis in urine, vulval and cervical swabs]. Tidsskr den Nor lægeforening Tidsskr Prakt Med ny række 125:1629–30.

2. Schoeman SA, Stewart CMW, Booth RA, Smith SD, Wilcox MH, Wilson JD. 2012. Assessment of best single sample for finding chlamydia in women with and without symptoms: a diagnostic test study. BMJ 345:e8013.

3. Jaton K, Bille J, Greub G. 2006. A novel real-time PCR to detect Chlamydia trachomatis in first-void urine or genital swabs. J Med Microbiol 55:1667–1674.

4. Andrews J. 2009. Self-administered vaginal swabs were accurate for screening for sexually transmitted infections in the emergency department. Evid Based Med 14:150.

5. Behets FM-TF, Andriamiadana J, Randrianasolo D, Rasamilalao D, Ratsimbazafy N, Dallabetta G, Cohen MS. 2002. Laboratory diagnosis of sexually transmitted infections in women with genital discharge in Madagascar: implications for primary care. Int J STD AIDS 13:606–611.

6. Schmitt M, Depuydt C, Stalpaert M, Pawlita M. 2014. Bead-based multiplex sexually transmitted infection profiling. J Infect 69:123–133.

7. Le Roy C, Papaxanthos A, Liesenfeld O, Mehats V, Clerc M, Bébéar C, de Barbeyrac B. 2013. Swabs (dry or collected in universal transport medium) and semen can be used for the detection of Chlamydia trachomatis using the cobas 4800 system. J Med Microbiol 62:217–22.

8. Berwald N, Cheng S, Augenbraun M, Abu-Lawi K, Lucchesi M, Zehtabchi S. 2009. Self-administered vaginal swabs are a feasible alternative to physician-assisted cervical swabs for sexually transmitted infection screening in the emergency department. Acad Emerg Med 16:360–3.

9. Shrier L, Dean D, Klein E, Harter K, Rice P. 2004. Limitations of Screening Tests for the Detection of Chlamydia trachomatis in Asymptomatic Adolescent and Young Adult Women. Obs Gynecol Surv 59:513–515.

10. Shalepo K, Savicheva A, Shipitsyna E, Unemo M, Domeika M. 2006. Diagnosis of Chlamydia trachomatis in Russia - In-house PCR assays may be effective but overall optimization and quality assurance are urgently needed. Apmis 114:500–507.

11. Black CM, Driebe EM, Howard L a, Fajman NN, Sawyer MK, Girardet RG, Sautter RL, Greenwald E, Beck-Sague CM, Unger ER, Igietseme JU, Hammerschlag MR, MD P, Igietseme JU, Hammerschlag MR, C.M. B-SB, E.M. D, L.A. H, N.N. F, M.K. S, R.G. G, R.L. S, E. G, E.R. U, J.U. I, M.R. H. 2009. Multicenter study of nucleic acid amplification tests for detection of Chlamydia trachomatis and Neisseria gonorrhoeae in children being evaluated for sexual abuse. Pediatr Infect Dis J 28:608–613.

12. Skidmore S, Horner P, Herring A, Sell J, Paul I, Thomas J, Caul EO, Egger M, McCarthy E, Sanford E, Salisbury C, Macleod J, Sterne J, Low L, Group CSS (ClaSS) P. 2006. Vulvovaginal-swab or first-catch urine specimen to detect Chlamydia trachomatis in women in a community setting?. J Clin Microbiol 44:4389–4394.

13. Schachter J, McCormack WM, Chernesky MA, Martin DH, Van Der Pol B, Rice PA, Hook EW 3rd, Stamm WE, Quinn TC, Chow JM. 2003. Vaginal swabs are appropriate specimens for diagnosis of genital tract infection with Chlamydia trachomatis. J Clin Microbiol 41:3784–3789.

14. Bachmann LLH, Desmond RA, Stephens J, Hughes A, Hook EW, Stephens R, Hughes A, Hook III EW. 2002. Duration of persistence of gonococcal DNA detected by ligase chain reaction in men and women following recommended therapy for uncomplicated gonorrhea. J Clin Microbiol 40:3596–3601.

For peer review only - http://bmjopen.bmj.com/site/about/guidelines.xhtml

15. Carder C, Robinson AJ, Broughton C, Stephenson JM, Ridgway GL. 1999. Evaluation of self-taken samples for the presence of genital Chlamydia trachomatis infection in women using the ligase chain reaction assay. Int J STD AIDS 10:776–779.

16. Stewart CMWCMW, Schoeman SA, Booth RA, Smith SDSDD, Wilcox MHMHH, Wilson JDJDD. 2012. Assessment of self taken swabs versus clinician taken swab cultures for diagnosing gonorrhoea in women: single centre, diagnostic accuracy study. BMJ 345:e8107.

17. Samra Z, Rosenberg S, Madar-Shapiro L. 2011. Direct simultaneous detection of 6 sexually transmitted pathogens from clinical specimens by multiplex polymerase chain reaction and auto-capillary electrophoresis. Diagn Microbiol Infect Dis 70:17–21.

18. Chandeying V, Skov S, Farrell D, Lamlertkittikul S, Tabrizi S, Jarumanokul R. 2002. GYNAECOLOGY A Comparison of First-Void Urine , Self-Administered Low Vaginal Swab , Self-Inserted Tampon , and Endocervical Swab Using PCR Tests for the Detection of Infection with Chlamydia Trachomatis in Commercial Sex Workers and Women Attending Gyneco 14:201–209.

19. Sturm PDJ, Connolly C, Khan N, Ebrahim S, Sturm a W. 2004. Vaginal tampons as specimen collection device for the molecular diagnosis of non-ulcerative sexually transmitted infections in antenatal clinic attendees. Int J STD AIDS 15:94–8.

20. Upton A, Wilson J, Bissessor L. 2013. Introduction of routine polymerase chain reaction testing for Neisseria gonorrhoeae in a community laboratory. Sex Health. ELEC, CSIRO (P.O. Box 1139, Collingwood VIC 3066, Australia), A. Upton, Labtests, 27-41 Carbine Road, Mt Wellington 1060, New Zealand. E-mail: arlo.upton@labtests.co.nz.

21. Cheng A, Kirby JE. 2014. Evaluation of the hologic gen-probe PANTHER, APTIMA combo 2 assay in a tertiary care teaching hospital. Am J Clin Pathol 141:397–403.

22. Tanaka M, Nakayama H, Yoshida H, Takahashi K, Nagafuji T, Hagiwara T, Kumazawa J. 1998. Detection of Chlamydia trachomatis in vaginal specimens from female commercial sex workers using a new improved enzyme immunoassay. Sex Transm Infect 74:435–438.

23. Vahidnia A, Costa S, Veenings S, Tuin H, van Loon L, Bliekendaal H. 2014. Comparative evaluation of Roche Aurora FLOW, Becton and Dickinson Viper system, and Dynex DS2 for detection of Chlamydia trachomatis, Neisseria gonorrhoeae, and Trichomonas vaginalis in various clinical specimens. Diagn Microbiol Infect Dis 80:191–192.

24. Dietrich W, Rath M, Stanek G, Apfalter P, Huber JC, Tempfer C. 2010. Multiple site sampling does not increase the sensitivity of Chlamydia trachomatis detection in infertility patients. Fertil Steril 93:68–71.

25. Tanaka M, Nakayama H, Sagiyama K, Haraoka M, Yoshida H, Hagiwara T, Akazawa K, Naito S. 2000. Evaluation of a new amplified enzyme immunoassay (EIA) for the detection of Chlamydia trachomatis in male urine , female endocervical swab , and patient obtained vaginal swab specimens. J Clin Pathol 53:350–354.

26. Yu B, An Y, Xu G, Shan H. 2016. Detection of *Chlamydia trachomatis* and *Neisseria gonorrhoea* e based on cross-priming amplification. Lett Appl Microbiol 62:399–403.

27. Domeika M, Drulyte O. 2000. Use of PCR for the detection of genital Chlamydia trachomatis infection on self-obtained mailed vaginal samples. Acta Obstet Gynecol Scand 79:570–575.

28. Taylor SN, Van Der Pol B, Lillis R, Hook EW 3rd, Lebar W, Davis T, Fuller D, Mena L, Fine P, Gaydos CA, Martin DH. 2011. Clinical evaluation of the BD ProbeTecTM Chlamydia trachomatis Qx amplified DNA assay on the BD ViperTM system with XTRTM technology. Sex Transm Dis 38:603–609.

For Peer review only

29. Zauli DAG, De Menezes CLP, De Oliveira CL. 2013. Development and padronization of three multiplex PCRs for the diagnosis of chlamydia trachomatis, toxoplasma gondii, herpes simplex viruses 1 and 2, and cytomegalovirus. Mol Biotechnol 54:1004–1009.

30. Domeika M, Bassiri M, Butrimiene I, Venalis a, Ranceva J, Vasjanova V. 1999. Evaluation of vaginal introital sampling as an alternative approach for the detection of genital Chlamydia trachomatis infection in women. Acta Obstet Gynecol Scand 78:131–136.

31. Thomas BJ, Pierpoint T, Taylor-Robinson D, Renton M. 1998. Sensitivity of the ligase chain reaction assay for detecting Chlamydia trachomatis in vaginal swabs from women who are infected at other sites. Sex Transm Infect 74:140–1.

32. Carroll KC, Aldeen WE, Morrison M, Anderson R, Lee D, Mottice S. 1998. Evaluation of the Abbott LCx ligase chain reaction assay for detection of Chlamydia trachomatis and Neisseria gonorrhoeae in urine and genital swab specimens from a sexually transmitted disease clinic population. J Clin Microbiol 36:1630–1633.

33. Falk L, Coble B-I, Mjörnberg P, Fredlund H. 2010. Sampling for Chlamydia trachomatis infection - a comparison of vaginal, first-catch urine, combined vaginal and first-catch urine and endocervical sampling. Int J STD AIDS 21:283–287.

34. Thomas BJ, Pierpoint T, Taylor-Robinson D, Renton M. 2001. Qualitative and quantitative aspects of the ligase chain reaction assay for Chlamydia trachomatis in genital tract samples and urines. Int J STD AIDS 12:589–594.

35. Lee GI, Yoen JP, Kang JS, Hwang SY, Hong YM, Yang JH, Yoon HK. 2013. A comparison of oligonucleotide-based microarray and real-time PCR for the detection of sexually transmitted infections. Biochip J 7:68–74.

36. Fang J, Husman C, DeSilva L, Chang R, Peralta L. 2008. Evaluation of self-collected vaginal swab, first void urine, and endocervical swab specimens for the detection of Chlamydia trachomatis and Neisseria gonorrhoeae in adolescent females. J Pediatr Adolesc Gynecol 21:355–360.

37. Van Der Pol B, Taylor SN, Lebar W, Davis T, Fuller D, Mena L, Fine P, Gaydos C a., Martin DH, Hook EW. 2012. Clinical Evaluation of the BD ProbeTec^{TM} Neisseria gonorrhoeae Qx Amplified DNA Assay on the BD Viper^{TM} System With XTR^{TM} Technology. Sex Transm Dis 39:147–153.

38. Nuñez-Forero L, Moyano-Ariza L, Gaitán-Duarte H, Ángel-Müller E, Ruiz-Parra A, González P, Rodríguez A, Tolosa JE. 2016. Diagnostic accuracy of rapid tests for sexually transmitted infections in symptomatic women. Sex Transm Infect 92:24–28.

39. Fernández G, Martró E, González V, Saludes V, Bascuñana E, Marcó C, Rivaya B, López E, Coll P, Matas L, Ausina V. 2016. Usefulness of a novel multiplex real-time PCR assay for the diagnosis of sexually-transmitted infections. Enferm Infecc Microbiol Clin 34:471–6.

40. Garrow SC, Smith DW, Harnett GB. 2002. The diagnosis of chlamydia, gonorrhoea, and trichomonas infections by self obtained low vaginal swabs, in remote northern Australian clinical practice. Sex Transm Infect 78:278–281.

41. Van Der Pol B, Taylor SN, Liesenfeld O, Williams JA, Hook EW. 2013. Vaginal swabs are the optimal specimen for detection of genital Chlamydia trachomatis or Neisseria gonorrhoeae using the Cobas 4800 CT/NG test. Sex Transm Dis 40:247–50.

42. Gaydos CA, Van Der Pol B, Jett-Goheen M, Barnes M, Quinn N, Clark C, Daniel GE, Dixon PB, Hook EW 3rd, Group CS. 2013. Performance of the Cepheid CT/NG Xpert Rapid PCR Test for Detection of Chlamydia trachomatis and Neisseria gonorrhoeae. J Clin Microbiol 51:1666–1672.

For peer review only

43. Van Der Pol B, Hook 3rd EW, Williams JA, Smith B, Taylor SN. 2015. Performance of the BD CTQx and GCQx Amplified Assays on the BD Viper LT Compared With the BD Viper XTR System. Sex Transm Dis 42:521–523.

44. CHERNESKY MA, JANG DA. 2006. APTIMA transcription-mediated amplification assays for Chlamydia trachomatis and Neisseria gonorrhoeae. Expert Rev Mol Diagn 6:519–525.

45. Gokral JS, Mania-Pramanik J, Meherji PK, Mali BN. Introital swab testing for Chlamydia trachomatis in a resource-poor setting: an Indian perspective. Int J Fertil Womens Med 50:140–3.

46. Van Der Pol B, Williams JA, Fuller DA, Taylor SN, Hook EW. 2017. Combined testing for chlamydia, gonorrhea, and trichomonas by use of the BD max CT/GC/TV assay with genitourinary specimen types. J Clin Microbiol 55:155–164.

47. Fong H, Christian CW. 2012. Evaluating Sexually Transmitted Infections in Sexually Abused Children: New Techniques to Identify Old Infections. Clin Pediatr Emerg Med 13:202–212.

48. Gaydos C, Crotchfelt K, Shah N, Tennant M, Quinn TC, Gaydos JC, McKee KT, Rompalo AM. 2002. Evaluation of dry and wet transported intravaginal swabs in detection of Chlamydia trachomatis and Neisseria gonorrhoeae infections in female soldiers by PCR. J Clin Microbiol 40:758–761.

49. Gaydos CA. 2014. Review of use of a new rapid real-time PCR, the Cepheid GeneXpert ® (Xpert) CT/NG assay, for *Chlamydia trachomatis* and *Neisseria gonorrhoeae* : results for patients while in a clinical setting. Expert Rev Mol Diagn 14:135–137.

50. Hjelm E, Hallén a., Domeika M. 2001. Cervical, urine and vaginal specimens for detection of Chlamydia trachomatis by ligase chain reaction in women: A comparison. Acta Derm Venereol 81:285–288.

51. van Dommelen L, Dukers-Muijrers N, Van Tiel F, Brouwers E, Hoebe C. 2011. Evaluation of one-sample testing of self-obtained vaginal swabs and first-catch urine samples separately and in combination for the detection of Chlamydia trachomatis by two amplified DNA assays in women visiting a sexually transmitted disease clinic. Clin Microbiol Infect 17:S754–S755.

52. Meyer T, T. M. 2012. [Diagnosis and treatment of Chlamydia trachomatis infections]. Hautarzt 63:16–23.

53. Hsieh Y-H, Howell MR, Gaydos JC, Mckee KT, Quinn TC, Gaydos C a, MD MsPH, Mckee KT, Quinn TC, Gaydos C a. 2003. Preference among female Army recruits for use of self-administrated vaginal swabs or urine to screen for Chlamydia trachomatis genital infections. Sex Transm Dis 30:769–773.

54. Wiesenfeld HC, Heine RP, Rideout a., Macio I, DiBiasi F, Sweet RL. 1996. The vaginal introitus: A novel site for Chlamydia trachomatis testing in women. Am J Obstet Gynecol 174:1542–1546.

55. Sautter RL, LeBar WD, Greenwald E. 2009. The Laboratory’s Role in Evaluating Sexually Transmitted Diseases as a Result of Sexual Abuse. Clin Microbiol Newsl 31:145–150.

56. Keane F, Bendall R, Saulsbury N, Haddon L. 2007. A comparison of self-taken vulvovaginal and cervical samples for the diagnosis of Chlamydia trachomatis infection by polymerase chain reaction. Int J STD AIDS 18:98–100.

57. Witkin SS, Inglis SR, Polaneczky M. 1996. Detection of Chlamydia trachomatis and Trichomonas vaginalis by polymerase chain reaction in introital specimens from pregnant women. Am J Obstet Gynecol 175:165–167.

For peer review only

58. Van Der Pol B. 2017. Profile of the triplex assay for detection of chlamydia, gonorrhea and trichomonas using the BD MAX^{TM} System. Expert Rev Mol Diagn 17:539–547.

59. Knox J, Tabrizi SN, Miller P, Petoumenos K, Law M, Chen S, Garland SM. 2002. Evaluation of self-collected samples in contrast to practitioner-collected samples for detection of Chlamydia trachomatis, Neisseria gonorrhoeae, and Trichomonas vaginalis by polymerase chain reaction among women living in remote areas. Sex Transm Dis 29:647–54.

60. Kellogg ND, Baillargeon J, Lukefahr JL, Lawless K, Menard SW. 2004. Comparison of nucleic acid amplification tests and culture techniques in the detection of Neisseria gonorrhoeae and Chlamydia trachomatis in victims of suspected child sexual abuse. J Pediatr Adolesc Gynecol 17:331–339.

61. Kriesel JD, Bhatia AS, Barrus C, Vaughn M, Gardner J, Crisp RJ. 2016. Multiplex PCR testing for nine different sexually transmitted infections. Int J STD AIDS 27:1275–1282.

62. Abou Tayoun AN, Burchard PR, Caliendo AM, Scherer A, Tsongalis GJ. 2015. A multiplex PCR assay for the simultaneous detection of Chlamydia trachomatis, Neisseria gonorrhoeae, and Trichomonas vaginalis. Exp Mol Pathol 98:214–218.

63. Li J, Jang D, Gilchrist J, Smieja M, Ewert R, MacRitchie C, Chernesky M. 2014. Comparison of flocked and Aptima swabs and two specimen transport media in the Aptima combo 2 assay. J Clin Microbiol 52:3808–3809.

64. Lawton B, Bromhead C. 2013. Nucleic acid amplification tests of self-taken vulvovaginal swabs are more sensitive than clinician taken endocervical culture for gonorrhoea. Evid Based Med 18:e46.

65. Lanjouw E, van Daele PLA, Raes MPE, van der Meijden WI. 2009. Consecutively Acquired Sexually Transmitted Infections Mimicking Crohn's Disease. Am J Gastroenterol 104:532–533.

66. Macmillan S, McKenzie H, Templeton A, Strandell A. 2003. Screening for chlamydial infection by DNA testing in urine and vulval swabs was highly sensitive and more acceptable than endocervical swabs. Evidence-based Obstet Gynecol 5:120–121.

67. Ostergaard LJ, Moller JK, Andersen B, Olesen F. 1997. Self-collected and mailed samples for diagnosis of urogenital Chlamydia trachomatis infection in women [Danish]. Ugeskr Laeger 159:5089–92.

68. McKechnie ML, Hillman RJ, Jones R, Lowe PC, Couldwell DL, Davies SC, King F, Kong F, Gilbert GL. 2011. The prevalence of urogenital micro-organisms detected by a multiplex PCR-reverse line blot assay in women attending three sexual health clinics in Sydney, Australia. J Med Microbiol 60:1010–1016.

69. Macmillan S, McKenzie H, Templeton A. 2003. Parallel observation of four methods for screening women under 25 years of age for genital infection with Chlamydia trachomatis. Eur J Obstet Gynecol Reprod Biol 107:68–73.

70. Cheng P, Tang J, Cheng H, Liu X, Wang Y. 2014. PCR methods for simultaneous detection of Neisseria agonorrhoeae, Chlamydiatrachomatis and Ureaplasma parvum. Chinese J Biol 27:109–114.

71. Ostergaard L, Møller JK. 1995. Use of PCR and direct immunofluorescence microscopy for confirmation of results obtained by Syva MicroTrak Chlamydia enzyme immunoassay. J Clin Microbiol 33:2620–3.

72. Ostergaard L, Møller JK, Andersen B, Olesen F. 1996. Diagnosis of urogenital Chlamydia trachomatis infection in women based on mailed samples obtained at home: multipractice comparative study. BMJ 313:1186–9.

73. Polaneczky M, Quigley C, Pollock L, Dulko D, Witkin SS. 1998. Use of self-collected

For peer review only

vaginal specimens for detection of Chlamydia trachomatis infection. Obstet Gynecol 91:375–8.

74. Renton A, Thomas BM, Gill S, Lowndes C, Taylor-Robinson D, Patterson K. 2006. Chlamydia trachomatis in cervical and vaginal swabs and urine specimens from women undergoing termination of pregnancy. Int J STD AIDS 17:443–447.

75. Koumans EH, Black CM, Markowitz LE, Unger ER, Pierce A, Sawyer MK, Papp JR. 2003. Comparison of Methods for Detection of Chlamydia trachomatis and Neisseria gonorrhoeae Using Commercially Available Nucleic Acid Amplification Tests and a Liquid Pap Smear Medium. Society 41:1507–1511.

76. Rompalo AM, Gaydos CA, Shah N, Tennant M, Crotchfelt KA, Madico G, Quinn TC, Daniel R, Shah K V, Gaydos JC, McKee KT. 2001. Evaluation of use of a single intravaginal swab to detect multiple sexually transmitted infections in active-duty military women. Clin Infect Dis 33:1455–1461.

77. Leslie DE, Azzato F, Ryan N, Fyfe J. 2003. An assessment of the Roche Amplicor ® gonorrhoeae multiplex PCR assay in routine diagnostic use on a variety of specimen types. Commun Dis Intell Q Rep 27:373–9.

78. Rumyantseva T, Golparian D, Nilsson CS, Johansson E, Falk M, Fredlund H, Van Dam A, Guschin A, Unemo M. 2015. Evaluation of the new AmpliSens multiplex real-time PCR assay for simultaneous detection of Neisseria gonorrhoeae, Chlamydia trachomatis, Mycoplasma genitalium, and Trichomonas vaginalis. Apmis 123:879–886.

79. Whiting PF, Rutjes AWS, Westwood ME, Mallett S, Deeks JJ, Reitsma JB, Leeflang MMG, Sterne JAC, Bossuyt PMM. 2011. QUADAS-2: a revised tool for the quality assessment of diagnostic accuracy studies. Ann Intern Med 155:529–36.

80. Chernesky MA, Jang D, Luinstra K, Chong S, Smieja M, Cai W, Hayhoe B, Portillo E, Main C, Ewert R, Macritchie C, Main C, Ewert R, Main MacRitchie C, Ewert R. 2006. High analytical sensitivity and low rates of inhibition may contribute to detection of Chlamydia trachomatis in significantly more women by the APTIMA Combo 2 assay. J Clin Microbiol 44:400–405.

81. Chernesky M, Jang D, Gilchrist J, Hatchette T, Poirier A, Flandin JF, Smieja M, Ratnam S. 2014. Head to Head Comparison of Second Generation Nucleic Acid Amplification Tests for Chlamydia trachomatis and Neisseria gonorrhoeae on Female Urines and Self-Collected Vaginal Swabs. J Clin Microbiol 52:2305–10.

82. Cosentino LA, Landers D V, Hillier SL. 2003. Detection of Chlamydia trachomatis and Neisseria gonorrhoeae by strand displacement amplification and relevance of the amplification control for use with vaginal swab specimens. J Clin Microbiol 41:3592–6.

83. Gaydos CA, Cartwright CP, Colaninno P, Welsch J, Holden J, Ho SY, Webb EM, Anderson C, Bertuzis R, Zhang L, Miller T, Leckie G, Abravaya K, Robinson J. 2010. Performance of the Abbott RealTime CT/NG for detection of Chlamydia trachomatis and Neisseria gonorrhoeae. J Clin Microbiol 48:3236–43.

84. Hook EW 3rd, Ching SF, Stephens J, Hardy KF, Smith KR, Lee HH. 1997. Diagnosis of Neisseria gonorrhoeae infections in women by using the ligase chain reaction on patient-obtained vaginal swabs. J Clin Microbiol 35:2129–2132.

85. Le Roy C, Le Hen I, Clerc M, Arfel V, Normandin F, Bebear C, de Barbeyrac B. 2012. The first performance report for the Bio-Rad Dx CT/NG/MG assay for simultaneous detection of Chlamydia trachomatis, Neisseria gonorrhoeae and Mycoplasma genitalium in urogenital samples. J Microbiol Methods 89:193–197.

86. Schachter J, Chernesky MA, Willis DE, Fine PM, Martin DH, Fuller D, Jordan JA, Janda W, Hook EW. 2005. Vaginal swabs are the specimens of choice when screening for Chlamydia trachomatis and Neisseria gonorrhoeae: results from a

For peer review only

multicenter evaluation of the APTIMA assays for both infections. Sex Transm Dis 32:725–8.

87. Shipitsyna E, Zolotoverkhaya E, Agne-Stadling I, Krysanova A, Savicheva A, Sokolovsky E, Domeika M, Unemo M. 2009. First evaluation of six nucleic acid amplification tests widely used in the diagnosis of Chlamydia trachomatis in Russia. J Eur Acad Dermatology Venereol 23:268–276.

88. Stary A, Schuh E, Kerschbaumer M, Götz B, Lee H. 1998. Performance of transcription-mediated amplification and ligase chain reaction assays for detection of chlamydial infection in urogenital samples obtained by invasive and noninvasive methods. J Clin Microbiol 36:2666–70.

For peer review only

PRISMA 2009 Checklist

Section/topic	#	Checklist item	Reported on page #
**TITLE**			
Title	1	Identify the report as a systematic review, meta-analysis, or both.	1
**ABSTRACT**			
Structured summary	2	Provide a structured summary including, as applicable: background; objectives; data sources; study eligibility criteria, participants, and interventions; study appraisal and synthesis methods; results; limitations; conclusions and implications of key findings; systematic review registration number.	2-3
**INTRODUCTION**			
Rationale	3	Describe the rationale for the review in the context of what is already known.	4-6
Objectives	4	Provide an explicit statement of questions being addressed with reference to participants, interventions, comparisons, outcomes, and study design (PICOS).	6-7
**METHODS**			
Protocol and registration	5	Indicate if a review protocol exists, if and where it can be accessed (e.g., Web address), and, if available, provide registration information including registration number.	6
Eligibility criteria	6	Specify study characteristics (e.g., PICOS, length of follow-up) and report characteristics (e.g., years considered, language, publication status) used as criteria for eligibility, giving rationale.	6-7
Information sources	7	Describe all information sources (e.g., databases with dates of coverage, contact with study authors to identify additional studies) in the search and date last searched.	7, Supplemental file
Search	8	Present full electronic search strategy for at least one database, including any limits used, such that it could be repeated.	Supplemental file
Study selection	9	State the process for selecting studies (i.e., screening, eligibility, included in systematic review, and, if applicable, included in the meta-analysis).	7-8
Data collection process	10	Describe method of data extraction from reports (e.g., piloted forms, independently, in duplicate) and any processes for obtaining and confirming data from investigators.	8
Data items	11	List and define all variables for which data were sought (e.g., PICOS, funding sources) and any assumptions and simplifications made.	8
Risk of bias in individual studies	12	Describe methods used for assessing risk of bias of individual studies (including specification of whether this was done at the study or outcome level), and how this information is to be used in any data synthesis.	8, Supplemental file
Summary measures	13	State the principal summary measures (e.g., risk ratio, difference in means).	8

For peer review only

PRISMA 2009 Checklist

Synthesis of results	14	Describe the methods of handling data and combining results of studies, if done, including measures of consistency (e.g., I^2) for each meta-analysis.	8

Page 1 of 2

Section/topic	#	Checklist item	Reported on page #
Risk of bias across studies	15	Specify any assessment of risk of bias that may affect the cumulative evidence (e.g., publication bias, selective reporting within studies).	8, Supplemental file
Additional analyses	16	Describe methods of additional analyses (e.g., sensitivity or subgroup analyses, meta-regression), if done, indicating which were pre-specified.	NA
**RESULTS**			
Study selection	17	Give numbers of studies screened, assessed for eligibility, and included in the review, with reasons for exclusions at each stage, ideally with a flow diagram.	8-9 Figure 1 Supplemental file
Study characteristics	18	For each study, present characteristics for which data were extracted (e.g., study size, PICOS, follow-up period) and provide the citations.	Tables 1 – 2 Supplemental file, Tables S3-4
Risk of bias within studies	19	Present data on risk of bias of each study and, if available, any outcome level assessment (see item 12).	Supplemental file, Table S2
Results of individual studies	20	For all outcomes considered (benefits or harms), present, for each study: (a) simple summary data for each intervention group (b) effect estimates and confidence intervals, ideally with a forest plot.	Figures 2-3
Synthesis of results	21	Present results of each meta-analysis done, including confidence intervals and measures of consistency.	NA
Risk of bias across studies	22	Present results of any assessment of risk of bias across studies (see Item 15).	14, Supplemental file
Additional analysis	23	Give results of additional analyses, if done (e.g., sensitivity or subgroup analyses, meta-regression [see Item 16]).	NA
**DISCUSSION**			
Summary of evidence	24	Summarize the main findings including the strength of evidence for each main outcome; consider their relevance to key groups (e.g., healthcare providers, users, and policy makers).	17-18

For peer review only

For peer review only - http://bmjopen.bmj.com/site/about/guidelines.xhtml

PRISMA 2009 Checklist

Limitations	25	Discuss limitations at study and outcome level (e.g., risk of bias), and at review-level (e.g., incomplete retrieval of identified research, reporting bias).	18
Conclusions	26	Provide a general interpretation of the results in the context of other evidence, and implications for future research.	19
**FUNDING**			
Funding	27	Describe sources of funding for the systematic review and other support (e.g., supply of data); role of funders for the systematic review.	20

From: Moher D, Liberati A, Tetzlaff J, Altman DG, The PRISMA Group (2009). Preferred Reporting Items for Systematic Reviews and Meta-Analyses: The PRISMA Statement. PLoS Med 6(7): e1000097. doi:10.1371/journal.pmed1000097

For more information, visit: **www.prisma-statement.org**.

For peer review only

For peer review only - http://bmjopen.bmj.com/site/about/guidelines.xhtml

BMJ Open

Evaluation of the performance of nucleic acid amplification tests (NAATs) in detection of chlamydia and gonorrhoea infection in vaginal specimens relative to patient infection status: a systematic review

Journal:	*BMJ Open*
Manuscript ID	bmjopen-2018-022510.R1
Article Type:	Research
Date Submitted by the Author:	04-Jul-2018

Complete List of Authors:	Rönn, Minttu; Imperial College London, Department of Infectious Disease Epidemiology; Harvard T. H. Chan School of Public Health, Department of Global Health and Population<br>Mc Grath-Lone, Louise; Imperial College London, Department of Infectious Disease Epidemiology<br>Davies, Bethan; Imperial College London, Department of Infectious Disease Epidemiology<br>Wilson, Janet; Leeds General Infirmary,<br>Ward, Helen; Imperial College London, Department of Infectious Disease Epidemiology

**Primary Subject Heading**:	Infectious diseases
Secondary Subject Heading:	Sexual health, Diagnostics, Evidence based practice
Keywords:	chlamydia, gonorrhea, patient infection status, Molecular diagnostics < INFECTIOUS DISEASES

SCHOLARONE™ Manuscripts

Evaluation of the performance of nucleic acid amplification tests (NAATs) in detection of chlamydia and gonorrhoea infection in vaginal specimens relative to patient infection status: a systematic review

AUTHORS:

Minttu M. Rönn[1,2], Louise Mc Grath-Lone[1], Bethan Davies[1], Janet D. Wilson[3], Helen Ward[1]

1 Department of Infectious Disease Epidemiology, Imperial College London, London, UK
2. Department of Global Health and Population, Harvard T.H. Chan School of Public Health
3 Leeds Sexual Health, Leeds Teaching Hospitals NHS Trust, Leeds, UK

Corresponding Author:
Minttu M. Rönn
Imperial College London
St Mary's Campus
Norfolk Place
W2 1PG London

MMR: mronn@hsph.harvard.edu
LM: lmcgrathlone@gmail.com
BD: bethan.davies06@imperial.ac.uk
JDW: janet-d.wilson@nhs.net
HW: h.ward@imperial.ac.uk

Abstract word count: 263
Manuscript word count: 3140
References: 33
Tables: 1 (in the manuscript)
Figures: 3 (attached)
Supplemental material: 1 (attached)

Keywords: chlamydia, gonorrhea, diagnostics, patient infection status

For peer review only

ABSTRACT

OBJECTIVES: We aimed to assess the performance of NAATs using vaginal specimens in comparison to other urogenital specimens in their ability to detect chlamydia and gonorrhea infection in women.

SETTING: We conducted a systematic review using EMBASE and Ovid MEDLINE databases for eligible studies. We compared the performance of NAATs on specimens taken from the vagina to those from the cervix or urine in diagnosing chlamydia and gonorrhoea infection in women based on patient infection status (PIS).

PARTICIPANTS: We included studies that tested samples from the vagina and ≥1 other site (cervix and/or urine) with ≥2 NAATs for chlamydia and ≥2 NAATs or 1 NAAT and culture for gonorrhoea for each site.

PRIMARY AND SECONDARY OUTCOME MEASURES: We analysed the performance using vaginal specimens compared to PIS. Performance is defined as the sensitivity of a NAAT using a specimen site and PIS status of the patient. We assessed risk of bias using modified QUADAS-2.

RESULTS: Nine publications met the inclusion criteria (eight for chlamydia; six for gonorrhoea) and were narratively reviewed. Pooled summary estimates were not calculated due to the variable methodology and PIS definitions. Tests performed on vaginal specimens accomplished similar performance to cervical and urine specimens for chlamydia (range of performance estimates: vaginal 65-100%, cervical 59-97%, urine 57-100%) and gonorrhoea (vaginal 64-100%, cervical 85-100%, urine 67-94%). Vaginal specimens were estimated to have a performance >80% for chlamydia and gonorrhoea infections in all but one study.

For peer review only - http://bmjopen.bmj.com/site/about/guidelines.xhtml

CONCLUSIONS: Performance of the NAATs for chlamydia and gonorrhea detection using vaginal specimens was similar to that of cervical and urine specimens relative to PIS. As vaginal samples have a higher acceptability and lower cost, the study can support clinical testing guidelines by providing evidence that vaginal samples are a suitable alternative to traditional test sites.

ARTICLE SUMMARY

Strengths and limitations of this study

- The systematic review included studies which had used a conservative definition of patient infection status (PIS), which can improve estimates of performance.
- The study included a broad time frame and the search did not have language restrictions, which increased the scope of the literature search.
- The lack of standardised definitions of PIS and variable methodologies used restricted our analyses.

For peer review only - http://bmjopen.bmj.com/site/about/guidelines.xhtml

INTRODUCTION

In the UK, practices for *Chlamydia trachomatis* (chlamydia) and *Neisseria gonorrhoea* (gonorrhoea) testing are not currently standardised. Nucleic acid amplification tests (NAATs) are recommended for the diagnosis of chlamydia and gonorrhoea due to their high sensitivity and specificity (1–3), but a variety of urogenital specimens (i.e. urine, cervical and vaginal) are used for testing at clinic level. This is an issue because the performance of NAATs to detect infection is known to vary by the type of specimen that is tested. A systematic review by Cook *et al.* (2005) estimated that the overall performance of NAATs for gonorrhoea was just 55.6% (95% CI: 36.3-74.9%) for urine samples compared to 94.2% (90.5-98.0%) for cervical samples, with a specificity of 98.7% (97.5-99.9%) and 99.2% (98.4-100%), respectively (4). Sensitivity is typically defined as the probability that a diagnostic test provides a positive result given that the the true test result – using gold standard – is positive, and a specificity is the probability that a diagnostic test yields a negative result given the true test result – using gold standard – is negative.

Recent studies have suggested that vaginal swabs may me better at detecting chlamydia and gonorrhoea than the traditionally-used urine and cervical samples (5–14). An issue related to these studies is that the estimates may be overestimated due to the use of discrepant analysis. As there is no gold-standard diagnostic test for either chlamydia or gonorrhoea (i.e. one with 100% sensitivity and specificity), studies evaluating the performance of a novel NAAT have tended to compare its performance against presumably poorer performing existing NAATs. Given the assumption that the novel NAAT has a higher sensitivity, it would be reasonable to expect that it would yield a positive result in some cases where the existing NAAT

For peer review only - http://bmjopen.bmj.com/site/about/guidelines.xhtml

yields a negative result. The discrepant results create a challenge in differentiating false positive results from true positive results for the novel NAAT. In this situation, a third test is often performed. If this third test is positive then the positive result from the novel NAAT is defined a “true positive” and if the third test is negative the result from the novel NAAT is defined a “false positive”. This approach is called discrepant analysis (DA). DA has been criticized because it only retests “false positives” and doesn’t seek to improve the classification of “false negatives” which potentially introduces a bias towards overestimating the performance of the novel NAAT (15).

To address some of the potential bias from DA, patient infection status (PIS) was developed to represent an “extended gold-standard” by measuring test performance based on information from multiple sources. The performance of PIS can be thought of as the sensitivity of a NAAT from a given site given the patient is considered to have an infection (determined by the sites and definition used for PIS). There is no consensus definition for PIS, but in general, it involves performing ≥1 test(s) at >1 site and interpreting the results using an algorithm that describes the number of positive tests (by test type or site) needed to define a person as infected. A limitation of the current evidence base is that many studies that have concluded in favour of using vaginal samples over cervical or urine for the diagnosis of chlamydia and gonorrhoea have used just one NAAT per test site (6, 9–14). Ideally, PIS is determined with the results of multiple NAATs adjusting for potential problems with the NAATs as well as for the possibility that a person might be infected at one or more anatomical sites (16). PIS is not a perfect reference-standard, and it has received criticism for similar reasons as discrepant analysis whereby there is a conditional dependence in the estimation of test performance and the PIS (17). This

For peer review only - http://bmjopen.bmj.com/site/about/guidelines.xhtml

may result in overestimation of the sensitivity of a test from a given site. but by including multiple sites in the PIS it can also result in lower site-specific performance. PIS allows us to compare different sites within a study and similar PIS definition allow for comparison between studies. The ultimate use depends on the best combination of specimen and test. In most countries, health funding restraints mean it is only possible to use one specimen for chlamydia and gonorrhra testing in women, and therefore it is important to know which sites to sampmle to diagnosed the highest proportion of infections.

The overall comparison of vaginal test performance compared to cervical and urine tests warrants further evaluation to inform evidence-based clinical testing guidelines. To this end, we performed a systematic review of the published literature. The primary aim of this review was to compare the performance of specimens taken from the vagina to those from the cervix or urine in diagnosing chlamydia and gonorrhoea infection in women based on a PIS definition. Given the lack of calibrated gold-standard diagnostic tests and variation in the definition of PIS in the literature, we purposively used conservative selection criteria to identify studies that applied rigorous testing criteria in the process of defining PIS. We also examined the quality of the studies in light of the known biases in the PIS methodology to increase our ability to assess the evidence-base.

METHODS

We performed a systematic review following the Preferred Reporting Items for Systematic Reviews and Meta-Analyses (PRISMA) guidelines (18). Protocol for our review was not registered in advance. We included studies which (a) tested for

For peer review only - http://bmjopen.bmj.com/site/about/guidelines.xhtml

urogenital chlamydia and/or gonorrhoea infection in (b) post-pubertal women (c) using a vaginal specimen (collected by any method) and (d) at least one other urine or cervical specimen. We limited this review to studies that used a conservative approach for defining PIS whereby each specimen site had to be tested with (e) ≥2 NAATs for chlamydia and ≥2 NAATs or 1 NAAT plus culture for gonorrhoea (as culture is an accepted alternative to NAAT for gonorrhoea diagnosis (19)). Finally, studies had to (f) report the performance of vaginal specimens compared to the PIS, or give the numbers of patients infected and results from the tests so that this could be calculated.

NAATs were defined as commercially available or in-house tests involving DNA or RNA replication using polymerase chain reaction (PCR), transcription mediated amplification (TMA), ligase chain reaction (LCR) or strand displacement amplification (SDA). Studies that used pooled samples or novel point-of-care tests were excluded together with studies that used one NAAT on all samples and only confirmed positive or equivocal results with a second NAAT. No restrictions were set on year or language of publication. However, only peer-reviewed studies were included; conference abstracts/proceedings, notes, letters and book chapters were excluded.

The electronic databases EMBASE (1947 to 2017) and Ovid MEDLINE (1946 to 2017) were searched on 3 October 2017 by two authors, using OvidSP as the search platform (MMR, LM or BD). The search strategy included keywords related to the infections, interventions, specimens of interest and outcomes. The full search strategy is presented in the supplement (File S1).

For peer review only

For peer review only - http://bmjopen.bmj.com/site/about/guidelines.xhtml

After duplicate references had been removed by OvidSP, two authors (MMR LM, or BD) independently selected studies by scanning titles and abstracts. A list of potentially eligible publications was obtained through consensus agreement and full texts were obtained online, through the British Library or directly from the authors. The full texts were then reviewed for inclusion and a third author was consulted if there was disagreement between the two reviewers. Where the language of the article was not English we consulted colleagues fluent in the relevant language. The reference lists of all studies meeting the inclusion criteria, as well as recent key papers and systematic reviews identified during the search process, were examined iteratively for additional references.

One author extracted information from the selected studies using a data extraction form in Microsoft Excel. The extracted data was checked against the studies by a second author. Two reviewers independently assessed the risk of bias in patient selection, PIS definition and flow and timing (of different tests and analysis, including here also discrepant analysis) for each included study using an adapted version of the quality assessment of diagnostic accuracy studies (QUADAS-2) tool (20). The purpose of this quality assessment was to consider the potential biases and variation within the individual studies that may account for heterogeneity across the studies. For each study, we calculated or reported the sensitivity of each NAAT for each sample site, and calculated confidence intervals using the exact binomial distribution. If the study did not present raw numbers, the summary estimate was extracted instead. Performance was defined as the "number of tests appearing to be a true positive at a site for a given NAAT / number of infected patients as determined by PIS". These performance measures were used to compare the ability of each sample

For peer review only - http://bmjopen.bmj.com/site/about/guidelines.xhtml

site to accurately predict PIS by describing the overall differences within and between sample sites for chlamydia and gonorrhoea. Analyses and forest plots were done in R(21).

Patient and Public Involvement

Vaginal specimens are an acceptable and sometimes a preferred way for chlamydia and gonorrhea testing among women.(22) In this study, we compare the evidence-base of performance of different testing sites for detection of chlamydia and gonorrhea by conducting a systematic review of published primary research. We did not involve patient groups in the design or conduct of the study. The findings of the study can be used to standardize testing practices.

RESULTS

The search on 3 October 2017 identified 567 studies of which 387 remained after de-duplication by the search platform OvidSP. We excluded 299 studies based on their abstracts and screened the full text of the remaining 89 studies of which one was identified through searching reference lists. We excluded 81 of these studies and the reasons for exclusion are presented in the supplement (File S1, Table S1). We identified one additional study through searching the reference lists (23) so that in total, nine eligible studies were included in this review: eight for chlamydia (24–31) and six for gonorrhoea (23, 25, 28, 30, 31). A flowchart describing study selection is presented in Figure 1 and a description of the included studies is presented in Table 1. Few studies included results that for patient characteristics or sample collection: three studies included self-collected vaginal swabs (23, 30, 31) and just one reported results for asymptomatic and symptomatic patients separately (25).

For peer review only - http://bmjopen.bmj.com/site/about/guidelines.xhtml

For peer review only

229 **Figure 1.** Flow chart of study selection.

230

For peer review only

For peer review only - http://bmjopen.bmj.com/site/about/guidelines.xhtml

231 **Table 1.**Characteristics of included studies

**Author and year (Reference) // Included infections**	**Review outcome the main focus of the study? // Aim of the study**	**Tests performed, (acronym for the NAAT)**	**Specimens/sites (in women) // Comments**	**PIS definition // Comments on discrepant analysis**
**Chernesky M. et al. (2006)** (27)<br><br>CT	No<br><br>To compare detection thresholds and inhibitor and infection rates from different specimens	APTIMA Combo 2, (TMA AC2)<br><br>ProbeTec ET, (SDA ProbeTec ET)<br><br>Amplicor, (PCR AMP)	3 EC, 3 CCVS and FCU divided into 3 aliquots<br><br>Each sample was tested as spiked (with added CT) or without added CT; seemingly all the tests were done to all the samples	≥1 site positive by ≥2 different tests or two specimens positive in a single test (≥2 out of 9 specimen-tests done); VS included in PIS<br><br>No discrepant analysis described
**Cherneskey M et al. (2014)** (31)<br><br>CT & NG	No<br><br>To compare the performance of four second-generation NAATs with FCU and VS	APTIMA Combo 2, (TMA AC2)<br><br>RealT*ime* CT/NG, (PCR RealT*ime*)<br><br>ProbeTec CT/GC Q^x, (SDA ProbeTec CT/GC Q^x)<br><br>Cobas CT/NG, (PCR Cobas CT/NG)	1 FCU and 4 SCVS<br><br>Each sample was tested as spiked (with added CT) or without added CT; all the tests were done to all the samples	≥2 of the 4 assays were positive for any specimen type; VS included in PIS<br><br>No discrepant analysis described
**Cosentino L.A. et al. (2003)** (28)<br><br>**CT & NG**	Yes<br><br>To compare vaginal swab specimens to endocervical swab specimens for the detection of CT and NG	ProbeTec ET, (SDA ProbeTec ET)<br><br>Amplicor, (PCR AMP)<br><br>Thayer-Martin medium and chocolate agar (for NG culture)	3 EC, 2 (presumably clinician-obtained) VS. One EC used for NG culture<br><br>CT was tested by PCR and SDA; NG (EC) was tested for by culture and SDA	Positive result by two different molecular tests for CT or for NG by culture or by two molecular tests; VS included in PIS<br><br>Discrepant analysis for discrodant results by LCR

For peer review only - http://bmjopen.bmj.com/site/about/guidelines.xhtml

		LCx, (LCR) for discrepant results		
**Gaydos C.A. et al. (2010)**<br>(25)<br><br>CT & NG	No<br><br>To compare the performance of the new RealT*ime* CT/NG assay with the Aptima Combo 2 assay	RealT*ime* CT/NG, (PCR RealT*ime*) – index<br><br>APTIMA Combo 2, (TMA AC2) – reference<br><br>ProbeTec ET, (SDA ProbeTec ET) – reference<br><br>NG culture	4 EC, 1 SCVS, 2 CCVS, 3 urine<br><br>Only one NAAT performed on the SCVS (this test was not used to define PIS); results for this are not presented	≥1 positive result by both of the 2 reference NAATs, additionally for NG if culture positive the subject was defined as infected. Infection absent if ≥1 reference NAAT was negative for all sample types<br><br>Discrepant analysis: for CT retested discordant results, for NG not done
**Hook, E et al. (1997)**<br>(23)<br><br>NG	Yes<br><br>To evaluate patient-obtained vaginal specimens tested with culture and LCR assays for NG compared to clinician-collected specimens	LCx, (LCR)<br><br>Modified Thayer-Martin medium (for NG culture)	3 SCVS, 3 EC<br>(one sample at each site not part of this study as processed for chlamydia)	Culture positive from either site; or LCR positive and culture negative with a positive confirmatory LCR; VS is included in PIS<br><br>Discrepant analysis with, alternative TMA with different target site to confirm discordant resutls
**Le Roy C. et al. (2012)**<br>(24)<br><br>CT & NG[a]	No, data extracted based on their reporting.<br><br>Determine clinical performance of Bio-Rad CT/NG/MG assay for detection of chlamydia, gonorrhoea and mycoplasma genitalium	Dx CT/NG/MG Assay, (qPCR) – index test<br><br>Cobas TaqMan CT, (qPCR TaqMan) - reference<br><br>NG culture	Symptomatic: 2 SCVS, 2 EC and 2 FCU. Asymptomatic: 2 SCVS and FCU.<br><br>More tests done on symptomatic patients, but all samples seem to have been treated the same.	Study definition: At least 2 positive results from either of the two assays. We determined PIS based on test results for FCU and VS (which were available for all patients, see supplement material for further information); all infected patients had ≥2 positive tests and a positive test at both sites; VS is included in PIS<br><br>Discrepant analysis used for discordant results
**Schachter J et al. (2005)**<br>(30)	Yes<br><br>To evaluate the	APTIMA CT, (TMA ACT)<br><br>APTIMA GC, (TMA AGC)	1 FCU, 1 SCVS, 1 CCVS, 2 EC swabs	Infected if either BD or AC2 were positive on FCU or EC . VS not included in PIS

For peer review only - http://bmjopen.bmj.com/site/about/guidelines.xhtml

**CT & NG**	performance of APTIMA assays on vaginal swabs for CT and NG	APTIMA Combo 2, (TMA AC2)<br><br>ProbeTec ET, (SDA ProbeTec ET)	All samples tested with three TMAs (2 for CT and 2 for NG) FCU and 1x EC swab were also tested with ProbeTec ET)	No discrepant analysis described
**Shipitsyna E. et al. (2008)** (26)<br><br>CT	No<br><br>To evaluate the performance of five PCRs and a recently introduced nucleic acid sequence-based amplification (NASBA) assay	Different 'in-house' PCRs tested: cPCR-DT, rtPCR-DT, cPCR-Ly, cPCR-Ep, rtPCR-Ep, real-time NASBA assay<br><br>Reference methods:<br>Amplicor, (PCR AMP)<br><br>LightMix, (PCR Lightmix)	Used the subsample who had 4 specimens collected each: 2 EC and 2 VS<br><br>All sample sites tested using reference NAAT and at least 3 other NAATs	Several (range) Russian PCRs used on the sample and the sample was considered true positive if a positive result by a Russian PCR was confirmed by the reference tests; VS included in PIS<br><br>Discrepant analysis for discordant results
**Stary A et al. (1998)** (29)<br><br>**CT**	No<br><br>To compare TMA assay with LCR assay in detection of CT in genital tract with different specimen types	Aptima CT, (TMA ACT)<br><br>LCx, (LCR)<br><br>McCoy cell culture<br><br>Direct-fluorescent antibody assay (DFA) or alternative TMA for confirming discrepant results	3 EC, 3 CCVS, FCU<br><br>EC and VS tested with LCR, TMA and culture. FCU tested with LCR and TMA	Positive culture at any site or positive result by both NAATs in one site, or one positive NAAT confirmed with discrepant analysis; VS included in PIS.<br><br>Discrepant analysis with DFA or TMA with different target site to confirm discordant results

a. Too few infections with gonorrhoea for analysis

CT: *Chlamydia trachomatis*; NG: *Neisseria gonorrhoea*

For peer review only

For peer review only - http://bmjopen.bmj.com/site/about/guidelines.xhtml

CCVS: Clinician-collected vaginal specimens; SCVS: Self-collected vaginal specimens; EC: endocervical swabs; FCU: first catch urine; VS: vaginal specimen

Tests used: LCR (Ligase Chain Reaction); PCR AMP (Polymerase Chain Reaction Amplicor); PCR Cobas CT/NG (Polymerase Chain Reaction Cobas CT/NG); PCR 'In house'; PCR RealTi*me* (Polymerase Chain Reaction RealTi*me*); qPCR TaqMan (Quantitative Polymerase Chain Reaction TaqMan); qPCR DxCT/NG/MG (Quantitative Polymerase Chain Reaction DxCT/NG/MG); PCR LightMix, (Polymerase Chain Reaction Lightmix); NASBA (Nucleic acid sequence-based amplification); SDA ProbeTec ET (Strand Displacement Amplification ProbeTec ET); SDA ProbeTec CT/GC Q^x, (Strand Displacement Amplification ProbeTec CT/GC Q^x); TMA AC2 (Transcription Mediated Amplification Aptima Combo-2); TMA ACT (Transcription Mediated Amplification Aptima CT); TMA AGC (Transcription Mediated Amplification Aptima GC)

Discrepant analysis: We define discrepant analysis to have occurred in sitautions where sample was positive for only one of the tests used. In these instances another test was done.

For peer review only - http://bmjopen.bmj.com/site/about/guidelines.xhtml

The factors considered in the modified QUADAS-2 assessment and the results are presented in the supplement (File S1, Table S2). All studies were rated as high or unclear risk for at least two of the five criteria related to risk of bias and applicability concerns; for example, risk for patient selection bias was either unclear (6/9) or high (3/9). Our inclusion criteria required there to be ≥2 NAATs for chlamydia and ≥2 NAATs or 1 NAAT plus culture for gonorrhoea performed at each anatomical site, but variation remained in the number and type of tests used in the PIS algorithms. The definitions included "≥2 positive tests out of a number of tests performed (regardless of the site)", "≥2 different tests positive at the same site", "any test positive in ≥2 different sites" and "≥2 different tests positive" (described in Table 1). In all but one (30), the risk of bias in the definition of PIS was considered high because the algorithm used was unclearly defined and/or included vaginal specimens (as the component whose performance is being evaluated should be independent of the gold-standard it is measured against).

Seven out of nine studies (with the exception of Chernesky *et al.* 2006 (27) and Chernesky *et al.* 2014 (31) had performed a different number of tests on a subsample of their study population where most of the studies (6/9) performed discrepant analysis for discordant results (for example, for sites where only one NAAT of many was positive). Due to the low number of studies identified, and their variable methods and definitions of PIS, we deemed pooling of the results to be inappropriate. Instead, we compared the ability of each sample site to predict PIS using forest plots of site-specific performance from each study by NAAT used.

For peer review only - http://bmjopen.bmj.com/site/about/guidelines.xhtml

Chlamydia

Of the eight studies that considered chlamydia test performance (24–31), two directly addressed the question in this review (28, 30) and the remainder aimed to test the sensitivity of a novel NAAT in a clinical setting (24–27, 29, 31). All eight studies were performed in high-income countries. Where a broader description of the population sample was reported, the studies had recruited patients from sexual health or reproductive health clinics, youth centres or primary care settings. One study specified that the sample population was high-risk for acquisition of sexually transmitted infections (29).

The reported chlamydia prevalence in the included studies ranged from 8.1% to 23.2% based on varying definitions of PIS. The number of infected women according to PIS ranged from 19 to 69 (with one study giving a point estimate without the raw data (30)). The site-specific performance estimates and their confidence intervals are presented in Figure 2 and the results are presented in table format in the supplement (File S1, Table S3). Across the studies vaginal specimens performed similarly to cervical and urine specimens within a study, which is evident when comparing results by study (figure 2) (performance range: vaginal 65-100%, cervical 59-97%, urine 57-100%). The lowest overall performance estimates were reported by Chernesky *et al* using a first generation, less sensitive, NAAT (2006) (27).

Figure 2. Performance estimates for chlamydia by showing the sensitivity of the NAAT on a given specimen site relative to PIS. The studies are referenced by the first author, and publication year, and colors are by study for clarity.

For peer review only - http://bmjopen.bmj.com/site/about/guidelines.xhtml

Figure legend:

Each study is represented by a colour and where results are stratified by test, sampling or symptom status, this has been included in the figure.

Tests used: LCR (Ligase Chain Reaction); PCR AMP (Polymerase Chain Reaction Ampicor); PCR Cobas CT/NG (Polymerase Chain Reaction Cobas CT/NG); PCR 'In house'; PCR RealTi*me* (Polymerase Chain Reaction RealTi*me*); qPCR TaqMan (Quantitative Polymerase Chain Reaction TaqMan); qPCR DxCT/NG/MG (Quantitative Polymerase Chain Reaction DxCT/NG/MG); PCR LightMix, (Polymerase Chain Reaction Lightmix); NASBA (Nucleic acid sequence-based amplification); SDA ProbeTec ET (Strand Displacement Amplification Probetec ET); SDA ProbeTec CT/GC Q^x, (Strand Displacement Amplification ProbeTec CT/GC Q^x); TMA AC2 (Transcription Mediated Amplification Aptima Combo-2); TMA ACT (Transcription Mediated Amplification Aptima CT); TMA AGC (Transcription Mediated Amplification Aptima GC.

Gonorrhoea

We identified six studies that tested for gonorrhoea (23–25, 28, 30, 31). Of these, five had a sufficient number of infected women according to PIS for performance to be calculated and were included in this analysis (23, 25, 28, 30, 31). The excluded study found two gonorrhoea cases in 193 tested individuals and these were not analysed further (24). Of the five remaining studies, three addressed the question in this review (23, 28, 30) whilst the remaining two compared the performance of a new assay (25, 31). As with chlamydia, all of the studies were performed in high-income countries.

The prevalence of gonorrhoea ranged from 2% to 16.8% in the five studies, based on PIS, as defined by the studies; the denominator used for performance estimates varied from 11 to 52, with one study only reporting point estimates (30). Figure 3

For peer review only - http://bmjopen.bmj.com/site/about/guidelines.xhtml

presents the forest plot and the results are presented in table format in the supplement (File S1, Table S4). The performance estimates at each site in the four studies were similar witin each study: performance range for vaginal samples was 63.6-100%, for cervical samples 85-100% and for urine samples 66.7-94%. Three studies (23, 25, 28) included performance estimates for cervical swabs and two studies had performance estimates for urine (25, 31). The lowest overall performance estimates were reported by Chernesky *et al.* (2014) (31).

Figure 3. Performance estimates for gonorrhea showing the sensitivity of the NAAT on a given specimen site relative to PIS. The studies are referenced by the first author, and publication year, and colors are by study for clarity.

Figure legend:

Each study is represented by a colour and where results are stratified by test, sampling or symptom status, this has been included in the figure.

Tests used: LCR (Ligase Chain Reaction); PCR AMP (Polymerase Chain Reaction Ampicor); PCR Cobas CT/NG (Polymerase Chain Reaction Cobas CT/NG); PCR 'In house'; PCR RealTi*me* (Polymerase Chain Reaction RealTi*me*); qPCR TaqMan (Quantitative Polymerase Chain Reaction TaqMan); qPCR DxCT/NG/MG (Quantitative Polymerase Chain Reaction DxCT/NG/MG); PCR LightMix, (Polymerase Chain Reaction Lightmix); NASBA (Nucleic acid sequence-based amplification); SDA ProbeTec ET (Strand Displacement Amplification Probetec ET); SDA ProbeTec CT/GC Q^x, (Strand Displacement Amplification ProbeTec CT/GC Q^x); TMA AC2 (Transcription Mediated Amplification Aptima Combo-2); TMA ACT (Transcription Mediated Amplification Aptima CT); TMA AGC (Transcription Mediated Amplification Aptima GC.

DISCUSSION

For peer review only - http://bmjopen.bmj.com/site/about/guidelines.xhtml

We conducted a systematic review to evaluate the evidence for the relative performance of vaginal specimens in the detection of chlamydia and gonorrhoea in women using studies with conservative definitions of PIS. In the studies included in this review, we found similar sensitivities between urine, cervical and vaginal specimen types. This finding is contrary to other studies which have found that vaginal specimens have a higher performance than cervical swabs (5, 7, 9, 32). This difference may be due to the less conservative definition of PIS used in other studies, which may be more prone for overestimation. Nevertheless, our study suggests that vaginal swabs are at least as good as urine and cervical swabs in detecting chalmydia and gonorrhea.

This study followed PRISMA guidelines for systematic reviews and assessed the quality of the included studies using a modified version of QUADAS-2. The systematic searches were conducted by two authors and the results and discussion represent consensus opinion based on group discussion. There were no language or time restrictions and multiple databases were searched. Therefore, this review represents a robust and comprehensive summary of extant evidence on the comparative performance of vaginal specimens in detecting chlamydia and gonorrhoea infection. A further strength of this study is that we excluded studies where only one test was performed at the vaginal site, even if ≥2 NAATs were used at all other sites (6, 10, 33, 34). By limiting the analysis to studies that performed ≥2 tests at multiple sites, we aimed to minimize the potential for overestimation of test performance (16). The QUADAS-2 assessment found the studies were of variable quality and had methodological limitations and heterogeneity, particularly in PIS definition which was an important element of other analysis. As a result, some bias is

For peer review only - http://bmjopen.bmj.com/site/about/guidelines.xhtml

likely to remain in the estimates reported in this review and the performance estimates should not be directly compared across studies.

Overall, we found that vaginal specimens performed similarly to cervical and urine specimens for the diagnosis of both chlamydia and gonorrhoea in the identified studies. Based on the available evidence, and given the acceptability (22) and lower cost of vaginal samples (8), our study further supports that vaginal samples provide an appropriate alternative to traditional cervical or urine testing methods. However, this review has also highlighted that there are a lack of studies that have attempted to explicitly answer this research question whilst taking into account the inherent biases in estimating diagnostic test performance, particularly for NAATs. Further primary research with appropriate robust methodology for determining test performance in symptomatic and asymptomatic patients is needed for a more authoritative comparison of the performance of different urogenital sample sites. We suggest that research should be directed towards applying more sophisticated statistical methods for comparing test performance such as latent class modelling(17), which uses a weighted statistical model acknowledging the conditional dependence of the tests.

For peer review only

PATIENT CONSENT

Not required

DATA SHARING STATEMENT

All data presented in this study is available in the manuscript or in the supplementary material.

FUNDING

MMR received funding from the Wellcome Trust (G090285/Z/09/Z) during the first review. BD has been funded by an MRC Population Health Scientist Fellowship (G0902120). HW, BD and LM were supported in part by the National Institute for Health Research (NIHR) Biomedical Research Centre based at Imperial College Healthcare NHS Trust and Imperial College London. HW and JW have received funding from the NIHR Research for Patient Benefit (RfPB) Programme. The funders had no role in study design, data collection and analysis, decision to publish, or preparation of the manuscript.

CONFLICT OF INTEREST

JW has received research funding in the form of extra diagnostic reagents and equipment for one study from Hologic/Gen-Probe and has received one lecture fee from BD Diagnostics.

AUTHOR CONTRIBUTIONS

HW and JW conceived the study idea. MMR and LM conducted the first review and BD and MMR conducted the udate of the review with discrepancies solved by all three. MMR drafted the manuscript, which was subsequently edited by LM, BD, HW and JW. MMR prepared the tables and figures presented in the manuscript. All authors have read and accepted the final manuscript.

ACKNOWLEDGMENTS

The authors would like to thank Dr Mikaela Smit and Dr Annick Borquez for their help in evaluating and translating articles not available in English.

For peer review only

For peer review only - http://bmjopen.bmj.com/site/about/guidelines.xhtml

PRESENTATION AT A MEETING

Poster presented at 3rd Joint Conference of the British HIV Association, BHIVA with the British Association for Sexual Health and HIV, BASHH. Liverpool United Kingdom. Abstract: HIV Medicine 15 (pp 109-110), 2014.

For peer review only

For peer review only - http://bmjopen.bmj.com/site/about/guidelines.xhtml

REFERENCES

1. British Association for Sexual Health and HIV. 2015. Chlamydia trachomatis UK Testing Guidelines 1–17.
2. Bignell C, Fitzgerald M. 2011. UK national guideline for the management of gonorrhoea in adults, 2011. Int J STD AIDS 22:541–7.
3. Papp J, Schachter J, Gaydos C, Van Der Pol B. 2014. Recommendations for the laboratory-based detection of Chlamydia trachomatis and Neisseria gonorrhoeae--2014. MMWR Recomm Rep 63:1–19.
4. Cook RL, Hutchison SL, Østergaard L, Braithwaite RS, Ness RB. 2005. Systematic review: noninvasive testing for Chlamydia trachomatis and Neisseria gonorrhoeae. Ann Intern Med 142:914–25.
5. Schoeman SA, Stewart CMW, Booth RA, Smith SD, Wilcox MH, Wilson JD. 2012. Assessment of best single sample for finding chlamydia in women with and without symptoms: a diagnostic test study. BMJ 345:e8013.
6. Stewart CMWCMW, Schoeman SA, Booth RA, Smith SDSDD, Wilcox MHMHH, Wilson JDJDD. 2012. Assessment of self taken swabs versus clinician taken swab cultures for diagnosing gonorrhoea in women: single centre, diagnostic accuracy study. BMJ 345:e8107.
7. Shafer M, Moncada J, Boyer CB, Betsinger K, Flinn SD, Schachter J. 2003. Comparing first-void urine specimens, self-collected vaginal swabs, and endocervical specimens to detect Chlamydia trachomatis and Neisseria gonorrhoeae by a nucleic acid amplification test. J Clin Microbiol 41:4395–9.
8. Blake DR, Maldeis N, Barnes MR, Hardick A, Quinn TC, Gaydos CA. 2008. Cost-effectiveness of screening strategies for Chlamydia trachomatis using cervical swabs, urine, and self-obtained vaginal swabs in a sexually transmitted disease clinic setting. Sex Transm Dis 35:649–55.
9. Taylor SN, Van Der Pol B, Lillis R, Hook EW 3rd, Lebar W, Davis T, Fuller D, Mena L, Fine P, Gaydos CA, Martin DH. 2011. Clinical evaluation of the BD ProbeTecTM Chlamydia trachomatis Qx amplified DNA assay on the BD ViperTM system with XTRTM technology. Sex Transm Dis 38:603–609.
10. Van Der Pol B, Taylor SN, Liesenfeld O, Williams JA, Hook EW. 2013. Vaginal swabs are the optimal specimen for detection of genital Chlamydia trachomatis or Neisseria gonorrhoeae using the Cobas 4800 CT/NG test. Sex Transm Dis 40:247–50.
11. Falk L, Coble B-I, Mjörnberg P, Fredlund H. 2010. Sampling for Chlamydia trachomatis infection - a comparison of vaginal, first-catch urine, combined vaginal and first-catch urine and endocervical sampling. Int J STD AIDS 21:283–287.
12. Gaydos CA, Van Der Pol BB, Jett-Goheen M, Barnes M, Quinn N, Clark C, Daniel GE, Dixon PB, Hook EW 3rd, Group CS, Davis T, Pancholi P, Kerndt P, Horner P, Marrazzo J, De Burgh-Thomas A, Castro J, Ferguson D, Meyer M, Philip S, Van Der Pol BB, Thompson M, Taylor S, Ronk D, Fine P, Bachman L, Mayne D, Dietz C, Toevs K, Gettings N, Gall S, Patel R, Ross J, Brooks L, Hazan L, Clarke P, Groom R, Ackerman R, Anduczyk E, Mathews A, Blank S, Harris L, Tidings A, Moore P, Alexander S, Spurrell T, Group CS. 2013. Performance of the Cepheid CT/NG Xpert Rapid PCR Test for Detection of Chlamydia trachomatis and Neisseria gonorrhoeae. J Clin Microbiol 51:1666–1672.
13. Rompalo AM, Gaydos CA, Shah N, Tennant M, Crotchfelt KA, Madico G, Quinn TC, Daniel R, Shah K V, Gaydos JC, McKee KT. 2001. Evaluation of use of a single intravaginal swab to detect multiple sexually transmitted infections in active-duty military women. Clin Infect Dis 33:1455–1461.

For peer review only - http://bmjopen.bmj.com/site/about/guidelines.xhtml

14. van Dommelen L, Dukers-Muijrers N, Van Tiel F, Brouwers E, Hoebe C. 2011. Evaluation of one-sample testing of self-obtained vaginal swabs and first-catch urine samples separately and in combination for the detection of Chlamydia trachomatis by two amplified DNA assays in women visiting a sexually transmitted disease clinic. Clin Microbiol Infect 17:S754–S755.

15. Hadgu A. 1999. Discrepant analysis: a biased and an unscientific method for estimating test sensitivity and specificity. J Clin Epidemiol 52:1231–7.

16. Martin DH, Nsuami M, Schachter J, Iii EWH, Ferrero D, Quinn TC, Gaydos C, Hook EW 3rd, Ferrero D, Quinn TC, Gaydos C. 2004. Use of multiple nucleic acid amplification tests to define the infected-patient "gold standard" in clinical trials of new diagnostic tests for Chlamydia trachomatis infections. Society 42:4749–4758.

17. Hadgu A, Dendukuri N, Wang L. 2012. Evaluation of screening tests for detecting Chlamydia trachomatis: bias associated with the patient-infected-status algorithm. Epidemiology 23:72–82.

18. Moher D, Liberati A, Tetzlaff J, Altman DG, Group P. 2009. Preferred reporting items for systematic reviews and meta-analyses: the PRISMA statement. BMJ 339:b2535.

19. Bignell C, Unemo M. 2013. 2012 European guideline on the diagnosis and treatment of gonorrhoea in adults. Int J STD AIDS 24:85–92.

20. Whiting PF, Rutjes AWS, Westwood ME, Mallett S, Deeks JJ, Reitsma JB, Leeflang MMG, Sterne JAC, Bossuyt PMM. 2011. QUADAS-2: a revised tool for the quality assessment of diagnostic accuracy studies. Ann Intern Med 155:529–36.

21. R Core Team. 2017. R: A language and environment for statistical computing. R Foundation for Statistical Computing, Vienna, Austria.

22. Chernesky MA, Hook EW, Martin DH, Lane J, Johnson R, Jordan JA, Fuller D, Willis DE, Fine PM, Janda WM, Schachter J. 2005. Women find it easy and prefer to collect their own vaginal swabs to diagnose Chlamydia trachomatis or Neisseria gonorrhoeae infections. Sex Transm Dis 32:729–33.

23. Hook EW 3rd, Ching SF, Stephens J, Hardy KF, Smith KR, Lee HH. 1997. Diagnosis of Neisseria gonorrhoeae infections in women by using the ligase chain reaction on patient-obtained vaginal swabs. J Clin Microbiol 35:2129–2132.

24. Le Roy C, Le Hen I, Clerc M, Arfel V, Normandin F, Bebear C, de Barbeyrac B, Bébéar C, de Barbeyrac B. 2012. The first performance report for the Bio-Rad Dx CT/NG/MG assay for simultaneous detection of Chlamydia trachomatis, Neisseria gonorrhoeae and Mycoplasma genitalium in urogenital samples. J Microbiol Methods 89:193–197.

25. Gaydos CA, Cartwright CP, Colaninno P, Welsch J, Holden J, Ho SY, Webb EM, Anderson C, Bertuzis R, Zhang L, Miller T, Leckie G, Abravaya K, Robinson J. 2010. Performance of the Abbott RealTime CT/NG for detection of Chlamydia trachomatis and Neisseria gonorrhoeae. J Clin Microbiol 48:3236–43.

26. Shipitsyna E, Zolotoverkhaya E, Agne-Stadling I, Krysanova A, Savicheva A, Sokolovsky E, Domeika M, Unemo M. 2009. First evaluation of six nucleic acid amplification tests widely used in the diagnosis of Chlamydia trachomatis in Russia. J Eur Acad Dermatology Venereol 23:268–276.

27. Chernesky MA, Jang D, Luinstra K, Chong S, Smieja M, Cai W, Hayhoe B, Portillo E, Main C, Ewert R, Macritchie C, Main C, Ewert R, Main MacRitchie C, Ewert R. 2006. High analytical sensitivity and low rates of inhibition may contribute to detection of Chlamydia trachomatis in significantly more women by the APTIMA Combo 2 assay. J Clin Microbiol 44:400–405.

28. Cosentino LA, Landers D V, Hillier SL. 2003. Detection of Chlamydia trachomatis and Neisseria gonorrhoeae by strand displacement amplification and relevance of the

For peer review only - http://bmjopen.bmj.com/site/about/guidelines.xhtml

amplification control for use with vaginal swab specimens. J Clin Microbiol 41:3592–6.

29. Stary A, Schuh E, Kerschbaumer M, Götz B, Lee H. 1998. Performance of transcription-mediated amplification and ligase chain reaction assays for detection of chlamydial infection in urogenital samples obtained by invasive and noninvasive methods. J Clin Microbiol 36:2666–70.

30. Schachter J, Chernesky MA, Willis DE, Fine PM, Martin DH, Fuller D, Jordan JA, Janda W, Hook EW. 2005. Vaginal swabs are the specimens of choice when screening for Chlamydia trachomatis and Neisseria gonorrhoeae: results from a multicenter evaluation of the APTIMA assays for both infections. Sex Transm Dis 32:725–8.

31. Chernesky M, Jang D, Gilchrist J, Hatchette T, Poirier A, Flandin JF, Smieja M, Ratnam S. 2014. Head to Head Comparison of Second Generation Nucleic Acid Amplification Tests for Chlamydia trachomatis and Neisseria gonorrhoeae on Female Urines and Self-Collected Vaginal Swabs. J Clin Microbiol 52:2305–10.

32. Fang J, Husman C, DeSilva L, Chang R, Peralta L. 2008. Evaluation of self-collected vaginal swab, first void urine, and endocervical swab specimens for the detection of Chlamydia trachomatis and Neisseria gonorrhoeae in adolescent females. J Pediatr Adolesc Gynecol 21:355–360.

33. Koumans EH, Black CM, Markowitz LE, Unger ER, Pierce A, Sawyer MK, Papp JR. 2003. Comparison of Methods for Detection of Chlamydia trachomatis and Neisseria gonorrhoeae Using Commercially Available Nucleic Acid Amplification Tests and a Liquid Pap Smear Medium. Society 41:1507–1511.

34. Van Der Pol B, Williams JA, Fuller DA, Taylor SN, Hook EW. 2017. Combined testing for chlamydia, gonorrhea, and trichomonas by use of the BD max CT/GC/TV assay with genitourinary specimen types. J Clin Microbiol 55:155–164.

For peer review only

For peer review only - http://bmjopen.bmj.com/site/about/guidelines.xhtml

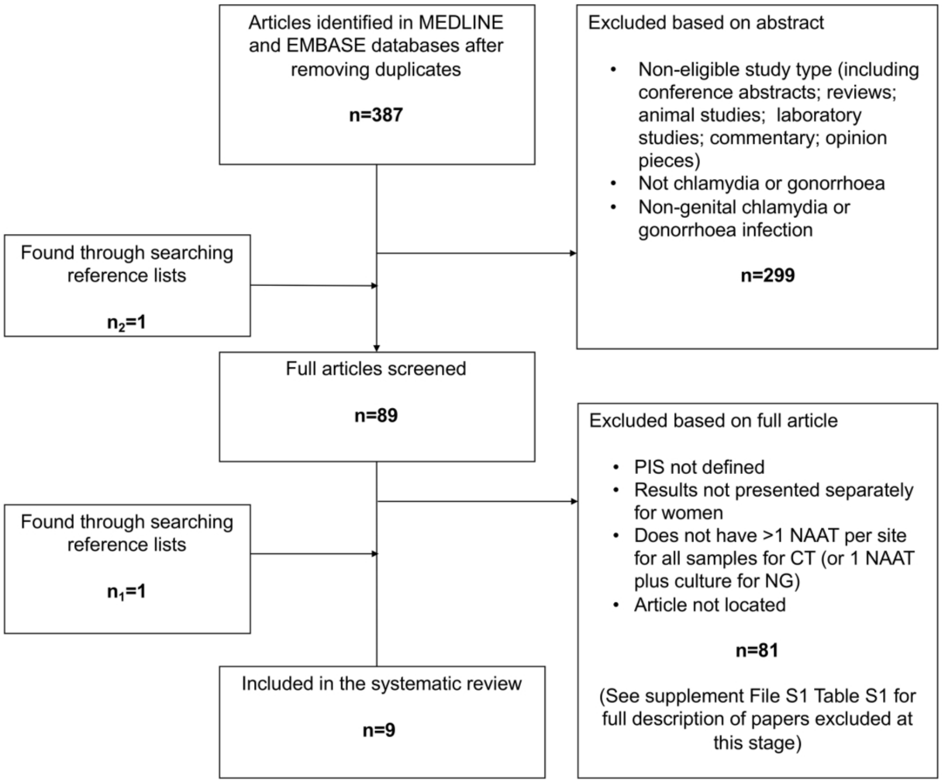

Figure 1. Flowchart of study selection

181x142mm (300 x 300 DPI)

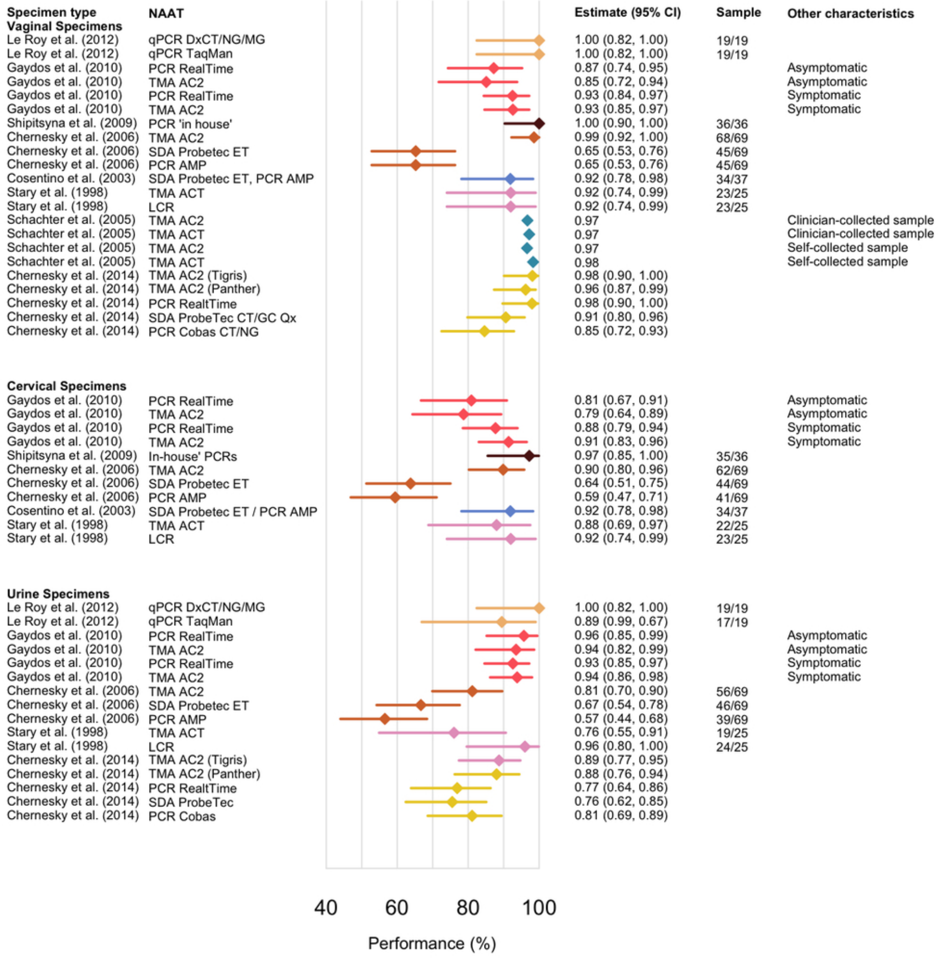

Figure 2. Performance estimates for chlamydia by showing the sensitivity of the NAAT on a given specimen site relative to PIS. The studies are referenced by the first author, and publication year, and colors are by study for clarity.

87x96mm (300 x 300 DPI)

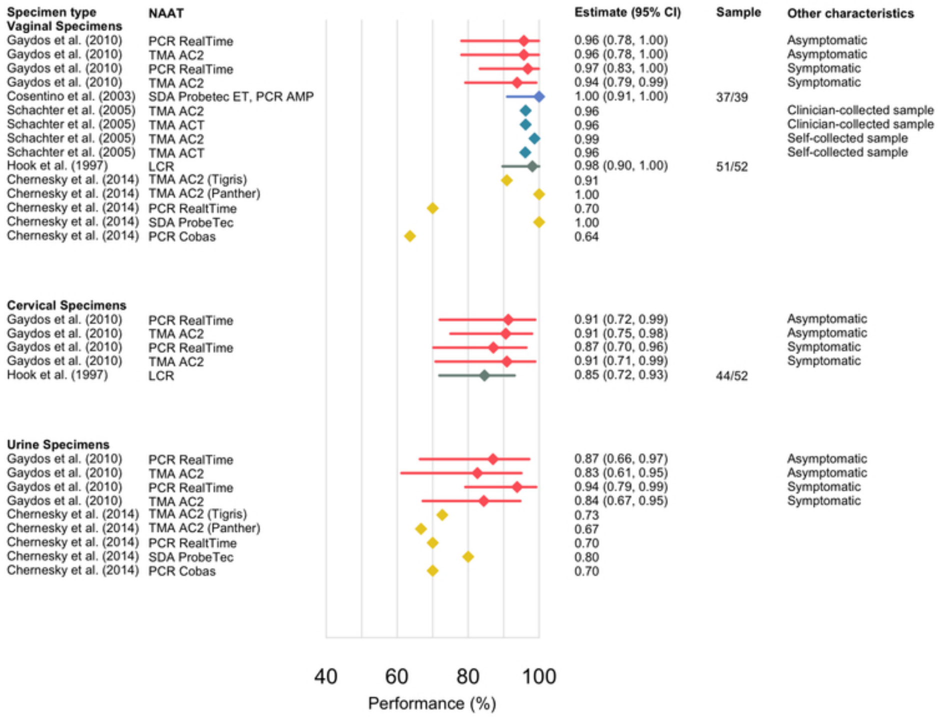

Figure 3. Performance estimates for gonorrhea showing the sensitivity of the NAAT on a given specimen site relative to PIS. The studies are referenced by the first author, and publication year, and colors are by study for clarity.

67x57mm (300 x 300 DPI)

Supplemental File for

Evaluation of the performance of nucleic acid amplification tests (NAATs) in detection of chlamydia and gonorrhoea infection in vaginal specimens relative to patient infection status: a systematic review

Minttu M. Rönn, Louise Mc Grath-Lone, Bethan Davies, Janet Wilson, Helen Ward

For peer review only

Search strategy

The following Boolean searches were performed on OvidSP on 8 October 2013 and an update on 3 October 2017. We searched all fields (af) using Ovid MEDLINE (1947-present) and EMBASE (1946-present) databases. We combined the individual searches with AND in the following manner:

(chlamydia OR "chlamydia trachomatis").af OR (gonorrh?ea OR "Neisseria gonorrhoeae").af

AND

(vagina$.af OR vulvo-vagina$).af

AND

(urine OR endovervi$ OR cervi$).af

AND

(NAAT OR "nucleic acid amplification" OR (PCR OR "polymerase chain reaction") OR (TMA OR "transcription mediated amplification OR "transcription-mediated amplification") OR (SDA or "Strand displacement amplification") OR (LCR or "ligase chain reaction")).af

AND

(sensitivity OR specificity OR performance).af

AND

(diagnos$ OR screen$ OR test OR detect$).af

Table S1.Studies which were excluded based on the full article (n=81).

**Does not have >1 NAAT per site for all samples for CT (or 1 NAAT plus culture for NG)**		**Performace calculation not based on PIS**	**Duplicate data**
Bakken et al., 2005 (1)	Schoeman et al., 2012 (2)	Jaton et al., 2006 (3)	Andrews, 2009 (4)
Behets et al., 2002 (5)	Schmitt et al, 2014 (6)	Le Roy et al., 2013 (7)	
Berwald et al., 2009(8)	Shrier et al., 2004 (9)	Shalepo et al., 2006 (10)	**Study population all infected**
Black et al., 2009 (11)	Skidmore et al., 2006 (12)	Schachter et al., 2003 (13)	Bachmann et al., 2002 (14)
Carder et al., 1999 (15)	Stewart et al., 2012 (16)	Samra et al., 2011 (17)	
Chandeying et al., 2004 (18)	Sturm et al., 2004 (19)	Upton et al., 2013 (20)	
Cheng et al. 2014 (21)	Tanaka et al., 1998 (22)	Vahidnia et al., 2014 (23)	
Dietrich et al., 2010 (24)	Tanaka et al., 2000 (25)	Yu et al., 2016(26)	**No vaginal samples**
Domeika & Drulyte, 2000 (27)	Taylor et al., 2011 (28)	Zauli et al., 2013 (29)	
Domeika et al.,1999 (30)	Thomas et al., 1998 (31)		Carroll et al. 1998 (32)
Falk et al., 2010 (33)	Thomas et al., 2001 (34)		Lee et al., 2013 (35)
Fang et al., 2008 (36)	Van Der Pol, 2012 (37)		Nunez-Forero et al. 2015 (38)
Fernandez et al., 2016 (39)	Van Der Pol et al., 2012 (28)		
Garrow et al., 2002 (40)	Van Der Pol et al., 2013 (41)	**No primary data reported**	
Gaydos et al., 2013 (42)	Van Der Pol et al., 2015 (43)	Chernesky & Jang, 2006 (44)	**Only vaginal samples**
Gokral et al., 2005 (45)	Van Der Pol et al., 2017 (46)	Fong & Christian, 2012 (47)	Gaydos et al. 2002 (48)
		Gaydos et al., 2014 (49)	
Hjelm et al., 2001 (50)	Van Dommelen et al., 2011 (51)	Meyer, 2012 (52)	
Hsieh et al., 2003 (53)	Wiesenfeld et al., 1996 (54)	Sautter et al., 2009 (55)	

For peer review only

Keane et al., 2007 (56)	Witkin et al., 1996 (57)	Van der Pol et al. 2017 (58)	**Performance calculation not based on PIS**
Knox et al., 2002 (59)			Kellogg et al. 2004 (60)
Kriesel et al., 2016 (61)		**Paper not available/located**	Tayoun et al. 2015 (62)
Li et al.2014., (63)			
Lawton et al. 2013 (64)		Lanjouw et al., 2009 (65)	**Results not stratified by sample site**
Macmillan et al., 2003 (66)		Ostergaard et al., 1997 (67)	McKechnie et al., 2011 (68)
Macmillan et al., 2003(69)		Cheng et al., 2014 (70)	
Ostergaard & Moller, 1995 (71)			
Ostergaard et al. 1996 (72)		**Cervical & vaginal samples combined**	
Polaneczky et al., 1998 (73)			
Renton et al., 2006 (74)		Koumans et al., 2003 (75)	
Rompalo et al., 2001 (76)		Leslie et al., 2003 (77)	
Rumyantseva et al 2015 (78)			

For peer review only

Modified Quality Assessment for Diagnostic Accuracy Studies (QUADAS-2) tool for assessment of included study quality

A modified tool, adapted from QUADAS-2 (79) was used to evaluate the following aspects of the included studies:

- **Risk of bias in patient selection:** Were patients selected at random (or in a consecutive manner of patients attending the clinic)? Were risk factors for the outcomes used to select patients included?
- **Risk of bias in patient infection status (PIS) definition:** Was PIS clearly defined? Had vaginal swabs been included in the PIS definition?
- **Risk of bias in flow and timing:** Is there a lag between the different tests done which could affect the results of the test? Were the same tests conducted for all patients (for example, was discrepant analysis used for equivocal test results?)?
- **Applicability concerns in patient selection:** Did the patient and the setting match the research question?
- **Applicability concerns for PIS definition:** Is the definition of PIS different from the review question?

Table S2. QUADAS-2 assessment for each included study

	Risk of bias			Applicability concerns	
Reference	Patient selection	PIS definition	Flow and timing	Patient selection	PIS definition
Chernesky *et al.*, 2006 (80)	Unclear	High	High	Unclear	Low
Chernesky *et al.*, 2014 (81)	Unclear	High	Low	Low	Low
Cosentino *et al.*, 2003 (82)	Unclear	High	High	High	Low
Gaydos *et al.*, 2010 (83)	Unclear	High	Unclear	Low	Low
Hook *et al.*, 1997 (84)	Unclear	High	High	Low	Low
Le Roy *et al.*, 2012 (85)	High	High	High	Low	High
Schachter *et al.*, 2005 (86)	Unclear	Low	Low	Low	Low
Shipitsyna *et al.*, 2009 (87)	High	High	Unclear	High	High
Stary et al., 1998 (88)	High	High	High	High	High

Overall, the sampling of patients to studies was not well described (unclear risk of bias) or selected patients with risk factors for the outcome (high risk of bias). All but Schachter *et al.* had included vaginal samples in their PIS definition which we considered likely to introduce a high risk of bias for the performace estimate (as discussed in the manuscript).

For flow and timing, the majority of studies were judged to have high risk of bias as they had used discrepant analysis (additional tests performed on patients with equivocal results) in their analyses or they had a different testing for some of the patients. There were less applicability concerns as concerns about risk of bias. Applicability of patient selection was unclear if the patient selection was poorly defined and of high concern if the patient population was at increased risk for the outcome (reported symptoms or were contacts of infected partners or reported high risk behaviours). PIS definition was marked as high concern if it was poorly explained (Shipitsyna *et al.*) or the performace estimate was not directly reported (Stary and Le Roy).

For peer review only - http://bmjopen.bmj.com/site/about/guidelines.xhtml

Table S3. Summary of results from studies that estimated performace of vaginal specimens relative to PIS for chlamydia

Author and year	Number of women who had chlamydia test	Chlamydia infected as defined by PIS (prevalence)	Sample performace relative to PIS (95% CI) [numbers reported by studies]		
			Vaginal samples	Endocervical samples	Urine
Chernesky M. et al. (2006)	298	69 (23%)	(AC2 against PIS)<br>99% (92-100%) [68/69]<br>(PT against PIS)<br>65% (53-76%) [45/69]<br>(AMP against PIS)<br>65% (52-76%) [45/69]	(AC2 against PIS)<br>90% (80-96%) [62/69]<br>(PT against PIS)<br>64% (51-75%) [44/69]<br>(AMP against PIS)<br>59% (47-71%) [41/69]	(AC2 against PIS)<br>81% (70-90%) [56/69]<br>(PT against PIS)<br>67% (54-78%) [46/69]<br>(AMP against PIS)<br>57% (44-68%) [39/69]
Chernesky M. *et al.*, (2014)	575	53 (9%)	AC2 (Tigris)<br>98.1 (90.1-99.7%)<br>AC2 (Panther)<br>96.2 (87.3-99.0%)<br><br>Real*Time* CT/NG<br>98.0 (89.7-99.7%)<br><br>ProbeTec CT/GC Qx<br>90.6 (79.8-95.9%)<br><br>Cobas CT/NG<br>84.6 (72.5-92.0%)	n/a	AC2 (Tigris)<br>88.7 (77.4-94.7%)<br>AC2 (Panther)<br>88.0 (76.2-94.4%)<br><br>Real*Time* CT/NG<br>76.9 (63.9—86.3%)<br><br>ProbeTec CT/GC Qx<br>75.5 (62.4-85.1%)<br><br>Cobas CT/NG<br>81.1 (68.6-89.4%)
Cosentino L.A. et al. (2003)	455	37 (8.1%)	(SDA against PIS) – SDA available for all sites<br>92% (78-98%) [34/37]	(SDA against PIS)<br>92% 78-98%) [34/37]	n/a
Gaydos C.A. et al. (2010)	2014 women enrolled	(Overall prevalence 9%)	Clinician-collected<br>Estimate and range presented<br>(ABT against PIS)<br>Symptomatic : 93% (84–97%)<br>Asymptomatic: 87% (74–95%)<br><br>(GP AC2 against PIS)<br>Symptomatic: 93% (85–97%)<br>Asymptomatic: 85% (72–94%)	(ABT against PIS)<br>Estimate and range presented<br>Symptomatic: 88% (79–94%)<br>Asymptomatic: 81% (67–91%)<br><br>(GP AC2 against PIS)<br>Symptomatic: 91% (83-97%)<br>Asymptomatic: 79% (64–89%)	(ABT against PIS)<br>Estimate and range presented<br>Symptomatic: 93% (85–97%)<br>Asymptomatic: 96% (85–100%)<br><br>(GP AC2 against PIS)<br>Symptomatic: 94% (86–98%)<br>Asymptomatic: 94% (82–99%)

For peer review only

For peer review only - http://bmjopen.bmj.com/site/about/guidelines.xhtml

Le Roy C. et al. (2012)	193	19 included in this analysis. Prevalence in the study was 20/193 (10%).	Bio-Rad Dx CT/NG/MG<br>100 % (82-100%) [19/19]<br><br>Cobas<br>100% (82-100%) [19/19]	Not evaluated as only 21 individuals had EC swab taken (3 Ct positives)	Bio-Rad Dx CT/NG/MG<br>100% (82-100%) [19/19]<br><br>Cobas<br>89% (67-99%) [17/19]
Schachter J et al. (2005)	1451	180<br>(12%)	Clinician-collected<br>Aptima Combo: 97%<br>Aptima CT: 97%<br><br>Patient-collected<br>Aptima Combo: 97%<br>Aptima CT: 98%	n/a	n/a
Shipitsyna E. et al. (2009)	298 (with both EC and vagina)	36<br>(12%)	100% (85-100%) [36/36]	97% (85-100%) [35/36]	n/a
Stary A et al. (1998)	308	25<br>(8%)	(TMA against PIS)<br>92% (74-99%) [23/25]<br><br>(LCR against PIS)<br>92%(74-99%) [23/25]	(TMA against PIS)<br>88% (69-97%) [22/25]<br><br>(LCR against PIS)<br>92% (74-99%) [23/25]	(TMA against PIS)<br>76% (55-91%) [19/25]<br><br>(LCR against PIS)<br>96% (80-100%) [24/25]

For peer review only

Table S4. **Summary of results from studies that estimated performace of vaginal specimens relative to PIS for gonorrhoea**

			**Sample performace relative to PIS**		
**Author and year**	**Number of women who had gonorrhoea test**	**Gonorrhoea infected as defined by PIS (prevalence)**	**Vaginal**	**Endocervical**	**Urine**
Chernesky M. *et al.*, (2014)	575	11<br>(2%)	AC2 (Tigris): 90.9%<br>AC2 (Panther): 100%<br>RealTime CT/NG: 70.0%<br>ProbeTec CT/GC Qx:100%<br>Cobas CT/NG:63.6%	n/a	AC2 (Tigris): 72.7%<br>AC2 (Panther): 66.7%<br>RealTime CT/NG: 70.0%<br>ProbeTec CT/GC Qx: 80.0%<br>Cobas CT/NG: 70.0%
Cosentino L.A. et al. (2003)	455	39<br>(8.6%)	100% (91-100%) [39/39]	100% (91-100%) [39/39]	n/a
Gaydos C.A. et al. (2010)	2014 women enrolled	Overall prevalence 3.8%	Clinician-collected<br>Estimate and range presented (ABT (RealTime) against PIS)<br>Symptomatic: 97% (83–100%)<br>Asymptomatic: 96% (78–100%)<br><br>(GP AC2 against PIS)<br>Symptomatic: 94% (79–99%)<br>Asymptomatic: 96% (78–100%)	Estimate and range presented (ABT (RealTime) against PIS)<br>Symptomatic: 87% (70–96%)<br>Asymptomatic: 91% (72–99%)<br><br>(GP AC2 against PIS)<br>Symptomatic: 91% (75–98%)<br>Asymptomatic: 91% (71%–99%)	Estimate and range presented (ABT (RealTime) against PIS)<br>Symptomatic: 94% (79–99%)<br>Asymptomatic: 87% (66–97%)<br><br>(GP AC2 against PIS)<br>Symptomatic: 84% (67–95%)<br>Asymptomatic:83% (61-95%)
Hook, E et al. (1997)	309	52<br>(16.8%)	Patient-obtained<br>100% (93-100%) [52/52]	85% (72-93%) [44/52]	
Le Roy et al. (2012)	193	2 (1%)	Not evaluated.		
Schachter J et al. (2005)	1450	78<br>(5.4%)	Clinician-collected<br>Aptima Combo: 96%<br>Aptima GC: 96%<br><br>Patient-collected<br>Aptima Combo: 99%<br>Aptima GC: 96%		

For peer review only

References

1. Bakken IJ, Bratt H, Skjeldestad FE, Nordbø SA. 2005. [Detection of chlamydia trachomatis in urine, vulval and cervical swabs]. Tidsskr den Nor lægeforening Tidsskr Prakt Med ny række 125:1629–30.

2. Schoeman SA, Stewart CMW, Booth RA, Smith SD, Wilcox MH, Wilson JD. 2012. Assessment of best single sample for finding chlamydia in women with and without symptoms: a diagnostic test study. BMJ 345:e8013.

3. Jaton K, Bille J, Greub G. 2006. A novel real-time PCR to detect Chlamydia trachomatis in first-void urine or genital swabs. J Med Microbiol 55:1667–1674.

4. Andrews J. 2009. Self-administered vaginal swabs were accurate for screening for sexually transmitted infections in the emergency department. Evid Based Med 14:150.

5. Behets FM-TF, Andriamiadana J, Randrianasolo D, Rasamilalao D, Ratsimbazafy N, Dallabetta G, Cohen MS. 2002. Laboratory diagnosis of sexually transmitted infections in women with genital discharge in Madagascar: implications for primary care. Int J STD AIDS 13:606–611.

6. Schmitt M, Depuydt C, Stalpaert M, Pawlita M. 2014. Bead-based multiplex sexually transmitted infection profiling. J Infect 69:123–133.

7. Le Roy C, Papaxanthos A, Liesenfeld O, Mehats V, Clerc M, Bébéar C, de Barbeyrac B. 2013. Swabs (dry or collected in universal transport medium) and semen can be used for the detection of Chlamydia trachomatis using the cobas 4800 system. J Med Microbiol 62:217–22.

8. Berwald N, Cheng S, Augenbraun M, Abu-Lawi K, Lucchesi M, Zehtabchi S. 2009. Self-administered vaginal swabs are a feasible alternative to physician-assisted cervical swabs for sexually transmitted infection screening in the emergency department. Acad Emerg Med 16:360–3.

9. Shrier L, Dean D, Klein E, Harter K, Rice P. 2004. Limitations of Screening Tests for the Detection of Chlamydia trachomatis in Asymptomatic Adolescent and Young Adult Women. Obs Gynecol Surv 59:513–515.

10. Shalepo K, Savicheva A, Shipitsyna E, Unemo M, Domeika M. 2006. Diagnosis of Chlamydia trachomatis in Russia - In-house PCR assays may be effective but overall optimization and quality assurance are urgently needed. Apmis 114:500–507.

11. Black CM, Driebe EM, Howard L a, Fajman NN, Sawyer MK, Girardet RG, Sautter RL, Greenwald E, Beck-Sague CM, Unger ER, Igietseme JU, Hammerschlag MR, MD P, Igietseme JU, Hammerschlag MR, C.M. B-SB, E.M. D, L.A. H, N.N. F, M.K. S, R.G. G, R.L. S, E. G, E.R. U, J.U. I, M.R. H. 2009. Multicenter study of nucleic acid amplification tests for detection of Chlamydia trachomatis and Neisseria gonorrhoeae in children being evaluated for sexual abuse. Pediatr Infect Dis J 28:608–613.

12. Skidmore S, Horner P, Herring A, Sell J, Paul I, Thomas J, Caul EO, Egger M, McCarthy E, Sanford E, Salisbury C, Macleod J, Sterne J, Low L, Group CSS (ClaSS) P. 2006. Vulvovaginal-swab or first-catch urine specimen to detect Chlamydia trachomatis in women in a community setting?. J Clin Microbiol 44:4389–4394.

13. Schachter J, McCormack WM, Chernesky MA, Martin DH, Van Der Pol B, Rice PA, Hook EW 3rd, Stamm WE, Quinn TC, Chow JM. 2003. Vaginal swabs are appropriate specimens for diagnosis of genital tract infection with Chlamydia trachomatis. J Clin Microbiol 41:3784–3789.

14. Bachmann LLH, Desmond RA, Stephens J, Hughes A, Hook EW, Stephens R, Hughes A, Hook III EW. 2002. Duration of persistence of gonococcal DNA detected by ligase chain reaction in men and women following recommended therapy for uncomplicated gonorrhea. J Clin Microbiol 40:3596–3601.

For peer review only - http://bmjopen.bmj.com/site/about/guidelines.xhtml

15. Carder C, Robinson AJ, Broughton C, Stephenson JM, Ridgway GL. 1999. Evaluation of self-taken samples for the presence of genital Chlamydia trachomatis infection in women using the ligase chain reaction assay. Int J STD AIDS 10:776–779.

16. Stewart CMWCMW, Schoeman SA, Booth RA, Smith SDSDD, Wilcox MHMHH, Wilson JDJDD. 2012. Assessment of self taken swabs versus clinician taken swab cultures for diagnosing gonorrhoea in women: single centre, diagnostic accuracy study. BMJ 345:e8107.

17. Samra Z, Rosenberg S, Madar-Shapiro L. 2011. Direct simultaneous detection of 6 sexually transmitted pathogens from clinical specimens by multiplex polymerase chain reaction and auto-capillary electrophoresis. Diagn Microbiol Infect Dis 70:17–21.

18. Chandeying V, Skov S, Farrell D, Lamlertkittikul S, Tabrizi S, Jarumanokul R. 2002. GYNAECOLOGY A Comparison of First-Void Urine , Self-Administered Low Vaginal Swab , Self-Inserted Tampon , and Endocervical Swab Using PCR Tests for the Detection of Infection with Chlamydia Trachomatis in Commercial Sex Workers and Women Attending Gyneco 14:201–209.

19. Sturm PDJ, Connolly C, Khan N, Ebrahim S, Sturm a W. 2004. Vaginal tampons as specimen collection device for the molecular diagnosis of non-ulcerative sexually transmitted infections in antenatal clinic attendees. Int J STD AIDS 15:94–8.

20. Upton A, Wilson J, Bissessor L. 2013. Introduction of routine polymerase chain reaction testing for Neisseria gonorrhoeae in a community laboratory. Sex Health. ELEC, CSIRO (P.O. Box 1139, Collingwood VIC 3066, Australia), A. Upton, Labtests, 27-41 Carbine Road, Mt Wellington 1060, New Zealand. E-mail: arlo.upton@labtests.co.nz.

21. Cheng A, Kirby JE. 2014. Evaluation of the hologic gen-probe PANTHER, APTIMA combo 2 assay in a tertiary care teaching hospital. Am J Clin Pathol 141:397–403.

22. Tanaka M, Nakayama H, Yoshida H, Takahashi K, Nagafuji T, Hagiwara T, Kumazawa J. 1998. Detection of Chlamydia trachomatis in vaginal specimens from female commercial sex workers using a new improved enzyme immunoassay. Sex Transm Infect 74:435–438.

23. Vahidnia A, Costa S, Veenings S, Tuin H, van Loon L, Bliekendaal H. 2014. Comparative evaluation of Roche Aurora FLOW, Becton and Dickinson Viper system, and Dynex DS2 for detection of Chlamydia trachomatis, Neisseria gonorrhoeae, and Trichomonas vaginalis in various clinical specimens. Diagn Microbiol Infect Dis 80:191–192.

24. Dietrich W, Rath M, Stanek G, Apfalter P, Huber JC, Tempfer C. 2010. Multiple site sampling does not increase the sensitivity of Chlamydia trachomatis detection in infertility patients. Fertil Steril 93:68–71.

25. Tanaka M, Nakayama H, Sagiyama K, Haraoka M, Yoshida H, Hagiwara T, Akazawa K, Naito S. 2000. Evaluation of a new amplified enzyme immunoassay (EIA) for the detection of Chlamydia trachomatis in male urine , female endocervical swab , and patient obtained vaginal swab specimens. J Clin Pathol 53:350–354.

26. Yu B, An Y, Xu G, Shan H. 2016. Detection of *Chlamydia trachomatis* and *Neisseria gonorrhoea* e based on cross-priming amplification. Lett Appl Microbiol 62:399–403.

27. Domeika M, Drulyte O. 2000. Use of PCR for the detection of genital Chlamydia trachomatis infection on self-obtained mailed vaginal samples. Acta Obstet Gynecol Scand 79:570–575.

28. Taylor SN, Van Der Pol B, Lillis R, Hook EW 3rd, Lebar W, Davis T, Fuller D, Mena L, Fine P, Gaydos CA, Martin DH. 2011. Clinical evaluation of the BD ProbeTecTM Chlamydia trachomatis Qx amplified DNA assay on the BD ViperTM system with XTRTM technology. Sex Transm Dis 38:603–609.

For peer review only - http://bmjopen.bmj.com/site/about/guidelines.xhtml

29. Zauli DAG, De Menezes CLP, De Oliveira CL. 2013. Development and padronization of three multiplex PCRs for the diagnosis of chlamydia trachomatis, toxoplasma gondii, herpes simplex viruses 1 and 2, and cytomegalovirus. Mol Biotechnol 54:1004–1009.

30. Domeika M, Bassiri M, Butrimiene I, Venalis a, Ranceva J, Vasjanova V. 1999. Evaluation of vaginal introital sampling as an alternative approach for the detection of genital Chlamydia trachomatis infection in women. Acta Obstet Gynecol Scand 78:131–136.

31. Thomas BJ, Pierpoint T, Taylor-Robinson D, Renton M. 1998. Sensitivity of the ligase chain reaction assay for detecting Chlamydia trachomatis in vaginal swabs from women who are infected at other sites. Sex Transm Infect 74:140–1.

32. Carroll KC, Aldeen WE, Morrison M, Anderson R, Lee D, Mottice S. 1998. Evaluation of the Abbott LCx ligase chain reaction assay for detection of Chlamydia trachomatis and Neisseria gonorrhoeae in urine and genital swab specimens from a sexually transmitted disease clinic population. J Clin Microbiol 36:1630–1633.

33. Falk L, Coble B-I, Mjörnberg P, Fredlund H. 2010. Sampling for Chlamydia trachomatis infection - a comparison of vaginal, first-catch urine, combined vaginal and first-catch urine and endocervical sampling. Int J STD AIDS 21:283–287.

34. Thomas BJ, Pierpoint T, Taylor-Robinson D, Renton M. 2001. Qualitative and quantitative aspects of the ligase chain reaction assay for Chlamydia trachomatis in genital tract samples and urines. Int J STD AIDS 12:589–594.

35. Lee GI, Yoen JP, Kang JS, Hwang SY, Hong YM, Yang JH, Yoon HK. 2013. A comparison of oligonucleotide-based microarray and real-time PCR for the detection of sexually transmitted infections. Biochip J 7:68–74.

36. Fang J, Husman C, DeSilva L, Chang R, Peralta L. 2008. Evaluation of self-collected vaginal swab, first void urine, and endocervical swab specimens for the detection of Chlamydia trachomatis and Neisseria gonorrhoeae in adolescent females. J Pediatr Adolesc Gynecol 21:355–360.

37. Van Der Pol B, Taylor SN, Lebar W, Davis T, Fuller D, Mena L, Fine P, Gaydos C a., Martin DH, Hook EW. 2012. Clinical Evaluation of the BD ProbeTec™ Neisseria gonorrhoeae Qx Amplified DNA Assay on the BD Viper™ System With XTR™ Technology. Sex Transm Dis 39:147–153.

38. Nuñez-Forero L, Moyano-Ariza L, Gaitán-Duarte H, Ángel-Müller E, Ruiz-Parra A, González P, Rodríguez A, Tolosa JE. 2016. Diagnostic accuracy of rapid tests for sexually transmitted infections in symptomatic women. Sex Transm Infect 92:24–28.

39. Fernández G, Martró E, González V, Saludes V, Bascuñana E, Marcó C, Rivaya B, López E, Coll P, Matas L, Ausina V. 2016. Usefulness of a novel multiplex real-time PCR assay for the diagnosis of sexually-transmitted infections. Enferm Infecc Microbiol Clin 34:471–6.

40. Garrow SC, Smith DW, Harnett GB. 2002. The diagnosis of chlamydia, gonorrhoea, and trichomonas infections by self obtained low vaginal swabs, in remote northern Australian clinical practice. Sex Transm Infect 78:278–281.

41. Van Der Pol B, Taylor SN, Liesenfeld O, Williams JA, Hook EW. 2013. Vaginal swabs are the optimal specimen for detection of genital Chlamydia trachomatis or Neisseria gonorrhoeae using the Cobas 4800 CT/NG test. Sex Transm Dis 40:247–50.

42. Gaydos CA, Van Der Pol B, Jett-Goheen M, Barnes M, Quinn N, Clark C, Daniel GE, Dixon PB, Hook EW 3rd, Group CS. 2013. Performance of the Cepheid CT/NG Xpert Rapid PCR Test for Detection of Chlamydia trachomatis and Neisseria gonorrhoeae. J Clin Microbiol 51:1666–1672.

For peer review only - http://bmjopen.bmj.com/site/about/guidelines.xhtml

43. Van Der Pol B, Hook 3rd EW, Williams JA, Smith B, Taylor SN. 2015. Performance of the BD CTQx and GCQx Amplified Assays on the BD Viper LT Compared With the BD Viper XTR System. Sex Transm Dis 42:521–523.

44. CHERNESKY MA, JANG DA. 2006. APTIMA transcription-mediated amplification assays for Chlamydia trachomatis and Neisseria gonorrhoeae. Expert Rev Mol Diagn 6:519–525.

45. Gokral JS, Mania-Pramanik J, Meherji PK, Mali BN. Introital swab testing for Chlamydia trachomatis in a resource-poor setting: an Indian perspective. Int J Fertil Womens Med 50:140–3.

46. Van Der Pol B, Williams JA, Fuller DA, Taylor SN, Hook EW. 2017. Combined testing for chlamydia, gonorrhea, and trichomonas by use of the BD max CT/GC/TV assay with genitourinary specimen types. J Clin Microbiol 55:155–164.

47. Fong H, Christian CW. 2012. Evaluating Sexually Transmitted Infections in Sexually Abused Children: New Techniques to Identify Old Infections. Clin Pediatr Emerg Med 13:202–212.

48. Gaydos C, Crotchfelt K, Shah N, Tennant M, Quinn TC, Gaydos JC, McKee KT, Rompalo AM. 2002. Evaluation of dry and wet transported intravaginal swabs in detection of Chlamydia trachomatis and Neisseria gonorrhoeae infections in female soldiers by PCR. J Clin Microbiol 40:758–761.

49. Gaydos CA. 2014. Review of use of a new rapid real-time PCR, the Cepheid GeneXpert ® (Xpert) CT/NG assay, for *Chlamydia trachomatis* and *Neisseria gonorrhoeae* : results for patients while in a clinical setting. Expert Rev Mol Diagn 14:135–137.

50. Hjelm E, Hallén a., Domeika M. 2001. Cervical, urine and vaginal specimens for detection of Chlamydia trachomatis by ligase chain reaction in women: A comparison. Acta Derm Venereol 81:285–288.

51. van Dommelen L, Dukers-Muijrers N, Van Tiel F, Brouwers E, Hoebe C. 2011. Evaluation of one-sample testing of self-obtained vaginal swabs and first-catch urine samples separately and in combination for the detection of Chlamydia trachomatis by two amplified DNA assays in women visiting a sexually transmitted disease clinic. Clin Microbiol Infect 17:S754–S755.

52. Meyer T, T. M. 2012. [Diagnosis and treatment of Chlamydia trachomatis infections]. Hautarzt 63:16–23.

53. Hsieh Y-H, Howell MR, Gaydos JC, Mckee KT, Quinn TC, Gaydos C a, MD MsPH, Mckee KT, Quinn TC, Gaydos C a. 2003. Preference among female Army recruits for use of self-administrated vaginal swabs or urine to screen for Chlamydia trachomatis genital infections. Sex Transm Dis 30:769–773.

54. Wiesenfeld HC, Heine RP, Rideout a., Macio I, DiBiasi F, Sweet RL. 1996. The vaginal introitus: A novel site for Chlamydia trachomatis testing in women. Am J Obstet Gynecol 174:1542–1546.

55. Sautter RL, LeBar WD, Greenwald E. 2009. The Laboratory's Role in Evaluating Sexually Transmitted Diseases as a Result of Sexual Abuse. Clin Microbiol Newsl 31:145–150.

56. Keane F, Bendall R, Saulsbury N, Haddon L. 2007. A comparison of self-taken vulvovaginal and cervical samples for the diagnosis of Chlamydia trachomatis infection by polymerase chain reaction. Int J STD AIDS 18:98–100.

57. Witkin SS, Inglis SR, Polaneczky M. 1996. Detection of Chlamydia trachomatis and Trichomonas vaginalis by polymerase chain reaction in introital specimens from pregnant women. Am J Obstet Gynecol 175:165–167.

For peer review only - http://bmjopen.bmj.com/site/about/guidelines.xhtml

58. Van Der Pol B. 2017. Profile of the triplex assay for detection of chlamydia, gonorrhea and trichomonas using the BD MAX^{TM} System. Expert Rev Mol Diagn 17:539–547.

59. Knox J, Tabrizi SN, Miller P, Petoumenos K, Law M, Chen S, Garland SM. 2002. Evaluation of self-collected samples in contrast to practitioner-collected samples for detection of Chlamydia trachomatis, Neisseria gonorrhoeae, and Trichomonas vaginalis by polymerase chain reaction among women living in remote areas. Sex Transm Dis 29:647–54.

60. Kellogg ND, Baillargeon J, Lukefahr JL, Lawless K, Menard SW. 2004. Comparison of nucleic acid amplification tests and culture techniques in the detection of Neisseria gonorrhoeae and Chlamydia trachomatis in victims of suspected child sexual abuse. J Pediatr Adolesc Gynecol 17:331–339.

61. Kriesel JD, Bhatia AS, Barrus C, Vaughn M, Gardner J, Crisp RJ. 2016. Multiplex PCR testing for nine different sexually transmitted infections. Int J STD AIDS 27:1275–1282.

62. Abou Tayoun AN, Burchard PR, Caliendo AM, Scherer A, Tsongalis GJ. 2015. A multiplex PCR assay for the simultaneous detection of Chlamydia trachomatis, Neisseria gonorrhoeae, and Trichomonas vaginalis. Exp Mol Pathol 98:214–218.

63. Li J, Jang D, Gilchrist J, Smieja M, Ewert R, MacRitchie C, Chernesky M. 2014. Comparison of flocked and Aptima swabs and two specimen transport media in the Aptima combo 2 assay. J Clin Microbiol 52:3808–3809.

64. Lawton B, Bromhead C. 2013. Nucleic acid amplification tests of self-taken vulvovaginal swabs are more sensitive than clinician taken endocervical culture for gonorrhoea. Evid Based Med 18:e46.

65. Lanjouw E, van Daele PLA, Raes MPE, van der Meijden WI. 2009. Consecutively Acquired Sexually Transmitted Infections Mimicking Crohn's Disease. Am J Gastroenterol 104:532–533.

66. Macmillan S, McKenzie H, Templeton A, Strandell A. 2003. Screening for chlamydial infection by DNA testing in urine and vulval swabs was highly sensitive and more acceptable than endocervical swabs. Evidence-based Obstet Gynecol 5:120–121.

67. Ostergaard LJ, Moller JK, Andersen B, Olesen F. 1997. Self-collected and mailed samples for diagnosis of urogenital Chlamydia trachomatis infection in women [Danish]. Ugeskr Laeger 159:5089–92.

68. McKechnie ML, Hillman RJ, Jones R, Lowe PC, Couldwell DL, Davies SC, King F, Kong F, Gilbert GL. 2011. The prevalence of urogenital micro-organisms detected by a multiplex PCR-reverse line blot assay in women attending three sexual health clinics in Sydney, Australia. J Med Microbiol 60:1010–1016.

69. Macmillan S, McKenzie H, Templeton A. 2003. Parallel observation of four methods for screening women under 25 years of age for genital infection with Chlamydia trachomatis. Eur J Obstet Gynecol Reprod Biol 107:68–73.

70. Cheng P, Tang J, Cheng H, Liu X, Wang Y. 2014. PCR methods for simultaneous detection of Neisseria agonorrhoeae, Chlamydiatrachomatis and Ureaplasma parvum. Chinese J Biol 27:109–114.

71. Ostergaard L, Møller JK. 1995. Use of PCR and direct immunofluorescence microscopy for confirmation of results obtained by Syva MicroTrak Chlamydia enzyme immunoassay. J Clin Microbiol 33:2620–3.

72. Ostergaard L, Møller JK, Andersen B, Olesen F. 1996. Diagnosis of urogenital Chlamydia trachomatis infection in women based on mailed samples obtained at home: multipractice comparative study. BMJ 313:1186–9.

73. Polaneczky M, Quigley C, Pollock L, Dulko D, Witkin SS. 1998. Use of self-collected

For peer review only - http://bmjopen.bmj.com/site/about/guidelines.xhtml

vaginal specimens for detection of Chlamydia trachomatis infection. Obstet Gynecol 91:375–8.

74. Renton A, Thomas BM, Gill S, Lowndes C, Taylor-Robinson D, Patterson K. 2006. Chlamydia trachomatis in cervical and vaginal swabs and urine specimens from women undergoing termination of pregnancy. Int J STD AIDS 17:443–447.

75. Koumans EH, Black CM, Markowitz LE, Unger ER, Pierce A, Sawyer MK, Papp JR. 2003. Comparison of Methods for Detection of Chlamydia trachomatis and Neisseria gonorrhoeae Using Commercially Available Nucleic Acid Amplification Tests and a Liquid Pap Smear Medium. Society 41:1507–1511.

76. Rompalo AM, Gaydos CA, Shah N, Tennant M, Crotchfelt KA, Madico G, Quinn TC, Daniel R, Shah K V, Gaydos JC, McKee KT. 2001. Evaluation of use of a single intravaginal swab to detect multiple sexually transmitted infections in active-duty military women. Clin Infect Dis 33:1455–1461.

77. Leslie DE, Azzato F, Ryan N, Fyfe J. 2003. An assessment of the Roche Amplicor ® gonorrhoeae multiplex PCR assay in routine diagnostic use on a variety of specimen types. Commun Dis Intell Q Rep 27:373–9.

78. Rumyantseva T, Golparian D, Nilsson CS, Johansson E, Falk M, Fredlund H, Van Dam A, Guschin A, Unemo M. 2015. Evaluation of the new AmpliSens multiplex real-time PCR assay for simultaneous detection of Neisseria gonorrhoeae, Chlamydia trachomatis, Mycoplasma genitalium, and Trichomonas vaginalis. Apmis 123:879–886.

79. Whiting PF, Rutjes AWS, Westwood ME, Mallett S, Deeks JJ, Reitsma JB, Leeflang MMG, Sterne JAC, Bossuyt PMM. 2011. QUADAS-2: a revised tool for the quality assessment of diagnostic accuracy studies. Ann Intern Med 155:529–36.

80. Chernesky MA, Jang D, Luinstra K, Chong S, Smieja M, Cai W, Hayhoe B, Portillo E, Main C, Ewert R, Macritchie C, Main C, Ewert R, Main MacRitchie C, Ewert R. 2006. High analytical sensitivity and low rates of inhibition may contribute to detection of Chlamydia trachomatis in significantly more women by the APTIMA Combo 2 assay. J Clin Microbiol 44:400–405.

81. Chernesky M, Jang D, Gilchrist J, Hatchette T, Poirier A, Flandin JF, Smieja M, Ratnam S. 2014. Head to Head Comparison of Second Generation Nucleic Acid Amplification Tests for Chlamydia trachomatis and Neisseria gonorrhoeae on Female Urines and Self-Collected Vaginal Swabs. J Clin Microbiol 52:2305–10.

82. Cosentino LA, Landers D V, Hillier SL. 2003. Detection of Chlamydia trachomatis and Neisseria gonorrhoeae by strand displacement amplification and relevance of the amplification control for use with vaginal swab specimens. J Clin Microbiol 41:3592–6.

83. Gaydos CA, Cartwright CP, Colaninno P, Welsch J, Holden J, Ho SY, Webb EM, Anderson C, Bertuzis R, Zhang L, Miller T, Leckie G, Abravaya K, Robinson J. 2010. Performance of the Abbott RealTime CT/NG for detection of Chlamydia trachomatis and Neisseria gonorrhoeae. J Clin Microbiol 48:3236–43.

84. Hook EW 3rd, Ching SF, Stephens J, Hardy KF, Smith KR, Lee HH. 1997. Diagnosis of Neisseria gonorrhoeae infections in women by using the ligase chain reaction on patient-obtained vaginal swabs. J Clin Microbiol 35:2129–2132.

85. Le Roy C, Le Hen I, Clerc M, Arfel V, Normandin F, Bebear C, de Barbeyrac B. 2012. The first performance report for the Bio-Rad Dx CT/NG/MG assay for simultaneous detection of Chlamydia trachomatis, Neisseria gonorrhoeae and Mycoplasma genitalium in urogenital samples. J Microbiol Methods 89:193–197.

86. Schachter J, Chernesky MA, Willis DE, Fine PM, Martin DH, Fuller D, Jordan JA, Janda W, Hook EW. 2005. Vaginal swabs are the specimens of choice when screening for Chlamydia trachomatis and Neisseria gonorrhoeae: results from a

For peer review only

multicenter evaluation of the APTIMA assays for both infections. Sex Transm Dis 32:725–8.

87. Shipitsyna E, Zolotoverkhaya E, Agne-Stadling I, Krysanova A, Savicheva A, Sokolovsky E, Domeika M, Unemo M. 2009. First evaluation of six nucleic acid amplification tests widely used in the diagnosis of Chlamydia trachomatis in Russia. J Eur Acad Dermatology Venereol 23:268–276.

88. Stary A, Schuh E, Kerschbaumer M, Götz B, Lee H. 1998. Performance of transcription-mediated amplification and ligase chain reaction assays for detection of chlamydial infection in urogenital samples obtained by invasive and noninvasive methods. J Clin Microbiol 36:2666–70.

For peer review only

For peer review only - http://bmjopen.bmj.com/site/about/guidelines.xhtml

PRISMA 2009 Checklist

Section/topic	#	Checklist item	Reported on page #
**TITLE**			
Title	1	Identify the report as a systematic review, meta-analysis, or both.	1
**ABSTRACT**			
Structured summary	2	Provide a structured summary including, as applicable: background; objectives; data sources; study eligibility criteria, participants, and interventions; study appraisal and synthesis methods; results; limitations; conclusions and implications of key findings; systematic review registration number.	2-3
**INTRODUCTION**			
Rationale	3	Describe the rationale for the review in the context of what is already known.	4-6
Objectives	4	Provide an explicit statement of questions being addressed with reference to participants, interventions, comparisons, outcomes, and study design (PICOS).	6-7
**METHODS**			
Protocol and registration	5	Indicate if a review protocol exists, if and where it can be accessed (e.g., Web address), and, if available, provide registration information including registration number.	6
Eligibility criteria	6	Specify study characteristics (e.g., PICOS, length of follow-up) and report characteristics (e.g., years considered, language, publication status) used as criteria for eligibility, giving rationale.	6-7
Information sources	7	Describe all information sources (e.g., databases with dates of coverage, contact with study authors to identify additional studies) in the search and date last searched.	7, Supplemental file
Search	8	Present full electronic search strategy for at least one database, including any limits used, such that it could be repeated.	Supplemental file
Study selection	9	State the process for selecting studies (i.e., screening, eligibility, included in systematic review, and, if applicable, included in the meta-analysis).	7-8
Data collection process	10	Describe method of data extraction from reports (e.g., piloted forms, independently, in duplicate) and any processes for obtaining and confirming data from investigators.	8
Data items	11	List and define all variables for which data were sought (e.g., PICOS, funding sources) and any assumptions and simplifications made.	8
Risk of bias in individual studies	12	Describe methods used for assessing risk of bias of individual studies (including specification of whether this was done at the study or outcome level), and how this information is to be used in any data synthesis.	8, Supplemental file
Summary measures	13	State the principal summary measures (e.g., risk ratio, difference in means).	8

For peer review only

PRISMA 2009 Checklist

Synthesis of results	14	Describe the methods of handling data and combining results of studies, if done, including measures of consistency (e.g., I^2) for each meta-analysis.	8

Page 1 of 2

Section/topic	#	Checklist item	Reported on page #
Risk of bias across studies	15	Specify any assessment of risk of bias that may affect the cumulative evidence (e.g., publication bias, selective reporting within studies).	8, Supplemental file
Additional analyses	16	Describe methods of additional analyses (e.g., sensitivity or subgroup analyses, meta-regression), if done, indicating which were pre-specified.	NA
**RESULTS**			
Study selection	17	Give numbers of studies screened, assessed for eligibility, and included in the review, with reasons for exclusions at each stage, ideally with a flow diagram.	8-9 Figure 1 Supplemental file
Study characteristics	18	For each study, present characteristics for which data were extracted (e.g., study size, PICOS, follow-up period) and provide the citations.	Tables 1 – 2 Supplemental file, Tables S3-4
Risk of bias within studies	19	Present data on risk of bias of each study and, if available, any outcome level assessment (see item 12).	Supplemental file, Table S2
Results of individual studies	20	For all outcomes considered (benefits or harms), present, for each study: (a) simple summary data for each intervention group (b) effect estimates and confidence intervals, ideally with a forest plot.	Figures 2-3
Synthesis of results	21	Present results of each meta-analysis done, including confidence intervals and measures of consistency.	NA
Risk of bias across studies	22	Present results of any assessment of risk of bias across studies (see Item 15).	14, Supplemental file
Additional analysis	23	Give results of additional analyses, if done (e.g., sensitivity or subgroup analyses, meta-regression [see Item 16]).	NA
**DISCUSSION**			
Summary of evidence	24	Summarize the main findings including the strength of evidence for each main outcome; consider their relevance to key groups (e.g., healthcare providers, users, and policy makers).	17-18

For peer review only

For peer review only - http://bmjopen.bmj.com/site/about/guidelines.xhtml

PRISMA 2009 Checklist

Limitations	25	Discuss limitations at study and outcome level (e.g., risk of bias), and at review-level (e.g., incomplete retrieval of identified research, reporting bias).	18
Conclusions	26	Provide a general interpretation of the results in the context of other evidence, and implications for future research.	19
**FUNDING**			
Funding	27	Describe sources of funding for the systematic review and other support (e.g., supply of data); role of funders for the systematic review.	20

From: Moher D, Liberati A, Tetzlaff J, Altman DG, The PRISMA Group (2009). Preferred Reporting Items for Systematic Reviews and Meta-Analyses: The PRISMA Statement. PLoS Med 6(7): e1000097. doi:10.1371/journal.pmed1000097

For more information, visit: www.prisma-statement.org.

For peer review only

For peer review only - http://bmjopen.bmj.com/site/about/guidelines.xhtml

The PRISMA for Abstracts Checklist

TITLE	CHECKLIST ITEM	REPORTED ON PAGE #
1. Title:	Identify the report as a systematic review, meta-analysis, or both.	1
**BACKGROUND**		
2. Objectives:	The research question including components such as participants, interventions, comparators, and outcomes.	2
**METHODS**		
3. Eligibility criteria:	Study and report characteristics used as criteria for inclusion.	2
4. Information sources:	Key databases searched and search dates.	2
5. Risk of bias:	Methods of assessing risk of bias.	2
**RESULTS**		
6. Included studies:	Number and type of included studies and participants and relevant characteristics of studies.	2
7. Synthesis of results:	Results for main outcomes (benefits and harms), preferably indicating the number of studies and participants for each. If meta-analysis was done, include summary measures and confidence intervals.	2
8. Description of the effect:	Direction of the effect (i.e. which group is favoured) and size of the effect in terms meaningful to clinicians and patients.	2
**DISCUSSION**		
9. Strengths and Limitations of evidence:	Brief summary of strengths and limitations of evidence (e.g. inconsistency, imprecision, indirectness, or risk of bias, other supporting or conflicting evidence)	2
10. Interpretation:	General interpretation of the results and important implications	2
**OTHER**		
11. Funding:	Primary source of funding for the review.	20
12. Registration:	Registration number and registry name.	NA

BMJ Open

Evaluation of the performance of nucleic acid amplification tests (NAATs) in detection of chlamydia and gonorrhoea infection in vaginal specimens relative to patient infection status: a systematic review

Journal:	*BMJ Open*
Manuscript ID	bmjopen-2018-022510.R2
Article Type:	Research
Date Submitted by the Author:	15-Oct-2018
Complete List of Authors:	Rönn, Minttu; Imperial College London, Department of Infectious Disease Epidemiology; Harvard T. H. Chan School of Public Health, Department of Global Health and Population<br>Mc Grath-Lone, Louise; Imperial College London, Department of Infectious Disease Epidemiology<br>Davies, Bethan; Imperial College London, Department of Infectious Disease Epidemiology<br>Wilson, Janet; Leeds General Infirmary,<br>Ward, Helen; Imperial College London, Department of Infectious Disease Epidemiology
**Primary Subject Heading**:	Infectious diseases
Secondary Subject Heading:	Sexual health, Diagnostics, Evidence based practice
Keywords:	chlamydia, gonorrhea, patient infection status, Molecular diagnostics < INFECTIOUS DISEASES

SCHOLARONE™
Manuscripts

Evaluation of the performance of nucleic acid amplification tests (NAATs) in detection of chlamydia and gonorrhoea infection in vaginal specimens relative to patient infection status: a systematic review

AUTHORS:

Minttu M. Rönn[1,2], Louise Mc Grath-Lone[1], Bethan Davies[1], Janet D. Wilson[3], Helen Ward[1]

1 Department of Infectious Disease Epidemiology, Imperial College London, London, UK
2. Department of Global Health and Population, Harvard T.H. Chan School of Public Health
3 Leeds Sexual Health, Leeds Teaching Hospitals NHS Trust, Leeds, UK

Corresponding Author:
Minttu M. Rönn
Imperial College London
St Mary's Campus
Norfolk Place
W2 1PG London

MMR: mronn@hsph.harvard.edu
LM: louise.mcgrath-lone@ed.ox.ac.uk
BD: bethan.davies06@imperial.ac.uk
JDW: janet-d.wilson@nhs.net
HW: h.ward@imperial.ac.uk

Abstract word count: 263
Manuscript word count: 3140
References: 33
Tables: 1 (in the manuscript)
Figures: 3 (attached)
Supplemental material: 1 (attached)

Keywords: chlamydia, gonorrhea, diagnostics, patient infection status

For peer review only - http://bmjopen.bmj.com/site/about/guidelines.xhtml

ABSTRACT

OBJECTIVE: We evaluated the performance of nucleic acid amplification tests (NAATs) using vaginal specimens in comparison to specimens from the cervix or urine in their ability to detect chlamydia and gonorrhea infection in women based on patient infection status (PIS).

DESIGN: Systematic review.

DATA SOURCES: EMBASE and Ovid MEDLINE databases were searched through 3 October 2017.

ELIGIBILITY CRITERIA FOR SELECTING STUDIES: We included studies that tested samples from the vagina and ≥1 other site (cervix and/or urine) with ≥2 NAATs for chlamydia and ≥2 NAATs or 1 NAAT and culture for gonorrhoea for each site.

DATA EXTRACTION AND SYNTHESIS: Performance is defined as the sensitivity of a NAAT using a specimen site and PIS of the patient. We assessed risk of bias using modified QUADAS-2.

RESULTS: Nine publications met the inclusion criteria (eight for chlamydia; six for gonorrhoea) and were narratively reviewed. Pooled summary estimates were not calculated due to the variable methodology and PIS definitions. Tests performed on vaginal specimens accomplished similar performance to cervical and urine specimens for chlamydia (range of performance estimates: vaginal 65-100%, cervical 59-97%, urine 57-100%) and gonorrhoea (vaginal 64-100%, cervical 85-100%, urine 67-94%). Vaginal specimens were estimated to have a performance >80% for chlamydia and gonorrhoea infections in all but one study.

For peer review only - http://bmjopen.bmj.com/site/about/guidelines.xhtml

CONCLUSIONS: Performance of the NAATs for chlamydia and gonorrhea detection using vaginal specimens was similar to that of cervical and urine specimens relative to PIS. As vaginal samples have a higher acceptability and lower cost, the study can support clinical testing guidelines by providing evidence that vaginal samples are a suitable alternative to traditionally used specimens.

ARTICLE SUMMARY

Strengths and limitations of this study

- The systematic review included studies which had used a conservative definition of patient infection status (PIS), which can improve estimates of performance.
- The study included a broad time frame and the search did not have language restrictions, which increased the scope of the literature search.
- The lack of standardised definitions of PIS and variable methodologies used restricted us to qualitative analyses.

For peer review only - http://bmjopen.bmj.com/site/about/guidelines.xhtml

INTRODUCTION

In the UK, practices for *Chlamydia trachomatis* (chlamydia) and *Neisseria gonorrhoea* (gonorrhoea) testing are not currently standardised. Nucleic acid amplification tests (NAATs) are recommended for the diagnosis of chlamydia and gonorrhoea due to their high sensitivity and specificity (1–3), but a variety of urogenital specimens (i.e. urine, cervical and vaginal) are used for testing at clinic level. The performance of NAATs to detect infection is known to vary by the type of specimen that is tested. A systematic review by Cook *et al.* (2005) estimated that the overall performance of NAATs for gonorrhoea was just 55.6% (95% CI: 36.3-74.9%) for urine samples compared to 94.2% (90.5-98.0%) for cervical samples, with a specificity of 98.7% (97.5-99.9%) and 99.2% (98.4-100%), respectively (4). Sensitivity is typically defined as the probability that a diagnostic test provides a positive result given that the the true test result – using gold standard – is positive, and a specificity is the probability that a diagnostic test yields a negative result given the true test result – using gold standard – is negative.

Recent studies have suggested that vaginal swabs may be better at detecting chlamydia and gonorrhoea than the traditionally-used urine and cervical samples (5–14). An issue related to these studies is that the estimates may be overestimated due to the use of discrepant analysis. As there is no gold-standard diagnostic test for either chlamydia or gonorrhoea (i.e. one with 100% sensitivity and specificity), studies evaluating the performance of a novel NAAT have tended to compare its performance against presumably poorer performing existing NAATs. Given the assumption that the novel NAAT has a higher sensitivity, it would be reasonable to expect that it would yield a

For peer review only - http://bmjopen.bmj.com/site/about/guidelines.xhtml

98 positive result in some cases where the existing NAAT yields a negative result. The
99 discrepant results create a challenge in differentiating false positive results from true
100 positive results for the novel NAAT. In this situation, a third test is often performed. If this
101 third test is positive then the positive result from the novel NAAT is defined a "true positive"
102 and if the third test is negative the result from the novel NAAT is defined a "false positive".
103 This approach is called discrepant analysis (DA). DA has been criticized because it only
104 retests "false positives" and does not seek to improve the classification of "false
105 negatives" which potentially introduces a bias towards overestimating the performance of
106 the novel NAAT (15).
107
108 To address some of the potential bias from DA, patient infection status (PIS) was
109 developed to represent an "extended gold-standard" by measuring test performance
110 based on information from multiple sources. The performance of PIS can be thought of
111 as the sensitivity of a NAAT from a given site given the patient is considered to have an
112 infection (determined by the sites and definition used for PIS). There is no consensus
113 definition for PIS, but in general, it involves performing ≥1 test(s) at >1 site and interpreting
114 the results using an algorithm that describes the number of positive tests (by test type or
115 site) needed to define a person as infected. A limitation of the current evidence base is
116 that many studies that have concluded in favour of using vaginal samples over cervical
117 or urine for the diagnosis of chlamydia and gonorrhoea have used just one NAAT per test
118 site (6, 9–14). Ideally, PIS is determined with the results of multiple NAATs adjusting for
119 potential problems with the NAATs as well as for the possibility that a person might be
120 infected at one or more anatomical sites (16). PIS is not a perfect reference-standard,

For peer review only - http://bmjopen.bmj.com/site/about/guidelines.xhtml

and it has received criticism for similar reasons as discrepant analysis whereby there is a conditional dependence in the estimation of test performance and the PIS (17). This may result in overestimation of the sensitivity of a test from a given site, but by including multiple sites in the PIS it can also result in lower site-specific performance. PIS allows us to compare different sites within a study and similar PIS definition allow for comparison between studies. The ultimate use depends on the best combination of specimen and test. In most countries, health funding restraints mean it is only possible to use one specimen for chlamydia and gonorrhoea testing in women, and therefore it is important to know which sites to sample to diagnose the highest proportion of infections.

The overall comparison of vaginal test performance compared to cervical and urine tests warrants further evaluation to inform evidence-based clinical testing guidelines. To this end, we performed a systematic review of the published literature. The primary aim of this review was to compare the performance of specimens taken from the vagina to those from the cervix or urine in diagnosing chlamydia and gonorrhoea infection in women based on a PIS definition. Given the lack of calibrated gold-standard diagnostic tests and variation in the definition of PIS in the literature, we purposively used conservative selection criteria to identify studies that applied rigorous testing criteria in the process of defining PIS. We also examined the quality of the studies in light of the known biases in the PIS methodology to increase our ability to assess the evidence-base.

METHODS

For peer review only - http://bmjopen.bmj.com/site/about/guidelines.xhtml

We performed a systematic review following the Preferred Reporting Items for Systematic Reviews and Meta-Analyses (PRISMA) guidelines (18). The protocol for our review was not registered in advance. We included studies which (a) tested for urogenital chlamydia and/or gonorrhoea infection in (b) post-pubertal women (c) using a vaginal specimen (collected by any method) and (d) at least one other urine or cervical specimen. We limited this review to studies that used a conservative approach for defining PIS whereby each specimen site had to be tested with (e) ≥2 NAATs for chlamydia and ≥2 NAATs or 1 NAAT plus culture for gonorrhoea (as culture is an accepted alternative to NAAT for gonorrhoea diagnosis (19)). Finally, studies had to (f) report the performance of vaginal specimens compared to the PIS, or give the numbers of patients infected and results from the tests so that this could be calculated.

NAATs were defined as commercially available or in-house tests involving DNA or RNA replication using polymerase chain reaction (PCR), transcription mediated amplification (TMA), ligase chain reaction (LCR) or strand displacement amplification (SDA). Studies that used pooled samples or novel point-of-care tests were excluded together with studies that used one NAAT on all samples and only confirmed positive or equivocal results with a second NAAT. No restrictions were set on year or language of publication. However, only peer-reviewed studies were included; conference abstracts/proceedings, notes, letters and book chapters were excluded.

The electronic databases EMBASE (1947 to 2017) and Ovid MEDLINE (1946 to 2017) were searched on 3 October 2017 by two authors, using OvidSP as the search platform

For peer review only - http://bmjopen.bmj.com/site/about/guidelines.xhtml

For peer review only

(MMR, LM or BD). The search strategy included keywords related to the infections, interventions, specimens of interest and outcomes. The full search strategy is presented in the supplement (File S1).

After duplicate references had been removed by OvidSP, two authors (MMR LM, or BD) independently selected studies by scanning titles and abstracts. A list of potentially eligible publications was obtained through consensus agreement and full texts were obtained online, through the British Library or directly from the authors. The full texts were then reviewed for inclusion and a third author was consulted if there was disagreement between the two reviewers. Where the language of the article was not English we consulted colleagues fluent in the relevant language. The reference lists of all studies meeting the inclusion criteria, as well as recent key papers and systematic reviews identified during the search process, were examined iteratively for additional references.

One author extracted information from the selected studies using a data extraction form in Microsoft Excel. The extracted data was checked against the studies by a second author. Two reviewers independently assessed the risk of bias in patient selection, PIS definition and flow and timing (of different tests and analysis, including here also discrepant analysis) for each included study using an adapted version of the quality assessment of diagnostic accuracy studies (QUADAS-2) tool (20). The purpose of this quality assessment was to consider the potential biases and variation within the individual studies that may account for heterogeneity across the studies. For each study, we calculated or reported the sensitivity of each NAAT for each sample site, and calculated

For peer review only

For peer review only - http://bmjopen.bmj.com/site/about/guidelines.xhtml

confidence intervals using the exact binomial distribution. If the study did not present raw numbers, the summary estimate was extracted instead. Performance was defined as the "number of tests appearing to be a true positive at a site for a given NAAT / number of infected patients as determined by PIS". These performance measures were used to compare the ability of each sample site to accurately predict PIS by describing the overall differences within and between sample sites for chlamydia and gonorrhoea. Analyses and forest plots were done in R (21).

Patient and Public Involvement

Vaginal specimens are an acceptable and sometimes a preferred way for chlamydia and gonorrhea testing among women (22). In this study, we compare the evidence-base of performance of different testing sites for detection of chlamydia and gonorrhea by conducting a systematic review of published primary research. We did not involve patient groups in the design or conduct of the study. The findings of the study can be used to standardize testing practices.

RESULTS

The search on 3 October 2017 identified 567 studies of which 387 remained after de-duplication by the search platform OvidSP. We excluded 299 studies based on their abstracts and screened the full text of the remaining 89 studies of which one was identified through searching reference lists. We excluded 81 of these studies and the reasons for exclusion are presented in the supplement (File S1, Table S1). We identified one additional study through searching the reference lists (23) so that in total, nine eligible

For peer review only

For peer review only - http://bmjopen.bmj.com/site/about/guidelines.xhtml

studies were included in this review: eight for chlamydia (24–31) and six for gonorrhoea (23, 25, 28, 30, 31). A flowchart describing study selection is presented in Figure 1 and a description of the included studies is presented in Table 1. Few studies included results for patient characteristics or sample collection: three studies included self-collected vaginal swabs (23, 30, 31) and just one reported results for asymptomatic and symptomatic patients separately (25).

Figure 1. Flow chart of study selection.

For peer review only

Table 1. Characteristics of included studies

**Author and year (Reference) // Included infections**	**Review outcome the main focus of the study? // Aim of the study**	**Tests performed, (acronym for the NAAT)**	**Specimens/sites (in women) // Comments**	**PIS definition // Comments on discrepant analysis**
**Chernesky M. et al. (2006)** (27)<br><br>CT	No<br><br>To compare detection thresholds and inhibitor and infection rates from different specimens	APTIMA Combo 2, (TMA AC2)<br><br>ProbeTec ET, (SDA ProbeTec ET)<br><br>Amplicor, (PCR AMP)	3 EC, 3 CCVS and FCU divided into 3 aliquots<br><br>Each sample was tested as spiked (with added CT) or without added CT; seemingly all the tests were done to all the samples	≥1 site positive by ≥2 different tests or two specimens positive in a single test (≥2 out of 9 specimen-tests done); VS included in PIS<br><br>No discrepant analysis described
**Cherneskey M et al. (2014)** (31)<br><br>CT & NG	No<br><br>To compare the performance of four second-generation NAATs with FCU and VS	APTIMA Combo 2, (TMA AC2)<br><br>RealT*ime* CT/NG, (PCR RealT*ime*)<br><br>ProbeTec CT/GC Q^x, (SDA ProbeTec CT/GC Q^x)<br><br>Cobas CT/NG, (PCR Cobas CT/NG)	1 FCU and 4 SCVS<br><br>Each sample was tested as spiked (with added CT) or without added CT; all the tests were done to all the samples	≥2 of the 4 assays were positive for any specimen type; VS included in PIS<br><br>No discrepant analysis described
**Cosentino L.A. et al. (2003)** (28)<br><br>**CT & NG**	Yes<br><br>To compare vaginal swab specimens to endocervical swab specimens for the detection of CT and NG	ProbeTec ET, (SDA ProbeTec ET)<br><br>Amplicor, (PCR AMP)<br><br>Thayer-Martin medium and chocolate agar (for NG culture)	3 EC, 2 (presumably clinician-obtained) VS. One EC used for NG culture<br><br>CT was tested by PCR and SDA; NG (EC) was tested for by	Positive result by two different molecular tests for CT or for NG by culture or by two molecular tests; VS included in PIS<br><br>Discrepant analysis for discordant results by LCR

For peer review only - http://bmjopen.bmj.com/site/about/guidelines.xhtml

		LCx, (LCR) for discrepant results	culture and SDA	
**Gaydos C.A. et al. (2010)** (25)<br><br>CT & NG	No<br><br>To compare the performance of the new RealTime CT/NG assay with the Aptima Combo 2 assay	RealTime CT/NG, (PCR RealTime) – index<br><br>APTIMA Combo 2, (TMA AC2) – reference<br><br>ProbeTec ET, (SDA ProbeTec ET) – reference<br><br>NG culture	4 EC, 1 SCVS, 2 CCVS, 3 urine<br><br>Only one NAAT performed on the SCVS (this test was not used to define PIS); results for this are not presented	≥1 positive result by both of the 2 reference NAATs, additionally for NG if culture positive the subject was defined as infected. Infection absent if ≥1 reference NAAT was negative for all sample types<br><br>Discrepant analysis: for CT retested discordant results, for NG not done
**Hook, E et al. (1997)** (23)<br><br>NG	Yes<br><br>To evaluate patient-obtained vaginal specimens tested with culture and LCR assays for NG compared to clinician-collected specimens	LCx, (LCR)<br><br>Modified Thayer-Martin medium (for NG culture)	3 SCVS, 3 EC (one sample at each site not part of this study as processed for CT)	Culture positive from either site; or LCR positive and culture negative with a positive confirmatory LCR; VS is included in PIS<br><br>Discrepant analysis with, alternative TMA with different target site to confirm discordant results
**Le Roy C. et al. (2012)** (24)<br><br>CT & NG[a]	No, data extracted based on their reporting<br><br>Determine clinical performance of Bio-Rad CT/NG/MG assay for detection of CT, NG and mycoplasma genitalium	Dx CT/NG/MG Assay, (qPCR) – index test<br><br>Cobas TaqMan CT, (qPCR TaqMan) - reference<br><br>NG culture	Symptomatic: 2 SCVS, 2 EC and 2 FCU. Asymptomatic: 2 SCVS and FCU.<br><br>More tests done on symptomatic patients, but all samples seem to have been treated the same.	Study definition: At least 2 positive results from either of the two assays. We determined PIS based on test results for FCU and VS (which were available for all patients, see supplement material for further information); all infected patients had ≥2 positive tests and a positive test at both sites; VS is included in PIS<br><br>Discrepant analysis used for discordant results
**Schachter J et al. (2005)** (30)	Yes<br><br>To evaluate the performance of	APTIMA CT, (TMA ACT)<br><br>APTIMA GC, (TMA AGC)	1 FCU, 1 SCVS, 1 CCVS, 2 EC swabs<br><br>All samples tested with	Infected if either BD or AC2 were positive on FCU or EC . VS not included in PIS

For peer review only - http://bmjopen.bmj.com/site/about/guidelines.xhtml

**CT & NG**	APTIMA assays on vaginal swabs for CT and NG	APTIMA Combo 2, (TMA AC2)<br><br>ProbeTec ET, (SDA ProbeTec ET)	three TMAs (2 for CT and 2 for NG) FCU and 1x EC swab were also tested with ProbeTec ET)	No discrepant analysis described
**Shipitsyna E. et al. (2008)** (26)<br><br>CT	No<br><br>To evaluate the performance of five PCRs and a recently introduced nucleic acid sequence-based amplification (NASBA) assay	Different 'in-house' PCRs tested: cPCR-DT, rtPCR-DT, cPCR-Ly, cPCR-Ep, rtPCR-Ep, real-time NASBA assay<br><br>Reference methods: Amplicor, (PCR AMP)<br><br>LightMix, (PCR Lightmix)	Used the subsample who had 4 specimens collected each: 2 EC and 2 VS<br><br>All sample sites tested using reference NAAT and at least 3 other NAATs	Several (range) Russian PCRs used on the sample and the sample was considered true positive if a positive result by a Russian PCR was confirmed by the reference tests; VS included in PIS<br><br>Discrepant analysis for discordant results
**Stary A et al. (1998)** (29)<br><br>**CT**	No<br><br>To compare TMA assay with LCR assay in detection of CT in genital tract with different specimen types	Aptima CT, (TMA ACT)<br><br>LCx, (LCR)<br><br>McCoy cell culture<br><br>Direct-fluorescent antibody assay (DFA) or alternative TMA for confirming discrepant results	3 EC, 3 CCVS, FCU<br><br>EC and VS tested with LCR, TMA and culture. FCU tested with LCR and TMA	Positive culture at any site or positive result by both NAATs in one site, or one positive NAAT confirmed with discrepant analysis; VS included in PIS.<br><br>Discrepant analysis with DFA or TMA with different target site to confirm discordant results

a. Too few infections with gonorrhoea for analysis

CT: *Chlamydia trachomatis*; NG: *Neisseria gonorrhoea*

For peer review only

CCVS: Clinician-collected vaginal specimens; SCVS: Self-collected vaginal specimens; EC: endocervical swabs; FCU: first catch urine; VS: vaginal specimen

Tests used: LCR (Ligase Chain Reaction); PCR AMP (Polymerase Chain Reaction Amplicor); PCR Cobas CT/NG (Polymerase Chain Reaction Cobas CT/NG); PCR 'In house'; PCR RealTi*me* (Polymerase Chain Reaction RealTi*me*); qPCR TaqMan (Quantitative Polymerase Chain Reaction TaqMan); qPCR DxCT/NG/MG (Quantitative Polymerase Chain Reaction DxCT/NG/MG); PCR LightMix, (Polymerase Chain Reaction Lightmix); NASBA (Nucleic acid sequence-based amplification); SDA ProbeTec ET (Strand Displacement Amplification ProbeTec ET); SDA ProbeTec CT/GC Q^x, (Strand Displacement Amplification ProbeTec CT/GC Q^x); TMA AC2 (Transcription Mediated Amplification Aptima Combo-2); TMA ACT (Transcription Mediated Amplification Aptima CT); TMA AGC (Transcription Mediated Amplification Aptima GC)

Discrepant analysis: We define discrepant analysis to have occurred in situations where sample was positive for only one of the tests used. In these instances another test was done.

For peer review only

For peer review only - http://bmjopen.bmj.com/site/about/guidelines.xhtml

236 The factors considered in the modified QUADAS-2 assessment and the results are presented in the supplement (File S1, Table S2). All studies were rated as high or unclear risk for at least two of the five criteria related to risk of bias and applicability concerns; for example, risk for patient selection bias was either unclear (6/9) or high (3/9). Our inclusion criteria required there to be ≥2 NAATs for chlamydia and ≥2 NAATs or 1 NAAT plus culture for gonorrhoea performed at each anatomical site, but variation remained in the number and type of tests used in the PIS algorithms. The definitions included "≥2 positive tests out of a number of tests performed (regardless of the site)", "≥2 different tests positive at the same site", "any test positive in ≥2 different sites" and "≥2 different tests positive" (described in Table 1). In all but one (30), the risk of bias in the definition of PIS was considered high because the algorithm used was unclearly defined and/or included vaginal specimens (as the component whose performance is being evaluated should be independent of the gold-standard it is measured against).

Seven out of nine studies (with the exception of Chernesky *et al.* 2006 (27) and Chernesky *et al.* 2014 (31)) had performed a different number of tests on a subsample of their study population where most of the studies (6/9) performed discrepant analysis for discordant results (for example, for sites where only one NAAT of many was positive). Due to the low number of studies identified, and their variable methods and definitions of PIS, we deemed pooling of the results to be inappropriate. Instead, we compared the ability of each sample site to predict PIS using forest plots of site-specific performance from each study by NAAT used.

For peer review only - http://bmjopen.bmj.com/site/about/guidelines.xhtml

Chlamydia

Of the eight studies that considered chlamydia test performance (24–31), two directly addressed the question in this review (28, 30) and the remainder aimed to test the sensitivity of a novel NAAT in a clinical setting (24–27, 29, 31). All eight studies were performed in high-income countries. Where a broader description of the population sample was reported, the studies had recruited patients from sexual health or reproductive health clinics, youth centres or primary care settings. One study specified that the sample population was high-risk for acquisition of sexually transmitted infections (29).

The reported chlamydia prevalence in the included studies ranged from 8.1% to 23.2% based on varying definitions of PIS. The number of infected women according to PIS ranged from 19 to 69 (with one study giving a point estimate without the raw data (30)). The site-specific performance estimates and their confidence intervals are presented in Figure 2 and the results are presented in table format in the supplement (File S1, Table S3). Across the studies vaginal specimens performed similarly to cervical and urine specimens within a study, which can be observed by comparing results by study in Figure 2; performance range: vaginal 65-100%, cervical 59-97%, urine 57-100%). The lowest overall performance estimates were reported by Chernesky *et al.* (2006) using a first generation, less sensitive, NAAT (27).

For peer review only - http://bmjopen.bmj.com/site/about/guidelines.xhtml

Figure 2. Performance estimates for chlamydia by showing the sensitivity of the NAAT on a given specimen site relative to PIS. The studies are referenced by the first author, and publication year, and colors are by study for clarity.

Figure legend:

Each study is represented by a colour and where results are stratified by test, sampling or symptom status, this has been included in the figure.

Tests used: LCR (Ligase Chain Reaction); PCR AMP (Polymerase Chain Reaction Ampicor); PCR Cobas CT/NG (Polymerase Chain Reaction Cobas CT/NG); PCR 'In house'; PCR RealTi*me* (Polymerase Chain Reaction RealTi*me*); qPCR TaqMan (Quantitative Polymerase Chain Reaction TaqMan); qPCR DxCT/NG/MG (Quantitative Polymerase Chain Reaction DxCT/NG/MG); PCR LightMix, (Polymerase Chain Reaction Lightmix); NASBA (Nucleic acid sequence-based amplification); SDA ProbeTec ET (Strand Displacement Amplification Probetec ET); SDA ProbeTec CT/GC Q^x, (Strand Displacement Amplification ProbeTec CT/GC Q^x); TMA AC2 (Transcription Mediated Amplification Aptima Combo-2); TMA ACT (Transcription Mediated Amplification Aptima CT); TMA AGC (Transcription Mediated Amplification Aptima GC.

Gonorrhoea

We identified six studies that tested for gonorrhoea (23–25, 28, 30, 31). Of these, five had a sufficient number of infected women according to PIS for performance to be calculated and were included in this analysis (23, 25, 28, 30, 31). The excluded study found two gonorrhoea cases in 193 tested individuals and these were not analysed further (24). Of the five remaining studies, three addressed the question in this review (23, 28, 30) whilst

For peer review only

the remaining two compared the performance of a new assay (25, 31). As with chlamydia, all of the studies were performed in high-income countries.

The prevalence of gonorrhoea ranged from 2% to 16.8% in the five studies, based on PIS, as defined by the studies; the denominator used for performance estimates varied from 11 to 52, with one study only reporting point estimates (30). Figure 3 presents the forest plot and the results are presented in table format in the supplement (File S1, Table S4). The performance estimates at each site in the four studies were similar within each study: performance range for vaginal samples was 63.6-100%, for cervical samples 85-100% and for urine samples 66.7-94%. Three studies (23, 25, 28) included performance estimates for cervical swabs and two studies had performance estimates for urine (25, 31). The lowest overall performance estimates were reported by Chernesky *et al.* (2014) (31).

Figure 3. Performance estimates for gonorrhea showing the sensitivity of the NAAT on a given specimen site relative to PIS. The studies are referenced by the first author, and publication year, and colors are by study for clarity.

Figure legend:

Each study is represented by a colour and where results are stratified by test, sampling or symptom status, this has been included in the figure.

Tests used: LCR (Ligase Chain Reaction); PCR AMP (Polymerase Chain Reaction Ampicor); PCR Cobas CT/NG (Polymerase Chain Reaction Cobas CT/NG); PCR 'In house'; PCR RealTi*me* (Polymerase Chain Reaction RealTi*me*); qPCR TaqMan (Quantitative Polymerase Chain Reaction TaqMan); qPCR DxCT/NG/MG (Quantitative Polymerase Chain Reaction

For peer review only - http://bmjopen.bmj.com/site/about/guidelines.xhtml

DxCT/NG/MG); PCR LightMix, (Polymerase Chain Reaction Lightmix); NASBA (Nucleic acid sequence-based amplification); SDA ProbeTec ET (Strand Displacement Amplification Probetec ET); SDA ProbeTec CT/GC Q^x, (Strand Displacement Amplification ProbeTec CT/GC Q^x); TMA AC2 (Transcription Mediated Amplification Aptima Combo-2); TMA ACT (Transcription Mediated Amplification Aptima CT); TMA AGC (Transcription Mediated Amplification Aptima GC.

DISCUSSION

We conducted a systematic review to evaluate the evidence for the relative performance of vaginal specimens in the detection of chlamydia and gonorrhoea in women using studies with conservative definitions of PIS. In the studies included in this review, we found similar sensitivities between urine, cervical and vaginal specimen types. This finding is contrary to other studies which have found that vaginal specimens have a higher performance than cervical swabs (5, 7, 9, 32). This difference may be due to the less conservative definition of PIS used in other studies, which may be more prone for overestimation. Nevertheless, our study suggests that vaginal swabs are at least as good as urine and cervical swabs in detecting chalmydia and gonorrhea.

This study followed PRISMA guidelines for systematic reviews and assessed the quality of the included studies using a modified version of QUADAS-2. The searches were conducted systematically and all potentially eligible abstracts were screened by at least two authors. The papers included in this review represent consensus opinion following group discussion among the authors. There were no language or time restrictions and multiple databases were searched. Therefore, this review represents a robust and comprehensive summary of extant evidence on the comparative performance of vaginal

For peer review only

For peer review only - http://bmjopen.bmj.com/site/about/guidelines.xhtml

specimens in detecting chlamydia and gonorrhoea infection. A further strength of this study is that we excluded studies where only one test was performed at the vaginal site, even if ≥2 NAATs were used at all other sites (6, 10, 33, 34). By limiting the analysis to studies that performed ≥2 tests at multiple sites, we aimed to minimize the potential for overestimation of test performance (16). The QUADAS-2 assessment found the studies were of variable quality and had methodological limitations and heterogeneity, particularly in PIS definition which was an important element of other analysis. As a result, some bias is likely to remain in the estimates reported in this review and the performance estimates should not be directly compared across studies. We did not pool the results, and considered the studies to be highly variable, which makes the study more exploratory in nature.

Overall, we found that vaginal specimens performed similarly to cervical and urine specimens for the diagnosis of both chlamydia and gonorrhoea in the identified studies. Based on the available evidence, and given the acceptability (22) and lower cost of vaginal samples (8), our study further supports that vaginal samples provide an appropriate alternative to traditional cervical or urine testing methods. However, this review has also highlighted that there are a lack of studies that have attempted to explicitly answer this research question whilst taking into account the inherent biases in estimating diagnostic test performance, particularly for NAATs. Further primary research with appropriate robust methodology for determining test performance in symptomatic and asymptomatic patients is needed for a more authoritative comparison of the performance of different urogenital sample sites. We suggest that research should be directed towards

For peer review only - http://bmjopen.bmj.com/site/about/guidelines.xhtml

applying more sophisticated statistical methods for comparing test performance such as latent class models (17), which uses a weighted statistical model acknowledging the conditional dependence of the tests.

PATIENT CONSENT

Not required.

DATA SHARING STATEMENT

All data presented in this study is available in the manuscript or in the supplementary material.

FUNDING

MMR received funding from the Wellcome Trust (G090285/Z/09/Z) at the beginning of this study. BD has been funded by an MRC Population Health Scientist Fellowship (G0902120). HW, BD and LM were supported in part by the National Institute for Health Research (NIHR) Biomedical Research Centre based at Imperial College Healthcare NHS Trust and Imperial College London. HW and JW have received funding from the NIHR Research for Patient Benefit (RfPB) Programme. The funders had no role in study design, data collection and analysis, decision to publish, or preparation of the manuscript.

CONFLICT OF INTEREST

For peer review only

For peer review only - http://bmjopen.bmj.com/site/about/guidelines.xhtml

JW has received research funding in the form of extra diagnostic reagents and equipment for one study from Hologic/Gen-Probe and has received one lecture fee from BD Diagnostics.

AUTHOR CONTRIBUTIONS

HW and JW conceived the study idea. MMR and LM conducted the first review and BD and MMR conducted the update of the review with discrepancies solved by all three. MMR drafted the manuscript, which was subsequently edited by LM, BD, HW and JW. MMR prepared the tables and figures presented in the manuscript. All authors have read and accepted the final manuscript.

ACKNOWLEDGMENTS

The authors would like to thank Dr Mikaela Smit and Dr Annick Borquez for their help in evaluating and translating articles not available in English.

PRESENTATION AT A MEETING

Poster presented at 3rd Joint Conference of the British HIV Association, BHIVA with the British Association for Sexual Health and HIV, BASHH. Liverpool United Kingdom. Abstract: HIV Medicine 15 (pp 109-110), 2014.

For peer review only

For peer review only - http://bmjopen.bmj.com/site/about/guidelines.xhtml

REFERENCES

1. British Association for Sexual Health and HIV. 2015. Chlamydia trachomatis UK Testing Guidelines 1–17.
2. Bignell C, Fitzgerald M. 2011. UK national guideline for the management of gonorrhoea in adults, 2011. Int J STD AIDS 22:541–7.
3. Papp J, Schachter J, Gaydos C, Van Der Pol B. 2014. Recommendations for the laboratory-based detection of Chlamydia trachomatis and Neisseria gonorrhoeae--2014. MMWR Recomm Rep 63:1–19.
4. Cook RL, Hutchison SL, Østergaard L, Braithwaite RS, Ness RB. 2005. Systematic review: noninvasive testing for Chlamydia trachomatis and Neisseria gonorrhoeae. Ann Intern Med 142:914–25.
5. Schoeman SA, Stewart CMW, Booth RA, Smith SD, Wilcox MH, Wilson JD. 2012. Assessment of best single sample for finding chlamydia in women with and without symptoms: a diagnostic test study. BMJ 345:e8013.
6. Stewart CMWCMW, Schoeman SA, Booth RA, Smith SDSDD, Wilcox MHMHH, Wilson JDJDD. 2012. Assessment of self taken swabs versus clinician taken swab cultures for diagnosing gonorrhoea in women: single centre, diagnostic accuracy study. BMJ 345:e8107.
7. Shafer M, Moncada J, Boyer CB, Betsinger K, Flinn SD, Schachter J. 2003. Comparing first-void urine specimens, self-collected vaginal swabs, and endocervical specimens to detect Chlamydia trachomatis and Neisseria gonorrhoeae by a nucleic acid amplification test. J Clin Microbiol 41:4395–9.
8. Blake DR, Maldeis N, Barnes MR, Hardick A, Quinn TC, Gaydos CA. 2008. Cost-effectiveness of screening strategies for Chlamydia trachomatis using cervical swabs, urine, and self-obtained vaginal swabs in a sexually transmitted disease clinic setting. Sex Transm Dis 35:649–55.
9. Taylor SN, Van Der Pol B, Lillis R, Hook EW 3rd, Lebar W, Davis T, Fuller D, Mena L, Fine P, Gaydos CA, Martin DH. 2011. Clinical evaluation of the BD ProbeTecTM Chlamydia trachomatis Qx amplified DNA assay on the BD ViperTM system with XTRTM technology. Sex Transm Dis 38:603–609.
10. Van Der Pol B, Taylor SN, Liesenfeld O, Williams JA, Hook EW. 2013. Vaginal swabs are the optimal specimen for detection of genital Chlamydia trachomatis or Neisseria gonorrhoeae using the Cobas 4800 CT/NG test. Sex Transm Dis 40:247–50.
11. Falk L, Coble B-I, Mjörnberg P, Fredlund H. 2010. Sampling for Chlamydia trachomatis infection - a comparison of vaginal, first-catch urine, combined vaginal and first-catch urine and endocervical sampling. Int J STD AIDS 21:283–287.
12. Gaydos CA, Van Der Pol BB, Jett-Goheen M, Barnes M, Quinn N, Clark C, Daniel GE, Dixon PB, Hook EW 3rd, Group CS, Davis T, Pancholi P, Kerndt P, Horner P, Marrazzo J, De Burgh-Thomas A, Castro J, Ferguson D, Meyer M, Philip S, Van Der Pol BB, Thompson M, Taylor S, Ronk D, Fine P, Bachman L, Mayne D, Dietz C, Toevs K, Gettings N, Gall S, Patel R, Ross J, Brooks L, Hazan L, Clarke P, Groom R, Ackerman R, Anduczyk E, Mathews A, Blank S, Harris L, Tidings A, Moore P, Alexander S, Spurrell T, Group CS. 2013. Performance of the Cepheid CT/NG Xpert Rapid PCR Test for Detection of Chlamydia trachomatis and Neisseria gonorrhoeae. J Clin Microbiol 51:1666–1672.
13. Rompalo AM, Gaydos CA, Shah N, Tennant M, Crotchfelt KA, Madico G, Quinn TC, Daniel R,

For peer review only - http://bmjopen.bmj.com/site/about/guidelines.xhtml

Shah K V, Gaydos JC, McKee KT. 2001. Evaluation of use of a single intravaginal swab to detect multiple sexually transmitted infections in active-duty military women. Clin Infect Dis 33:1455–1461.

14. van Dommelen L, Dukers-Muijrers N, Van Tiel F, Brouwers E, Hoebe C. 2011. Evaluation of one-sample testing of self-obtained vaginal swabs and first-catch urine samples separately and in combination for the detection of Chlamydia trachomatis by two amplified DNA assays in women visiting a sexually transmitted disease clinic. Clin Microbiol Infect 17:S754–S755.

15. Hadgu A. 1999. Discrepant analysis: a biased and an unscientific method for estimating test sensitivity and specificity. J Clin Epidemiol 52:1231–7.

16. Martin DH, Nsuami M, Schachter J, Iii EWH, Ferrero D, Quinn TC, Gaydos C, Hook EW 3rd, Ferrero D, Quinn TC, Gaydos C. 2004. Use of multiple nucleic acid amplification tests to define the infected-patient “gold standard” in clinical trials of new diagnostic tests for Chlamydia trachomatis infections. Society 42:4749–4758.

17. Hadgu A, Dendukuri N, Wang L. 2012. Evaluation of screening tests for detecting Chlamydia trachomatis: bias associated with the patient-infected-status algorithm. Epidemiology 23:72–82.

18. Moher D, Liberati A, Tetzlaff J, Altman DG, Group P. 2009. Preferred reporting items for systematic reviews and meta-analyses: the PRISMA statement. BMJ 339:b2535.

19. Bignell C, Unemo M. 2013. 2012 European guideline on the diagnosis and treatment of gonorrhoea in adults. Int J STD AIDS 24:85–92.

20. Whiting PF, Rutjes AWS, Westwood ME, Mallett S, Deeks JJ, Reitsma JB, Leeflang MMG, Sterne JAC, Bossuyt PMM. 2011. QUADAS-2: a revised tool for the quality assessment of diagnostic accuracy studies. Ann Intern Med 155:529–36.

21. R Core Team. 2017. R: A language and environment for statistical computing. R Foundation for Statistical Computing, Vienna, Austria.

22. Chernesky MA, Hook EW, Martin DH, Lane J, Johnson R, Jordan JA, Fuller D, Willis DE, Fine PM, Janda WM, Schachter J. 2005. Women find it easy and prefer to collect their own vaginal swabs to diagnose Chlamydia trachomatis or Neisseria gonorrhoeae infections. Sex Transm Dis 32:729–33.

23. Hook EW 3rd, Ching SF, Stephens J, Hardy KF, Smith KR, Lee HH. 1997. Diagnosis of Neisseria gonorrhoeae infections in women by using the ligase chain reaction on patient-obtained vaginal swabs. J Clin Microbiol 35:2129–2132.

24. Le Roy C, Le Hen I, Clerc M, Arfel V, Normandin F, Bebear C, de Barbeyrac B, Bébéar C, de Barbeyrac B. 2012. The first performance report for the Bio-Rad Dx CT/NG/MG assay for simultaneous detection of Chlamydia trachomatis, Neisseria gonorrhoeae and Mycoplasma genitalium in urogenital samples. J Microbiol Methods 89:193–197.

25. Gaydos CA, Cartwright CP, Colaninno P, Welsch J, Holden J, Ho SY, Webb EM, Anderson C, Bertuzis R, Zhang L, Miller T, Leckie G, Abravaya K, Robinson J. 2010. Performance of the Abbott RealTime CT/NG for detection of Chlamydia trachomatis and Neisseria gonorrhoeae. J Clin Microbiol 48:3236–43.

26. Shipitsyna E, Zolotoverkhaya E, Agne-Stadling I, Krysanova A, Savicheva A, Sokolovsky E, Domeika M, Unemo M. 2009. First evaluation of six nucleic acid amplification tests widely used in the diagnosis of Chlamydia trachomatis in Russia. J Eur Acad Dermatology Venereol 23:268–276.

27. Chernesky MA, Jang D, Luinstra K, Chong S, Smieja M, Cai W, Hayhoe B, Portillo E, Main C, Ewert R, Macritchie C, Main C, Ewert R, Main MacRitchie C, Ewert R. 2006. High analytical

For peer review only - http://bmjopen.bmj.com/site/about/guidelines.xhtml

sensitivity and low rates of inhibition may contribute to detection of Chlamydia trachomatis in significantly more women by the APTIMA Combo 2 assay. J Clin Microbiol 44:400–405.

28. Cosentino LA, Landers D V, Hillier SL. 2003. Detection of Chlamydia trachomatis and Neisseria gonorrhoeae by strand displacement amplification and relevance of the amplification control for use with vaginal swab specimens. J Clin Microbiol 41:3592–6.

29. Stary A, Schuh E, Kerschbaumer M, Götz B, Lee H. 1998. Performance of transcription-mediated amplification and ligase chain reaction assays for detection of chlamydial infection in urogenital samples obtained by invasive and noninvasive methods. J Clin Microbiol 36:2666–70.

30. Schachter J, Chernesky MA, Willis DE, Fine PM, Martin DH, Fuller D, Jordan JA, Janda W, Hook EW. 2005. Vaginal swabs are the specimens of choice when screening for Chlamydia trachomatis and Neisseria gonorrhoeae: results from a multicenter evaluation of the APTIMA assays for both infections. Sex Transm Dis 32:725–8.

31. Chernesky M, Jang D, Gilchrist J, Hatchette T, Poirier A, Flandin JF, Smieja M, Ratnam S. 2014. Head to Head Comparison of Second Generation Nucleic Acid Amplification Tests for Chlamydia trachomatis and Neisseria gonorrhoeae on Female Urines and Self-Collected Vaginal Swabs. J Clin Microbiol 52:2305–10.

32. Fang J, Husman C, DeSilva L, Chang R, Peralta L. 2008. Evaluation of self-collected vaginal swab, first void urine, and endocervical swab specimens for the detection of Chlamydia trachomatis and Neisseria gonorrhoeae in adolescent females. J Pediatr Adolesc Gynecol 21:355–360.

33. Koumans EH, Black CM, Markowitz LE, Unger ER, Pierce A, Sawyer MK, Papp JR. 2003. Comparison of Methods for Detection of Chlamydia trachomatis and Neisseria gonorrhoeae Using Commercially Available Nucleic Acid Amplification Tests and a Liquid Pap Smear Medium. Society 41:1507–1511.

34. Van Der Pol B, Williams JA, Fuller DA, Taylor SN, Hook EW. 2017. Combined testing for chlamydia, gonorrhea, and trichomonas by use of the BD max CT/GC/TV assay with genitourinary specimen types. J Clin Microbiol 55:155–164.

For peer review only

For peer review only - http://bmjopen.bmj.com/site/about/guidelines.xhtml

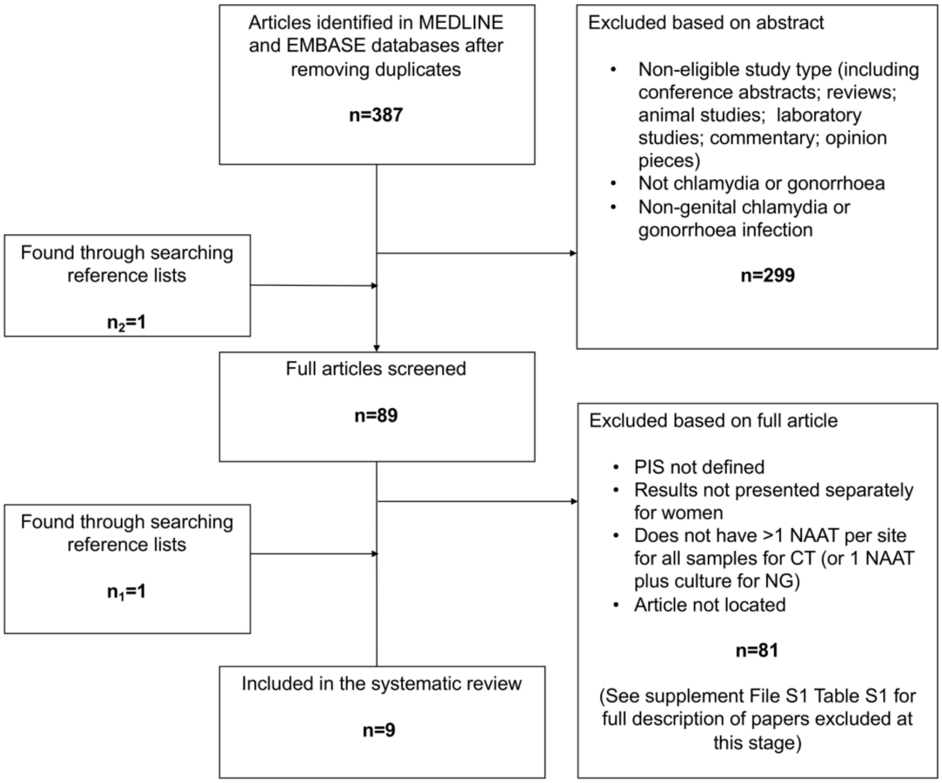

Figure 1. Flowchart of study selection

181x142mm (300 x 300 DPI)

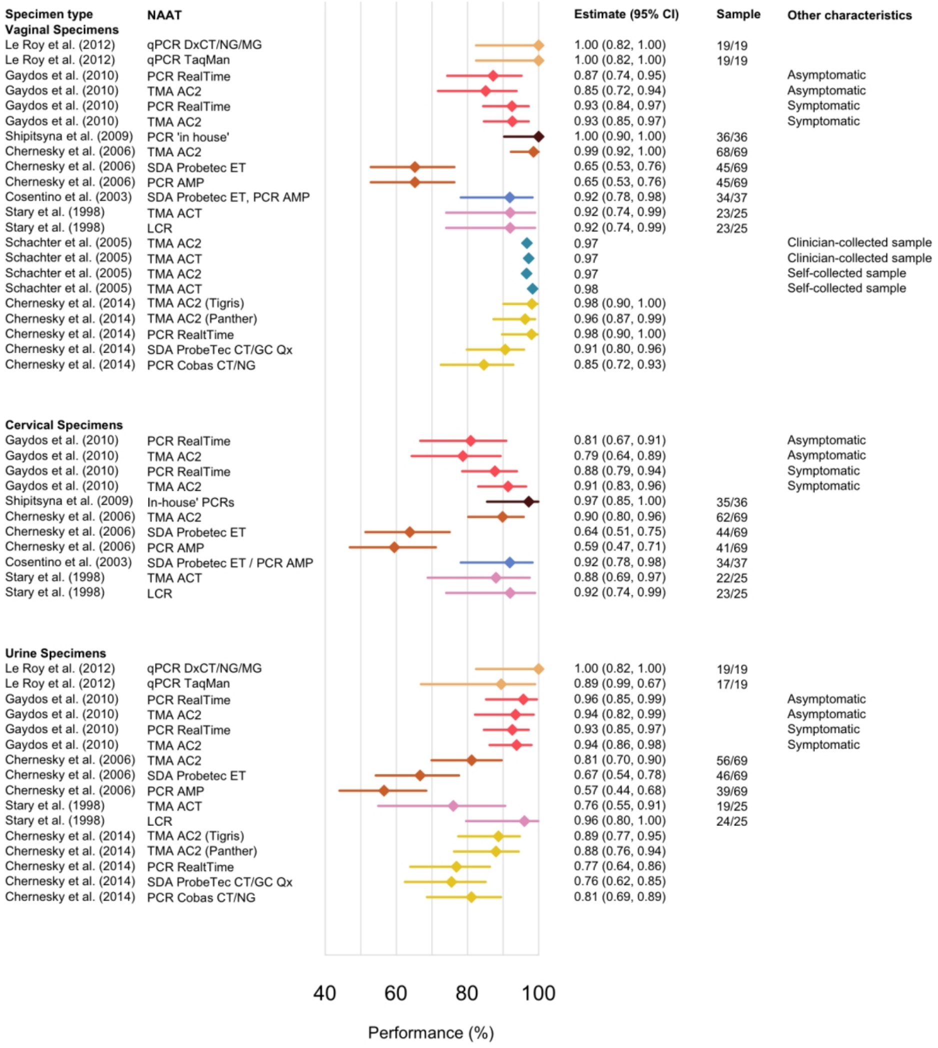

Figure 2. Performance estimates for chlamydia by showing the sensitivity of the NAAT on a given specimen site relative to PIS. The studies are referenced by the first author, and publication year, and colors are by study for clarity.

146x173mm (300 x 300 DPI)

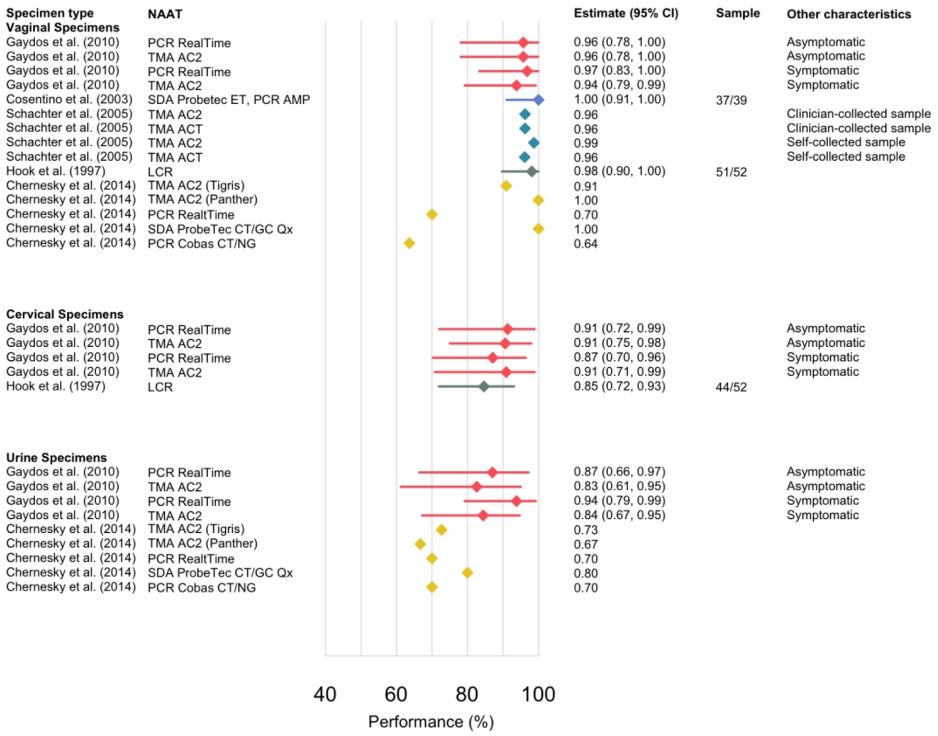

Figure 3. Performance estimates for gonorrhea showing the sensitivity of the NAAT on a given specimen site relative to PIS. The studies are referenced by the first author, and publication year, and colors are by study for clarity.

146x126mm (300 x 300 DPI)

Supplemental File for

Evaluation of the performance of nucleic acid amplification tests (NAATs) in detection of chlamydia and gonorrhoea infection in vaginal specimens relative to patient infection status: a systematic review

Minttu M. Rönn, Louise Mc Grath-Lone, Bethan Davies, Janet Wilson, Helen Ward

For peer review only

Search strategy

The following Boolean searches were performed on OvidSP on 8 October 2013 and an update on 3 October 2017. We searched all fields (af) using Ovid MEDLINE (1947-present) and EMBASE (1946-present) databases. We combined the individual searches with AND in the following manner:

(chlamydia OR “chlamydia trachomatis”).af OR (gonorrh?ea OR “Neisseria gonorrhoeae”).af

AND

(vagina$.af OR vulvo-vagina$).af

AND

(urine OR endovervi$ OR cervi$).af

AND

(NAAT OR “nucleic acid amplification” OR (PCR OR “polymerase chain reaction”) OR (TMA OR “transcription mediated amplification OR “transcription-mediated amplification”) OR (SDA or “Strand displacement amplification”) OR (LCR or “ligase chain reaction”)).af

AND

(sensitivity OR specificity OR performance).af

AND

(diagnos$ OR screen$ OR test OR detect$).af

For peer review only - http://bmjopen.bmj.com/site/about/guidelines.xhtml

Table S1.Studies which were excluded based on the full article (n=81).

Does not have >1 NAAT per site for all samples for CT (or 1 NAAT plus culture for NG)		Performace calculation not based on PIS	Duplicate data
Bakken et al., 2005 (1)	Schoeman et al., 2012 (2)	Jaton et al., 2006 (3)	Andrews, 2009 (4)
Behets et al., 2002 (5)	Schmitt et al, 2014 (6)	Le Roy et al., 2013 (7)	
Berwald et al., 2009(8)	Shrier et al., 2004 (9)	Shalepo et al., 2006 (10)	**Study population all infected**
Black et al., 2009 (11)	Skidmore et al., 2006 (12)	Schachter et al., 2003 (13)	Bachmann et al., 2002 (14)
Carder et al., 1999 (15)	Stewart et al., 2012 (16)	Samra et al., 2011 (17)	
Chandeying et al., 2004 (18)	Sturm et al., 2004 (19)	Upton et al., 2013 (20)	
Cheng et al. 2014 (21)	Tanaka et al., 1998 (22)	Vahidnia et al., 2014 (23)	
Dietrich et al., 2010 (24)	Tanaka et al., 2000 (25)	Yu et al., 2016(26)	**No vaginal samples**
Domeika & Drulyte, 2000 (27)	Taylor et al., 2011 (28)	Zauli et al., 2013 (29)	
Domeika et al.,1999 (30)	Thomas et al., 1998 (31)		Carroll et al. 1998 (32)
Falk et al., 2010 (33)	Thomas et al., 2001 (34)		Lee et al., 2013 (35)
Fang et al., 2008 (36)	Van Der Pol, 2012 (37)		Nunez-Forero et al. 2015 (38)
Fernandez et al., 2016 (39)	Van Der Pol et al., 2012 (28)		
Garrow et al., 2002 (40)	Van Der Pol et al., 2013 (41)	**No primary data reported**	
Gaydos et al., 2013 (42)	Van Der Pol et al., 2015 (43)	Chernesky & Jang, 2006 (44)	**Only vaginal samples**
Gokral et al., 2005 (45)	Van Der Pol et al., 2017 (46)	Fong & Christian, 2012 (47)	Gaydos et al. 2002 (48)
		Gaydos et al., 2014 (49)	
Hjelm et al., 2001 (50)	Van Dommelen et al., 2011 (51)	Meyer, 2012 (52)	
Hsieh et al., 2003 (53)	Wiesenfeld et al., 1996 (54)	Sautter et al., 2009 (55)	

For peer review only

Keane et al., 2007 (56)	Witkin et al., 1996 (57)	Van der Pol et al. 2017 (58)	**Performance calculation not based on PIS**
Knox et al., 2002 (59)			Kellogg et al. 2004 (60)
Kriesel et al., 2016 (61)		**Paper not available/located**	Tayoun et al. 2015 (62)
Li et al.2014., (63)			
Lawton et al. 2013 (64)		Lanjouw et al., 2009 (65)	**Results not stratified by sample site**
Macmillan et al., 2003 (66)		Ostergaard et al., 1997 (67)	McKechnie et al., 2011 (68)
Macmillan et al., 2003(69)		Cheng et al., 2014 (70)	
Ostergaard & Moller, 1995 (71)			
Ostergaard et al. 1996 (72)		**Cervical & vaginal samples combined**	
Polaneczky et al., 1998 (73)			
Renton et al., 2006 (74)		Koumans et al., 2003 (75)	
Rompalo et al., 2001 (76)		Leslie et al., 2003 (77)	
Rumyantseva et al 2015 (78)			

For peer review only

Modified Quality Assessment for Diagnostic Accuracy Studies (QUADAS-2) tool for assessment of included study quality

A modified tool, adapted from QUADAS-2 (79) was used to evaluate the following aspects of the included studies:

- **Risk of bias in patient selection:** Were patients selected at random (or in a consecutive manner of patients attending the clinic)? Were risk factors for the outcomes used to select patients included?
- **Risk of bias in patient infection status (PIS) definition:** Was PIS clearly defined? Had vaginal swabs been included in the PIS definition?
- **Risk of bias in flow and timing:** Is there a lag between the different tests done which could affect the results of the test? Were the same tests conducted for all patients (for example, was discrepant analysis used for equivocal test results?)?
- **Applicability concerns in patient selection:** Did the patient and the setting match the research question?
- **Applicability concerns for PIS definition:** Is the definition of PIS different from the review question?

Table S2. QUADAS-2 assessment for each included study

	Risk of bias			Applicability concerns	
Reference	Patient selection	PIS definition	Flow and timing	Patient selection	PIS definition
Chernesky *et al.*, 2006 (80)	Unclear	High	High	Unclear	Low
Chernesky *et al.*, 2014 (81)	Unclear	High	Low	Low	Low
Cosentino *et al.*, 2003 (82)	Unclear	High	High	High	Low
Gaydos *et al.*, 2010 (83)	Unclear	High	Unclear	Low	Low
Hook *et al.*, 1997 (84)	Unclear	High	High	Low	Low
Le Roy *et al.*, 2012 (85)	High	High	High	Low	High
Schachter *et al.*, 2005 (86)	Unclear	Low	Low	Low	Low
Shipitsyna *et al.*, 2009 (87)	High	High	Unclear	High	High
Stary et al., 1998 (88)	High	High	High	High	High

Overall, the sampling of patients to studies was not well described (unclear risk of bias) or selected patients with risk factors for the outcome (high risk of bias). All but Schachter *et al.* had included vaginal samples in their PIS definition which we considered likely to introduce a high risk of bias for the performace estimate (as discussed in the manuscript).

For flow and timing, the majority of studies were judged to have high risk of bias as they had used discrepant analysis (additional tests performed on patients with equivocal results) in their analyses or they had a different testing for some of the patients. There were less applicability concerns as concerns about risk of bias. Applicability of patient selection was unclear if the patient selection was poorly defined and of high concern if the patient population was at increased risk for the outcome (reported symptoms or were contacts of infected partners or reported high risk behaviours). PIS definition was marked as high concern if it was poorly explained (Shipitsyna *et al.*) or the performace estimate was not directly reported (Stary and Le Roy).

For peer review only - http://bmjopen.bmj.com/site/about/guidelines.xhtml
For peer review only

Table S3. Summary of results from studies that estimated performace of vaginal specimens relative to PIS for chlamydia

Author and year	Number of women who had chlamydia test	Chlamydia infected as defined by PIS (prevalence)	Sample performace relative to PIS (95% CI) [numbers reported by studies]		
			Vaginal samples	Endocervical samples	Urine
Chernesky M. et al. (2006)	298	69 (23%)	(AC2 against PIS)<br>99% (92-100%) [68/69]<br>(PT against PIS)<br>65% (53-76%) [45/69]<br>(AMP against PIS)<br>65% (52-76%) [45/69]	(AC2 against PIS)<br>90% (80-96%) [62/69]<br>(PT against PIS)<br>64% (51-75%) [44/69]<br>(AMP against PIS)<br>59% (47-71%) [41/69]	(AC2 against PIS)<br>81% (70-90%) [56/69]<br>(PT against PIS)<br>67% (54-78%) [46/69]<br>(AMP against PIS)<br>57% (44-68%) [39/69]
Chernesky M. *et al.*, (2014)	575	53 (9%)	AC2 (Tigris)<br>98.1 (90.1-99.7%)<br>AC2 (Panther)<br>96.2 (87.3-99.0%)<br>Real*Time* CT/NG<br>98.0 (89.7-99.7%)<br>ProbeTec CT/GC Qx<br>90.6 (79.8-95.9%)<br>Cobas CT/NG<br>84.6 (72.5-92.0%)	n/a	AC2 (Tigris)<br>88.7 (77.4-94.7%)<br>AC2 (Panther)<br>88.0 (76.2-94.4%)<br>Real*Time* CT/NG<br>76.9 (63.9—86.3%)<br>ProbeTec CT/GC Qx<br>75.5 (62.4-85.1%)<br>Cobas CT/NG<br>81.1 (68.6-89.4%)
Cosentino L.A. et al. (2003)	455	37 (8.1%)	(SDA against PIS) – SDA available for all sites<br>92% (78-98%) [34/37]	(SDA against PIS)<br>92% 78-98%) [34/37]	n/a
Gaydos C.A. et al. (2010)	2014 women enrolled	(Overall prevalence 9%)	Clinician-collected<br>Estimate and range presented<br>(ABT against PIS)<br>Symptomatic : 93% (84–97%)<br>Asymptomatic: 87% (74–95%)<br>(GP AC2 against PIS)<br>Symptomatic: 93% (85–97%)<br>Asymptomatic: 85% (72–94%)	(ABT against PIS)<br>Estimate and range presented<br>Symptomatic: 88% (79–94%)<br>Asymptomatic: 81% (67–91%)<br>(GP AC2 against PIS)<br>Symptomatic: 91% (83-97%)<br>Asymptomatic: 79% (64–89%)	(ABT against PIS)<br>Estimate and range presented<br>Symptomatic: 93% (85–97%)<br>Asymptomatic: 96% (85–100%)<br>(GP AC2 against PIS)<br>Symptomatic: 94% (86–98%)<br>Asymptomatic: 94% (82–99%)

For peer review only

Le Roy C. et al. (2012)	193	19 included in this analysis. Prevalence in the study was 20/193 (10%).	Bio-Rad Dx CT/NG/MG<br>100 % (82-100%) [19/19]<br><br>Cobas<br>100% (82-100%) [19/19]	Not evaluated as only 21 individuals had EC swab taken (3 Ct positives)	Bio-Rad Dx CT/NG/MG<br>100% (82-100%) [19/19]<br><br>Cobas<br>89% (67-99%) [17/19]
Schachter J et al. (2005)	1451	180<br>(12%)	Clinician-collected<br>Aptima Combo: 97%<br>Aptima CT: 97%<br><br>Patient-collected<br>Aptima Combo: 97%<br>Aptima CT: 98%	n/a	n/a
Shipitsyna E. et al. (2009)	298 (with both EC and vagina)	36<br>(12%)	100% (85-100%) [36/36]	97% (85-100%) [35/36]	n/a
Stary A et al. (1998)	308	25<br>(8%)	(TMA against PIS)<br>92% (74-99%) [23/25]<br><br>(LCR against PIS)<br>92%(74-99%) [23/25]	(TMA against PIS)<br>88% (69-97%) [22/25]<br><br>(LCR against PIS)<br>92% (74-99%) [23/25]	(TMA against PIS)<br>76% (55-91%) [19/25]<br><br>(LCR against PIS)<br>96% (80-100%) [24/25]

For peer review only

Table S4. **Summary of results from studies that estimated performace of vaginal specimens relative to PIS for gonorrhoea**

Author and year	Number of women who had gonorrhoea test	Gonorrhoea infected as defined by PIS (prevalence)	Sample performace relative to PIS		
			Vaginal	Endocervical	Urine
Chernesky M. *et al.*, (2014)	575	11 (2%)	AC2 (Tigris): 90.9%<br>AC2 (Panther): 100%<br>RealTime CT/NG: 70.0%<br>ProbeTec CT/GC Qx:100%<br>Cobas CT/NG:63.6%	n/a	AC2 (Tigris): 72.7%<br>AC2 (Panther): 66.7%<br>RealTime CT/NG: 70.0%<br>ProbeTec CT/GC Qx: 80.0%<br>Cobas CT/NG: 70.0%
Cosentino L.A. et al. (2003)	455	39 (8.6%)	100% (91-100%) [39/39]	100% (91-100%) [39/39]	n/a
Gaydos C.A. et al. (2010)	2014 women enrolled	Overall prevalence 3.8%	Clinician-collected<br>Estimate and range presented (ABT (RealTime) against PIS)<br>Symptomatic: 97% (83–100%)<br>Asymptomatic: 96% (78–100%)<br><br>(GP AC2 against PIS)<br>Symptomatic: 94% (79–99%)<br>Asymptomatic: 96% (78–100%)	Estimate and range presented (ABT (RealTime) against PIS)<br>Symptomatic: 87% (70–96%)<br>Asymptomatic: 91% (72–99%)<br><br>(GP AC2 against PIS)<br>Symptomatic: 91% (75–98%)<br>Asymptomatic: 91% (71%–99%)	Estimate and range presented (ABT (RealTime) against PIS)<br>Symptomatic: 94% (79–99%)<br>Asymptomatic: 87% (66–97%)<br><br>(GP AC2 against PIS)<br>Symptomatic: 84% (67–95%)<br>Asymptomatic:83% (61-95%)
Hook, E et al. (1997)	309	52 (16.8%)	Patient-obtained<br>100% (93-100%) [52/52]	85% (72-93%) [44/52]	
Le Roy et al. (2012)	193	2 (1%)	Not evaluated.		
Schachter J et al. (2005)	1450	78 (5.4%)	Clinician-collected<br>Aptima Combo: 96%<br>Aptima GC: 96%<br><br>Patient-collected<br>Aptima Combo: 99%<br>Aptima GC: 96%		

For peer review only

References

1. Bakken IJ, Bratt H, Skjeldestad FE, Nordbø SA. 2005. [Detection of chlamydia trachomatis in urine, vulval and cervical swabs]. Tidsskr den Nor lægeforening Tidsskr Prakt Med ny række 125:1629–30.
2. Schoeman SA, Stewart CMW, Booth RA, Smith SD, Wilcox MH, Wilson JD. 2012. Assessment of best single sample for finding chlamydia in women with and without symptoms: a diagnostic test study. BMJ 345:e8013.
3. Jaton K, Bille J, Greub G. 2006. A novel real-time PCR to detect Chlamydia trachomatis in first-void urine or genital swabs. J Med Microbiol 55:1667–1674.
4. Andrews J. 2009. Self-administered vaginal swabs were accurate for screening for sexually transmitted infections in the emergency department. Evid Based Med 14:150.
5. Behets FM-TF, Andriamiadana J, Randrianasolo D, Rasamilalao D, Ratsimbazafy N, Dallabetta G, Cohen MS. 2002. Laboratory diagnosis of sexually transmitted infections in women with genital discharge in Madagascar: implications for primary care. Int J STD AIDS 13:606–611.
6. Schmitt M, Depuydt C, Stalpaert M, Pawlita M. 2014. Bead-based multiplex sexually transmitted infection profiling. J Infect 69:123–133.
7. Le Roy C, Papaxanthos A, Liesenfeld O, Mehats V, Clerc M, Bébéar C, de Barbeyrac B. 2013. Swabs (dry or collected in universal transport medium) and semen can be used for the detection of Chlamydia trachomatis using the cobas 4800 system. J Med Microbiol 62:217–22.
8. Berwald N, Cheng S, Augenbraun M, Abu-Lawi K, Lucchesi M, Zehtabchi S. 2009. Self-administered vaginal swabs are a feasible alternative to physician-assisted cervical swabs for sexually transmitted infection screening in the emergency department. Acad Emerg Med 16:360–3.
9. Shrier L, Dean D, Klein E, Harter K, Rice P. 2004. Limitations of Screening Tests for the Detection of Chlamydia trachomatis in Asymptomatic Adolescent and Young Adult Women. Obs Gynecol Surv 59:513–515.
10. Shalepo K, Savicheva A, Shipitsyna E, Unemo M, Domeika M. 2006. Diagnosis of Chlamydia trachomatis in Russia - In-house PCR assays may be effective but overall optimization and quality assurance are urgently needed. Apmis 114:500–507.
11. Black CM, Driebe EM, Howard L a, Fajman NN, Sawyer MK, Girardet RG, Sautter RL, Greenwald E, Beck-Sague CM, Unger ER, Igietseme JU, Hammerschlag MR, MD P, Igietseme JU, Hammerschlag MR, C.M. B-SB, E.M. D, L.A. H, N.N. F, M.K. S, R.G. G, R.L. S, E. G, E.R. U, J.U. I, M.R. H. 2009. Multicenter study of nucleic acid amplification tests for detection of Chlamydia trachomatis and Neisseria gonorrhoeae in children being evaluated for sexual abuse. Pediatr Infect Dis J 28:608–613.
12. Skidmore S, Horner P, Herring A, Sell J, Paul I, Thomas J, Caul EO, Egger M, McCarthy E, Sanford E, Salisbury C, Macleod J, Sterne J, Low L, Group CSS (ClaSS) P. 2006. Vulvovaginal-swab or first-catch urine specimen to detect Chlamydia trachomatis in women in a community setting?. J Clin Microbiol 44:4389–4394.
13. Schachter J, McCormack WM, Chernesky MA, Martin DH, Van Der Pol B, Rice PA, Hook EW 3rd, Stamm WE, Quinn TC, Chow JM. 2003. Vaginal swabs are appropriate specimens for diagnosis of genital tract infection with Chlamydia trachomatis. J Clin Microbiol 41:3784–3789.
14. Bachmann LLH, Desmond RA, Stephens J, Hughes A, Hook EW, Stephens R, Hughes A, Hook III EW. 2002. Duration of persistence of gonococcal DNA detected by ligase chain reaction in men and women following recommended therapy for uncomplicated gonorrhea. J Clin Microbiol 40:3596–3601.

For peer review only - http://bmjopen.bmj.com/site/about/guidelines.xhtml

15. Carder C, Robinson AJ, Broughton C, Stephenson JM, Ridgway GL. 1999. Evaluation of self-taken samples for the presence of genital Chlamydia trachomatis infection in women using the ligase chain reaction assay. Int J STD AIDS 10:776–779.

16. Stewart CMWCMW, Schoeman SA, Booth RA, Smith SDSDD, Wilcox MHMHH, Wilson JDJDD. 2012. Assessment of self taken swabs versus clinician taken swab cultures for diagnosing gonorrhoea in women: single centre, diagnostic accuracy study. BMJ 345:e8107.

17. Samra Z, Rosenberg S, Madar-Shapiro L. 2011. Direct simultaneous detection of 6 sexually transmitted pathogens from clinical specimens by multiplex polymerase chain reaction and auto-capillary electrophoresis. Diagn Microbiol Infect Dis 70:17–21.

18. Chandeying V, Skov S, Farrell D, Lamlertkittikul S, Tabrizi S, Jarumanokul R. 2002. GYNAECOLOGY A Comparison of First-Void Urine , Self-Administered Low Vaginal Swab , Self-Inserted Tampon , and Endocervical Swab Using PCR Tests for the Detection of Infection with Chlamydia Trachomatis in Commercial Sex Workers and Women Attending Gyneco 14:201–209.

19. Sturm PDJ, Connolly C, Khan N, Ebrahim S, Sturm a W. 2004. Vaginal tampons as specimen collection device for the molecular diagnosis of non-ulcerative sexually transmitted infections in antenatal clinic attendees. Int J STD AIDS 15:94–8.

20. Upton A, Wilson J, Bissessor L. 2013. Introduction of routine polymerase chain reaction testing for Neisseria gonorrhoeae in a community laboratory. Sex Health. ELEC, CSIRO (P.O. Box 1139, Collingwood VIC 3066, Australia), A. Upton, Labtests, 27-41 Carbine Road, Mt Wellington 1060, New Zealand. E-mail: arlo.upton@labtests.co.nz.

21. Cheng A, Kirby JE. 2014. Evaluation of the hologic gen-probe PANTHER, APTIMA combo 2 assay in a tertiary care teaching hospital. Am J Clin Pathol 141:397–403.

22. Tanaka M, Nakayama H, Yoshida H, Takahashi K, Nagafuji T, Hagiwara T, Kumazawa J. 1998. Detection of Chlamydia trachomatis in vaginal specimens from female commercial sex workers using a new improved enzyme immunoassay. Sex Transm Infect 74:435–438.

23. Vahidnia A, Costa S, Veenings S, Tuin H, van Loon L, Bliekendaal H. 2014. Comparative evaluation of Roche Aurora FLOW, Becton and Dickinson Viper system, and Dynex DS2 for detection of Chlamydia trachomatis, Neisseria gonorrhoeae, and Trichomonas vaginalis in various clinical specimens. Diagn Microbiol Infect Dis 80:191–192.

24. Dietrich W, Rath M, Stanek G, Apfalter P, Huber JC, Tempfer C. 2010. Multiple site sampling does not increase the sensitivity of Chlamydia trachomatis detection in infertility patients. Fertil Steril 93:68–71.

25. Tanaka M, Nakayama H, Sagiyama K, Haraoka M, Yoshida H, Hagiwara T, Akazawa K, Naito S. 2000. Evaluation of a new amplified enzyme immunoassay (EIA) for the detection of Chlamydia trachomatis in male urine , female endocervical swab , and patient obtained vaginal swab specimens. J Clin Pathol 53:350–354.

26. Yu B, An Y, Xu G, Shan H. 2016. Detection of *Chlamydia trachomatis* and *Neisseria gonorrhoea* e based on cross-priming amplification. Lett Appl Microbiol 62:399–403.

27. Domeika M, Drulyte O. 2000. Use of PCR for the detection of genital Chlamydia trachomatis infection on self-obtained mailed vaginal samples. Acta Obstet Gynecol Scand 79:570–575.

28. Taylor SN, Van Der Pol B, Lillis R, Hook EW 3rd, Lebar W, Davis T, Fuller D, Mena L, Fine P, Gaydos CA, Martin DH. 2011. Clinical evaluation of the BD ProbeTecTM Chlamydia trachomatis Qx amplified DNA assay on the BD ViperTM system with XTRTM technology. Sex Transm Dis 38:603–609.

For peer review only - http://bmjopen.bmj.com/site/about/guidelines.xhtml

29. Zauli DAG, De Menezes CLP, De Oliveira CL. 2013. Development and padronization of three multiplex PCRs for the diagnosis of chlamydia trachomatis, toxoplasma gondii, herpes simplex viruses 1 and 2, and cytomegalovirus. Mol Biotechnol 54:1004–1009.

30. Domeika M, Bassiri M, Butrimiene I, Venalis a, Ranceva J, Vasjanova V. 1999. Evaluation of vaginal introital sampling as an alternative approach for the detection of genital Chlamydia trachomatis infection in women. Acta Obstet Gynecol Scand 78:131–136.

31. Thomas BJ, Pierpoint T, Taylor-Robinson D, Renton M. 1998. Sensitivity of the ligase chain reaction assay for detecting Chlamydia trachomatis in vaginal swabs from women who are infected at other sites. Sex Transm Infect 74:140–1.

32. Carroll KC, Aldeen WE, Morrison M, Anderson R, Lee D, Mottice S. 1998. Evaluation of the Abbott LCx ligase chain reaction assay for detection of Chlamydia trachomatis and Neisseria gonorrhoeae in urine and genital swab specimens from a sexually transmitted disease clinic population. J Clin Microbiol 36:1630–1633.

33. Falk L, Coble B-I, Mjörnberg P, Fredlund H. 2010. Sampling for Chlamydia trachomatis infection - a comparison of vaginal, first-catch urine, combined vaginal and first-catch urine and endocervical sampling. Int J STD AIDS 21:283–287.

34. Thomas BJ, Pierpoint T, Taylor-Robinson D, Renton M. 2001. Qualitative and quantitative aspects of the ligase chain reaction assay for Chlamydia trachomatis in genital tract samples and urines. Int J STD AIDS 12:589–594.

35. Lee GI, Yoen JP, Kang JS, Hwang SY, Hong YM, Yang JH, Yoon HK. 2013. A comparison of oligonucleotide-based microarray and real-time PCR for the detection of sexually transmitted infections. Biochip J 7:68–74.

36. Fang J, Husman C, DeSilva L, Chang R, Peralta L. 2008. Evaluation of self-collected vaginal swab, first void urine, and endocervical swab specimens for the detection of Chlamydia trachomatis and Neisseria gonorrhoeae in adolescent females. J Pediatr Adolesc Gynecol 21:355–360.

37. Van Der Pol B, Taylor SN, Lebar W, Davis T, Fuller D, Mena L, Fine P, Gaydos C a., Martin DH, Hook EW. 2012. Clinical Evaluation of the BD ProbeTec™ Neisseria gonorrhoeae Qx Amplified DNA Assay on the BD Viper™ System With XTR™ Technology. Sex Transm Dis 39:147–153.

38. Nuñez-Forero L, Moyano-Ariza L, Gaitán-Duarte H, Ángel-Müller E, Ruiz-Parra A, González P, Rodríguez A, Tolosa JE. 2016. Diagnostic accuracy of rapid tests for sexually transmitted infections in symptomatic women. Sex Transm Infect 92:24–28.

39. Fernández G, Martró E, González V, Saludes V, Bascuñana E, Marcó C, Rivaya B, López E, Coll P, Matas L, Ausina V. 2016. Usefulness of a novel multiplex real-time PCR assay for the diagnosis of sexually-transmitted infections. Enferm Infecc Microbiol Clin 34:471–6.

40. Garrow SC, Smith DW, Harnett GB. 2002. The diagnosis of chlamydia, gonorrhoea, and trichomonas infections by self obtained low vaginal swabs, in remote northern Australian clinical practice. Sex Transm Infect 78:278–281.

41. Van Der Pol B, Taylor SN, Liesenfeld O, Williams JA, Hook EW. 2013. Vaginal swabs are the optimal specimen for detection of genital Chlamydia trachomatis or Neisseria gonorrhoeae using the Cobas 4800 CT/NG test. Sex Transm Dis 40:247–50.

42. Gaydos CA, Van Der Pol B, Jett-Goheen M, Barnes M, Quinn N, Clark C, Daniel GE, Dixon PB, Hook EW 3rd, Group CS. 2013. Performance of the Cepheid CT/NG Xpert Rapid PCR Test for Detection of Chlamydia trachomatis and Neisseria gonorrhoeae. J Clin Microbiol 51:1666–1672.

For peer review only - http://bmjopen.bmj.com/site/about/guidelines.xhtml

43. Van Der Pol B, Hook 3rd EW, Williams JA, Smith B, Taylor SN. 2015. Performance of the BD CTQx and GCQx Amplified Assays on the BD Viper LT Compared With the BD Viper XTR System. Sex Transm Dis 42:521–523.

44. CHERNESKY MA, JANG DA. 2006. APTIMA transcription-mediated amplification assays for Chlamydia trachomatis and Neisseria gonorrhoeae. Expert Rev Mol Diagn 6:519–525.

45. Gokral JS, Mania-Pramanik J, Meherji PK, Mali BN. Introital swab testing for Chlamydia trachomatis in a resource-poor setting: an Indian perspective. Int J Fertil Womens Med 50:140–3.

46. Van Der Pol B, Williams JA, Fuller DA, Taylor SN, Hook EW. 2017. Combined testing for chlamydia, gonorrhea, and trichomonas by use of the BD max CT/GC/TV assay with genitourinary specimen types. J Clin Microbiol 55:155–164.

47. Fong H, Christian CW. 2012. Evaluating Sexually Transmitted Infections in Sexually Abused Children: New Techniques to Identify Old Infections. Clin Pediatr Emerg Med 13:202–212.

48. Gaydos C, Crotchfelt K, Shah N, Tennant M, Quinn TC, Gaydos JC, McKee KT, Rompalo AM. 2002. Evaluation of dry and wet transported intravaginal swabs in detection of Chlamydia trachomatis and Neisseria gonorrhoeae infections in female soldiers by PCR. J Clin Microbiol 40:758–761.

49. Gaydos CA. 2014. Review of use of a new rapid real-time PCR, the Cepheid GeneXpert ® (Xpert) CT/NG assay, for *Chlamydia trachomatis* and *Neisseria gonorrhoeae* : results for patients while in a clinical setting. Expert Rev Mol Diagn 14:135–137.

50. Hjelm E, Hallén a., Domeika M. 2001. Cervical, urine and vaginal specimens for detection of Chlamydia trachomatis by ligase chain reaction in women: A comparison. Acta Derm Venereol 81:285–288.

51. van Dommelen L, Dukers-Muijrers N, Van Tiel F, Brouwers E, Hoebe C. 2011. Evaluation of one-sample testing of self-obtained vaginal swabs and first-catch urine samples separately and in combination for the detection of Chlamydia trachomatis by two amplified DNA assays in women visiting a sexually transmitted disease clinic. Clin Microbiol Infect 17:S754–S755.

52. Meyer T, T. M. 2012. [Diagnosis and treatment of Chlamydia trachomatis infections]. Hautarzt 63:16–23.

53. Hsieh Y-H, Howell MR, Gaydos JC, Mckee KT, Quinn TC, Gaydos C a, MD MsPH, Mckee KT, Quinn TC, Gaydos C a. 2003. Preference among female Army recruits for use of self-administrated vaginal swabs or urine to screen for Chlamydia trachomatis genital infections. Sex Transm Dis 30:769–773.

54. Wiesenfeld HC, Heine RP, Rideout a., Macio I, DiBiasi F, Sweet RL. 1996. The vaginal introitus: A novel site for Chlamydia trachomatis testing in women. Am J Obstet Gynecol 174:1542–1546.

55. Sautter RL, LeBar WD, Greenwald E. 2009. The Laboratory's Role in Evaluating Sexually Transmitted Diseases as a Result of Sexual Abuse. Clin Microbiol Newsl 31:145–150.

56. Keane F, Bendall R, Saulsbury N, Haddon L. 2007. A comparison of self-taken vulvovaginal and cervical samples for the diagnosis of Chlamydia trachomatis infection by polymerase chain reaction. Int J STD AIDS 18:98–100.

57. Witkin SS, Inglis SR, Polaneczky M. 1996. Detection of Chlamydia trachomatis and Trichomonas vaginalis by polymerase chain reaction in introital specimens from pregnant women. Am J Obstet Gynecol 175:165–167.

For peer review only - http://bmjopen.bmj.com/site/about/guidelines.xhtml

58. Van Der Pol B. 2017. Profile of the triplex assay for detection of chlamydia, gonorrhea and trichomonas using the BD MAX^{TM} System. Expert Rev Mol Diagn 17:539–547.

59. Knox J, Tabrizi SN, Miller P, Petoumenos K, Law M, Chen S, Garland SM. 2002. Evaluation of self-collected samples in contrast to practitioner-collected samples for detection of Chlamydia trachomatis, Neisseria gonorrhoeae, and Trichomonas vaginalis by polymerase chain reaction among women living in remote areas. Sex Transm Dis 29:647–54.

60. Kellogg ND, Baillargeon J, Lukefahr JL, Lawless K, Menard SW. 2004. Comparison of nucleic acid amplification tests and culture techniques in the detection of Neisseria gonorrhoeae and Chlamydia trachomatis in victims of suspected child sexual abuse. J Pediatr Adolesc Gynecol 17:331–339.

61. Kriesel JD, Bhatia AS, Barrus C, Vaughn M, Gardner J, Crisp RJ. 2016. Multiplex PCR testing for nine different sexually transmitted infections. Int J STD AIDS 27:1275–1282.

62. Abou Tayoun AN, Burchard PR, Caliendo AM, Scherer A, Tsongalis GJ. 2015. A multiplex PCR assay for the simultaneous detection of Chlamydia trachomatis, Neisseria gonorrhoeae, and Trichomonas vaginalis. Exp Mol Pathol 98:214–218.

63. Li J, Jang D, Gilchrist J, Smieja M, Ewert R, MacRitchie C, Chernesky M. 2014. Comparison of flocked and Aptima swabs and two specimen transport media in the Aptima combo 2 assay. J Clin Microbiol 52:3808–3809.

64. Lawton B, Bromhead C. 2013. Nucleic acid amplification tests of self-taken vulvovaginal swabs are more sensitive than clinician taken endocervical culture for gonorrhoea. Evid Based Med 18:e46.

65. Lanjouw E, van Daele PLA, Raes MPE, van der Meijden WI. 2009. Consecutively Acquired Sexually Transmitted Infections Mimicking Crohn's Disease. Am J Gastroenterol 104:532–533.

66. Macmillan S, McKenzie H, Templeton A, Strandell A. 2003. Screening for chlamydial infection by DNA testing in urine and vulval swabs was highly sensitive and more acceptable than endocervical swabs. Evidence-based Obstet Gynecol 5:120–121.

67. Ostergaard LJ, Moller JK, Andersen B, Olesen F. 1997. Self-collected and mailed samples for diagnosis of urogenital Chlamydia trachomatis infection in women [Danish]. Ugeskr Laeger 159:5089–92.

68. McKechnie ML, Hillman RJ, Jones R, Lowe PC, Couldwell DL, Davies SC, King F, Kong F, Gilbert GL. 2011. The prevalence of urogenital micro-organisms detected by a multiplex PCR-reverse line blot assay in women attending three sexual health clinics in Sydney, Australia. J Med Microbiol 60:1010–1016.

69. Macmillan S, McKenzie H, Templeton A. 2003. Parallel observation of four methods for screening women under 25 years of age for genital infection with Chlamydia trachomatis. Eur J Obstet Gynecol Reprod Biol 107:68–73.

70. Cheng P, Tang J, Cheng H, Liu X, Wang Y. 2014. PCR methods for simultaneous detection of Neisseria agonorrhoeae, Chlamydiatrachomatis and Ureaplasma parvum. Chinese J Biol 27:109–114.

71. Ostergaard L, Møller JK. 1995. Use of PCR and direct immunofluorescence microscopy for confirmation of results obtained by Syva MicroTrak Chlamydia enzyme immunoassay. J Clin Microbiol 33:2620–3.

72. Ostergaard L, Møller JK, Andersen B, Olesen F. 1996. Diagnosis of urogenital Chlamydia trachomatis infection in women based on mailed samples obtained at home: multipractice comparative study. BMJ 313:1186–9.

73. Polaneczky M, Quigley C, Pollock L, Dulko D, Witkin SS. 1998. Use of self-collected

For peer review only - http://bmjopen.bmj.com/site/about/guidelines.xhtml

vaginal specimens for detection of Chlamydia trachomatis infection. Obstet Gynecol 91:375–8.

74. Renton A, Thomas BM, Gill S, Lowndes C, Taylor-Robinson D, Patterson K. 2006. Chlamydia trachomatis in cervical and vaginal swabs and urine specimens from women undergoing termination of pregnancy. Int J STD AIDS 17:443–447.

75. Koumans EH, Black CM, Markowitz LE, Unger ER, Pierce A, Sawyer MK, Papp JR. 2003. Comparison of Methods for Detection of Chlamydia trachomatis and Neisseria gonorrhoeae Using Commercially Available Nucleic Acid Amplification Tests and a Liquid Pap Smear Medium. Society 41:1507–1511.

76. Rompalo AM, Gaydos CA, Shah N, Tennant M, Crotchfelt KA, Madico G, Quinn TC, Daniel R, Shah K V, Gaydos JC, McKee KT. 2001. Evaluation of use of a single intravaginal swab to detect multiple sexually transmitted infections in active-duty military women. Clin Infect Dis 33:1455–1461.

77. Leslie DE, Azzato F, Ryan N, Fyfe J. 2003. An assessment of the Roche Amplicor ® gonorrhoeae multiplex PCR assay in routine diagnostic use on a variety of specimen types. Commun Dis Intell Q Rep 27:373–9.

78. Rumyantseva T, Golparian D, Nilsson CS, Johansson E, Falk M, Fredlund H, Van Dam A, Guschin A, Unemo M. 2015. Evaluation of the new AmpliSens multiplex real-time PCR assay for simultaneous detection of Neisseria gonorrhoeae, Chlamydia trachomatis, Mycoplasma genitalium, and Trichomonas vaginalis. Apmis 123:879–886.

79. Whiting PF, Rutjes AWS, Westwood ME, Mallett S, Deeks JJ, Reitsma JB, Leeflang MMG, Sterne JAC, Bossuyt PMM. 2011. QUADAS-2: a revised tool for the quality assessment of diagnostic accuracy studies. Ann Intern Med 155:529–36.

80. Chernesky MA, Jang D, Luinstra K, Chong S, Smieja M, Cai W, Hayhoe B, Portillo E, Main C, Ewert R, Macritchie C, Main C, Ewert R, Main MacRitchie C, Ewert R. 2006. High analytical sensitivity and low rates of inhibition may contribute to detection of Chlamydia trachomatis in significantly more women by the APTIMA Combo 2 assay. J Clin Microbiol 44:400–405.

81. Chernesky M, Jang D, Gilchrist J, Hatchette T, Poirier A, Flandin JF, Smieja M, Ratnam S. 2014. Head to Head Comparison of Second Generation Nucleic Acid Amplification Tests for Chlamydia trachomatis and Neisseria gonorrhoeae on Female Urines and Self-Collected Vaginal Swabs. J Clin Microbiol 52:2305–10.

82. Cosentino LA, Landers D V, Hillier SL. 2003. Detection of Chlamydia trachomatis and Neisseria gonorrhoeae by strand displacement amplification and relevance of the amplification control for use with vaginal swab specimens. J Clin Microbiol 41:3592–6.

83. Gaydos CA, Cartwright CP, Colaninno P, Welsch J, Holden J, Ho SY, Webb EM, Anderson C, Bertuzis R, Zhang L, Miller T, Leckie G, Abravaya K, Robinson J. 2010. Performance of the Abbott RealTime CT/NG for detection of Chlamydia trachomatis and Neisseria gonorrhoeae. J Clin Microbiol 48:3236–43.

84. Hook EW 3rd, Ching SF, Stephens J, Hardy KF, Smith KR, Lee HH. 1997. Diagnosis of Neisseria gonorrhoeae infections in women by using the ligase chain reaction on patient-obtained vaginal swabs. J Clin Microbiol 35:2129–2132.

85. Le Roy C, Le Hen I, Clerc M, Arfel V, Normandin F, Bebear C, de Barbeyrac B. 2012. The first performance report for the Bio-Rad Dx CT/NG/MG assay for simultaneous detection of Chlamydia trachomatis, Neisseria gonorrhoeae and Mycoplasma genitalium in urogenital samples. J Microbiol Methods 89:193–197.

86. Schachter J, Chernesky MA, Willis DE, Fine PM, Martin DH, Fuller D, Jordan JA, Janda W, Hook EW. 2005. Vaginal swabs are the specimens of choice when screening for Chlamydia trachomatis and Neisseria gonorrhoeae: results from a

For peer review only - http://bmjopen.bmj.com/site/about/guidelines.xhtml

multicenter evaluation of the APTIMA assays for both infections. Sex Transm Dis 32:725–8.

87. Shipitsyna E, Zolotoverkhaya E, Agne-Stadling I, Krysanova A, Savicheva A, Sokolovsky E, Domeika M, Unemo M. 2009. First evaluation of six nucleic acid amplification tests widely used in the diagnosis of Chlamydia trachomatis in Russia. J Eur Acad Dermatology Venereol 23:268–276.

88. Stary A, Schuh E, Kerschbaumer M, Götz B, Lee H. 1998. Performance of transcription-mediated amplification and ligase chain reaction assays for detection of chlamydial infection in urogenital samples obtained by invasive and noninvasive methods. J Clin Microbiol 36:2666–70.

For peer review only

PRISMA 2009 Checklist

Section/topic	#	Checklist item	Reported on page #
**TITLE**			
Title	1	Identify the report as a systematic review, meta-analysis, or both.	1
**ABSTRACT**			
Structured summary	2	Provide a structured summary including, as applicable: background; objectives; data sources; study eligibility criteria, participants, and interventions; study appraisal and synthesis methods; results; limitations; conclusions and implications of key findings; systematic review registration number.	2-3
**INTRODUCTION**			
Rationale	3	Describe the rationale for the review in the context of what is already known.	4-6
Objectives	4	Provide an explicit statement of questions being addressed with reference to participants, interventions, comparisons, outcomes, and study design (PICOS).	6-7
**METHODS**			
Protocol and registration	5	Indicate if a review protocol exists, if and where it can be accessed (e.g., Web address), and, if available, provide registration information including registration number.	6
Eligibility criteria	6	Specify study characteristics (e.g., PICOS, length of follow-up) and report characteristics (e.g., years considered, language, publication status) used as criteria for eligibility, giving rationale.	6-7
Information sources	7	Describe all information sources (e.g., databases with dates of coverage, contact with study authors to identify additional studies) in the search and date last searched.	7, Supplemental file
Search	8	Present full electronic search strategy for at least one database, including any limits used, such that it could be repeated.	Supplemental file
Study selection	9	State the process for selecting studies (i.e., screening, eligibility, included in systematic review, and, if applicable, included in the meta-analysis).	7-8
Data collection process	10	Describe method of data extraction from reports (e.g., piloted forms, independently, in duplicate) and any processes for obtaining and confirming data from investigators.	8
Data items	11	List and define all variables for which data were sought (e.g., PICOS, funding sources) and any assumptions and simplifications made.	8
Risk of bias in individual studies	12	Describe methods used for assessing risk of bias of individual studies (including specification of whether this was done at the study or outcome level), and how this information is to be used in any data synthesis.	8, Supplemental file
Summary measures	13	State the principal summary measures (e.g., risk ratio, difference in means).	8

For peer review only

PRISMA 2009 Checklist

Synthesis of results	14	Describe the methods of handling data and combining results of studies, if done, including measures of consistency (e.g., I^2) for each meta-analysis.	8

Section/topic	#	Checklist item	Reported on page #
Risk of bias across studies	15	Specify any assessment of risk of bias that may affect the cumulative evidence (e.g., publication bias, selective reporting within studies).	8, Supplemental file
Additional analyses	16	Describe methods of additional analyses (e.g., sensitivity or subgroup analyses, meta-regression), if done, indicating which were pre-specified.	NA
**RESULTS**			
Study selection	17	Give numbers of studies screened, assessed for eligibility, and included in the review, with reasons for exclusions at each stage, ideally with a flow diagram.	8-9<br>Figure 1<br>Supplemental file
Study characteristics	18	For each study, present characteristics for which data were extracted (e.g., study size, PICOS, follow-up period) and provide the citations.	Tables 1 – 2<br>Supplemental file, Tables S3-4
Risk of bias within studies	19	Present data on risk of bias of each study and, if available, any outcome level assessment (see item 12).	Supplemental file, Table S2
Results of individual studies	20	For all outcomes considered (benefits or harms), present, for each study: (a) simple summary data for each intervention group (b) effect estimates and confidence intervals, ideally with a forest plot.	Figures 2-3
Synthesis of results	21	Present results of each meta-analysis done, including confidence intervals and measures of consistency.	NA
Risk of bias across studies	22	Present results of any assessment of risk of bias across studies (see Item 15).	14, Supplemental file
Additional analysis	23	Give results of additional analyses, if done (e.g., sensitivity or subgroup analyses, meta-regression [see Item 16]).	NA
**DISCUSSION**			
Summary of evidence	24	Summarize the main findings including the strength of evidence for each main outcome; consider their relevance to key groups (e.g., healthcare providers, users, and policy makers).	17-18

For peer review only

For peer review only - http://bmjopen.bmj.com/site/about/guidelines.xhtml

PRISMA 2009 Checklist

Limitations	25	Discuss limitations at study and outcome level (e.g., risk of bias), and at review-level (e.g., incomplete retrieval of identified research, reporting bias).	18
Conclusions	26	Provide a general interpretation of the results in the context of other evidence, and implications for future research.	19
**FUNDING**			
Funding	27	Describe sources of funding for the systematic review and other support (e.g., supply of data); role of funders for the systematic review.	20

From: Moher D, Liberati A, Tetzlaff J, Altman DG, The PRISMA Group (2009). Preferred Reporting Items for Systematic Reviews and Meta-Analyses: The PRISMA Statement. PLoS Med 6(7): e1000097. doi:10.1371/journal.pmed1000097

For more information, visit: www.prisma-statement.org.

For peer review only

For peer review only - http://bmjopen.bmj.com/site/about/guidelines.xhtml

The PRISMA for Abstracts Checklist

TITLE	CHECKLIST ITEM	REPORTED ON PAGE #
1. Title:	Identify the report as a systematic review, meta-analysis, or both.	1
**BACKGROUND**		
2. Objectives:	The research question including components such as participants, interventions, comparators, and outcomes.	2
**METHODS**		
3. Eligibility criteria:	Study and report characteristics used as criteria for inclusion.	2
4. Information sources:	Key databases searched and search dates.	2
5. Risk of bias:	Methods of assessing risk of bias.	2
**RESULTS**		
6. Included studies:	Number and type of included studies and participants and relevant characteristics of studies.	2
7. Synthesis of results:	Results for main outcomes (benefits and harms), preferably indicating the number of studies and participants for each. If meta-analysis was done, include summary measures and confidence intervals.	2
8. Description of the effect:	Direction of the effect (i.e. which group is favoured) and size of the effect in terms meaningful to clinicians and patients.	2
**DISCUSSION**		
9. Strengths and Limitations of evidence:	Brief summary of strengths and limitations of evidence (e.g. inconsistency, imprecision, indirectness, or risk of bias, other supporting or conflicting evidence)	2
10. Interpretation:	General interpretation of the results and important implications	2
**OTHER**		
11. Funding:	Primary source of funding for the review.	20
12. Registration:	Registration number and registry name.	NA
